# Tissue-specific expression of histone H3 variants diversified after species separation

**DOI:** 10.1186/s13072-015-0027-3

**Published:** 2015-09-17

**Authors:** Kazumitsu Maehara, Akihito Harada, Yuko Sato, Masaki Matsumoto, Keiichi I. Nakayama, Hiroshi Kimura, Yasuyuki Ohkawa

**Affiliations:** Department of Advanced Medical Initiatives, Faculty of Medical Sciences, JST-CREST, Kyushu University, Fukuoka, 812-8582 Japan; Department of Biological Sciences, Graduate School of Bioscience and Biotechnology, Tokyo Institute of Technology, Yokohama, 226-0026 Japan; Department of Molecular and Cellular Biology, Medical Institute of Bioregulation, Kyushu University, 3-1-1 Maidashi, Higashi-ku, Fukuoka, Fukuoka 812-8582 Japan

## Abstract

**Background:**

The selective incorporation of appropriate histone variants into chromatin is critical for the regulation of genome function. Although many histone variants have been identified, a complete list has not been compiled.

**Results:**

We screened mouse, rat and human genomes by in silico hybridization using canonical histone sequences. In the mouse genome, we identified 14 uncharacterized H3 genes, among which 13 are similar to H3.3 and do not have human or rat counterparts, and one is similar to human testis-specific H3 variant, H3T/H3.4, and had a rat paralog. Although some of these genes were previously annotated as pseudogenes, their tissue-specific expression was confirmed by sequencing the 3′-UTR regions of the transcripts. Certain new variants were also detected at the protein level by mass spectrometry. When expressed as GFP-tagged versions in mouse C2C12 cells, some variants were stably incorporated into chromatin and the genome-wide distributions of most variants were similar to that of H3.3. Moreover, forced expression of H3 variants in chromatin resulted in alternate gene expression patterns after cell differentiation.

**Conclusions:**

We comprehensively identified and characterized novel mouse H3 variant genes that encoded highly conserved amino acid sequences compared to known histone H3. We speculated that the diversity of H3 variants acquired after species separation played a role in regulating tissue-specific gene expression in individual species. Their biological relevance and evolutionary aspect involving pseudogene diversification will be addressed by further functional analysis.

**Electronic supplementary material:**

The online version of this article (doi:10.1186/s13072-015-0027-3) contains supplementary material, which is available to authorized users.

## Background

Genomic DNA in eukaryotes is stored in nuclei as a highly packed structure called chromatin. The basic unit of chromatin is the nucleosome, in which DNA is wrapped around combinations of the core histone proteins H2A, H2B, H3 and H4. Each histone protein has several variants based on amino acid substitutions. In mice, canonical histone H3.1 and H3.2 are encoded by multiple genes gathered in three histone cluster*s* on chromosome 3, 11 and 13 [1]. In addition, some open reading frames (ORFs) similar to histone H3 have been annotated as pseudogenes because expression from these genes has not been determined. H3.1 coding genes produce RNA with a stem-loop in the 3′-end of the RNA structure instead of a poly-A tail and express no introns. In contrast, H3.3 is encoded by two genes, *H3f3a* on chromosome 1 and *H3f3b* on chromosome 11. These genes have a poly-A tail (signal), are located away from the histone cluster and expressed throughout the cell cycle in a replication-independent manner [2]. Specific histone variant incorporation into chromatin has been shown to play important roles in gene regulation during development and differentiation [3, 4].

The differential functions of individual histone variants are characteristic of various processes, such as nucleosome stability, protein binding, and chromatin modification. For example, H3.3 is generally distributed on transcriptionally active genes and harbors modifications associated with activation, such as H3K4me3, while H3.1 and H3.2 are distributed throughout the rest of the genome and are associated with inactivation modifications [5–8]. H3.3 is also known to be incorporated into promoter regions prior to transcriptional activation in cell differentiation by histone chaperone complexes, including HIRA and Chd1 [4, 9, 10]. Interestingly, H3.3 is also involved in genome silencing by being incorporated into pericentromeric heterochromatin and telomeric regions, in association with DAXX/ATRX [9, 11, 12]. In these cases, the selective incorporation of histone variants could be a molecular platform for downstream modifications and chromatin remodeling to acquire differentiation potential.

The discovery of new histone variants has been ongoing in numerous species [13, 14], and many histone-like sequences have been annotated as pseudogenes in genome databases [15]. Here, we report the identification and characterization of previously unknown histone genes in the mouse genome. By cross-hybridization analysis in silico (in silico hybridization), we have identified 14 uncharacterized histone H3 genes and 1 uncharacterized histone H2A gene that potentially encode histone proteins with core domains. Most of the new variants were not conserved in human, suggesting that these minor variants diverged after species separation. The expression of some of the new variants at the mRNA level was confirmed by 3′-seq analysis. When expressed as GFP-tagged forms in mouse C2C12 skeletal myoblasts, some variants were incorporated into chromatin and others were not. Whole transcriptome analysis revealed that the forced expression of any variant did not affect global transcription in undifferentiated myoblasts, but did upon myoblast differentiation. These diverse histone variants might play a role in regulating tissue-specific gene expression.

## Results

### Fourteen novel H3 genes identified by in silico hybridization

**Fig. 1:**
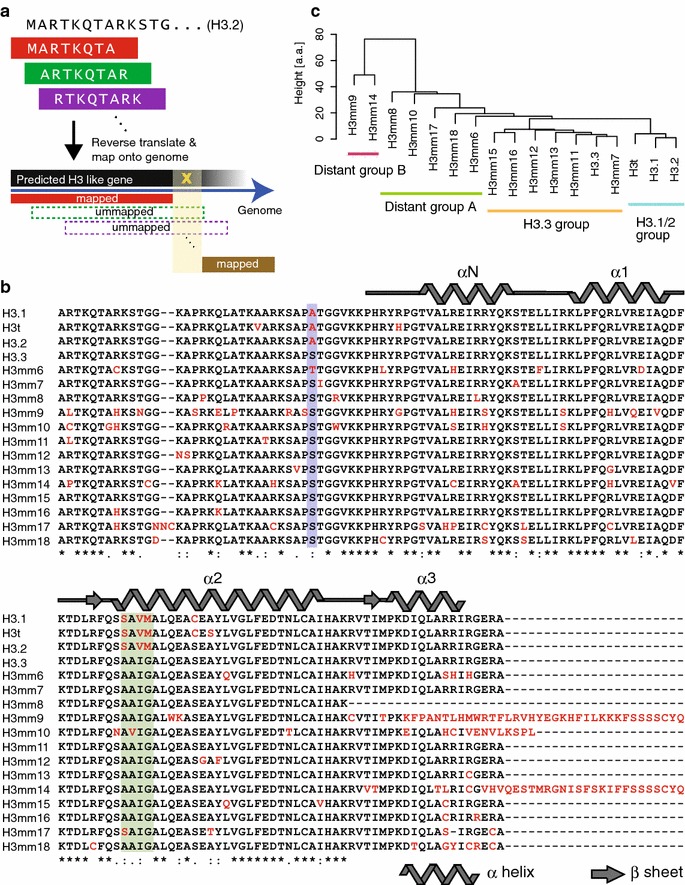
Identification of novel mouse H3 variants. **a** Schematic drawing of the H3 variant gene screening by in silico hybridization. We first split known H3 (H3.2) amino acid sequences into short fragments of eight amino acids and listed all possible combinations of fragments with respect to the codon table. Next, DNA sequences were mapped onto the mouse genome. Adjacently mapped sequences (<90 bp) were concatenated, and the concatenated regions were considered H3 variant coding genes. **b** Protein sequences of novel H3 variants. The protein sequences were translated from predicted H3 variant genes. Characteristic amino acids are in *red* type. The *blue* highlighted region indicates position 31 and the *green* highlighted region indicates amino acids at the N-terminal tail and the motif that discriminate H3.1 and H3.3. **c** Clusters of similar mouse H3 variant proteins. The cluster dendrogram was constructed by hierarchical clustering of H3 variant amino acid sequences. The edit distance (Levenshtein distance) was used as a similarity measure. Height (*y*-axis) indicates the edit distance between cluster pairs. Clusters containing H3.1 or H3.3 were defined as H3.1 and H3.3 groups, respectively. Other clusters distant from the H3.3 sequence were defined as distant groups A or B

To identify all genes encoding histone variants, we searched the mouse genome database for histone genes by in silico cross-hybridization screening (Fig. 1a). From the H3.2 amino acid sequence (CAA56577.1), eight amino acid sequence blocks (129 in total) were generated by shifting the sequence one amino acid (Fig. 1a). Each amino peptide sequence was reverse-translated based on mammalian codon usage, and 4,162,752 DNA sequences 24 nucleotides (nt) long were determined. Each DNA sequence was mapped onto the mouse genome (mm9) using Bowtie (with options: −a), and a total of 168,299 sequences (4.04 %) were successfully mapped, including multi-hit sequences. The mapped reads were considered concatenated sequences that encode histone proteins when two or more different sequences were mapped within 90 nt of each other. The connected sequences were filtered by eliminating sequences encoding peptides of less than 10 amino acids. This resulted in 87 genomic sequences that potentially represent histone H3 coding genes. These sequences contained known H3.1, H3.2 and H3.3 coding genes, 17 computationally predicted genes and 26 unannotated histone H3 pseudogenes and disrupted open reading frames (ORFs) (Additional file 1: Table S1). All previously identified histone H3 genes, regardless of the presence or absence of introns, were included. Because the core domain is essential for forming the nucleosome, we excluded ORFs that had out-of-frame core domains. This analysis resulted in the identification of 14 genes that potentially encode histone H3-like proteins (Fig. 1b; see Additional file 2: Figure S1 for DNA sequences). To assess whether these histone H3 sequences are conserved between mouse and human, we repeated the screen on the human genome (hg19). We extracted 24 ORFs that potentially encode a total of 11 histone H3 or H3-like proteins, but all 24 ORFs were previously known or predicted genes [16] and none were identical to the new mouse genes (Additional file 3: Table S2 for human genome screening results). We also examined the screen on the rat genome (rn5), which is taxonomically close to that of mouse. We extracted ORFs that potentially encode histone H3 or H3-like proteins (Additional file 4: Table S3). Only *Hist3h3*, which is deposited as provisional gene on rat genome, was extracted as the homolog of mouse H3:00036 (prediction ID), while other rat homologs of mouse H3 variants were not identified. Since the identical homologs at amino acid level among species, we further performed phylogenic analysis to evaluate conservation of histone H3 variants amino acid sequences, including those newly identified mouse H3 sequences. The phylogenetic tree showed that the 14 novel mouse histone H3 variants are categorized into two well-delineated clades (H3.1/H3.2 clade and H3.3 clade) (Additional file 2: Figure S2A). Thirteen novel H3 variants were categorized into the H3.3 clade (Additional file 2: Figure S2B) and only H3:00036 was placed in the H3.1/H3.2 clade (Additional file 2: Figure S2C). The 13 H3.3-related variants were not identical to any known histone genes in other species, including newly determined sequences in human and rat. Phylogenetic analysis also indicated that there is no obvious human ortholog of any of the mouse variants (Additional file 2: Figure S2B). These results suggested that the novel histone genes might be mouse specific; therefore, we named them H3mm (H3 *Mus musculus*) with a numbered suffix (i.e., H3mm6–H3mm18) to avoid future confusion, as the phylogeny-based nomenclature system [17] cannot be applied. Mouse H3:00036 and rat *Hist3h3*, however, might be counterparts of human H3T/H3.4 (see Additional file 2: Figure S3A for the sequence alignment of H3.1, H3T and H3t). We named this gene *H3t*, as both the genome structure and the characteristics of the encoded protein were similar to human H3T (as indicated below), in addition to the relevance in phylogeny [17]. While H3mm6–H3mm18 were similar to H3.3 and their genes were scattered outside the histone gene clusters, H3t was similar to H3.1 and its gene was located in histone cluster 2. *H3t* also had a stem-loop sequence in its 3′-UTR (see summary in Table 1).

**Table 1:** Genomic locations of novel H3 variants

Prediction ID	H3 variant name	Provisional gene name	Most similar protein	Locus (strand)	3’-end structure
H3:00003	H3mm8	*Gm8029*	histone H3.3	chr1:180229526-180230057 (+)	Poly-A
H3:00007	H3mm11	*H3f3c*	PREDICTED: histone H3.3-like	chr2:119491361-119491763 (−)	Poly-A
H3:00014	H3mm12	*Gm12657*	predicted gene 12657	chr4:94266075-94266486 (+)	Poly-A
H3:00033	H3mm10	*Gm3835*	histone H3.3	chr8:74953426-74953822 (−)	Poly-A
H3:00036	H3t	*Gm12260*	histone H3.1	chr11:58775313-58775724 (+)	Stem-loop
H3:00037	H3mm17	*Gm12271*	PREDICTED: histone H3.3-like	chr11:61229903-61230317 (−)	Poly-A
H3:00061	H3mm18	*Gm6132*	histone H3.3	chr13:70146081-70146492 (−)	(Not detected)
H3:00063	H3mm13	*Gm10257*	PREDICTED: histone H3.3-like	chr13:101716359-101716770 (−)	Poly-A
H3:00064	H3mm14	*Gm4938*	PREDICTED: histone H3.3-like	chr13:105708858-105709338(+)	Poly-A
H3:00065	H3mm7	*Gm6421*	histone H3.3	chr13:118147018-118147429 (−)	Poly-A
H3:00066	H3mm6	*Gm6128*	histone H3.3	chr14:21675232-21675643 (−)	Poly-A
H3:00074	H3mm15	*H3f3a*-*ps2*	histone H3.3	chr16:91114433-91114844 (−)	Poly-A
H3:00078	H3mm9	*Gm14529*	PREDICTED: histone H3.3-like	chrX:19916425-19916905 (+)	Poly-A
H3:00085	H3mm16	*H3f3a*-*ps1*	histone H3.3	chrX:99019853-99020264 (+)	Poly-A

The putative histone H3 proteins were categorized into four groups based on the edit distance (Levenshtein distance), which is a similarity measure defined for two sequences (words) that requires a minimum number of operations (delete, insert or substitute) to transform one into the other. All proteins showed high homology with H3.3 (percent identity ranged from 76 to 98.5 %; see also Additional file 5: Table S4 for amino acid sequences and DNA sequences similarities), but could be separated into four groups. One group, the H3.1/2 group, included H3.1, H3.2 and H3t and contained the SAVM (87–90) motif in the histone core domain. The other three groups (H3.3, distant A, and distant B) all had the AAIG (87–90) motif, which is recognized by ASF1/HIRA or Daxx [18–20], except for H3mm10 and H3mm17, which instead have AVIG and SAIG sequences, respectively. Compared with H3.3, the number of different amino acids was less than five in the H3.3 group, but was 11–23 or 37–51 amino acids in the distant A or B groups, respectively. (Figure 1c; see also Additional file 5: Table S4).

Lysine residues in the histone H3N-terminal tail region are known to be post-translationally modified as part of the mechanism for chromatin regulation [21]. The important lysines that are subjected to acetylation and methylation, including K4, K9, K27 and K36, were conserved among all histone H3 genes. Similarly, phosphorylatable serine residues (S10 and S28) were conserved among all genes, except H3mm9, where amino acid 28 was arginine. Variations in the N-terminal tail region were found around these critical lysine and serine residues and may regulate the modification levels in specific variants. On the other hand, variation in the core domain may alter the nucleosome structure and/or stability.

The edit distance at the nucleotide level unveiled extremely high homology between *H3f3a* and some of the newly identified genes (73.7–99.3 %; Additional file 5: Table S4). For example, the edit distance was 3 nt for *H3mm7*, 4 nt for *H3mm11* and *H3mm13*, and 5 nt for *H3mm15*. Such high similarity may have hindered previous attempts to identify such genes. The edit distance from *H3f3b* demonstrated that *H3mm8* is more similar to *H3f3b* (68 nt) than *H3f3a* (141 nt). These similarities indicate that the novel H3 genes are potentially derived from either *H3f3a* or *H3f3b*. The genomic structure of the novel genes predicted a polyadenylation signal and no stem-loop structure, except for *H3t*.

We also applied the same strategy to the other core histones, H2A, H2B and H4. H2A genes were screened with H2A type1B protein (NP_835489.1). The screening revealed one uncharacterized gene that encodes a protein similar to *H2A.J* (Additional file 2: Figure S3B; Table S5). H2B genes were screened with H2B type1P (NP_835509.2) and H4 genes with H4 (NP_78583.1), but neither screen resulted in the identification of a previously unknown ORF. These results suggest that histone H3 genes are more diverse than the other histone genes in the mouse genome.

### The expression of novel H3 genes in mouse tissues

**Fig. 2:**
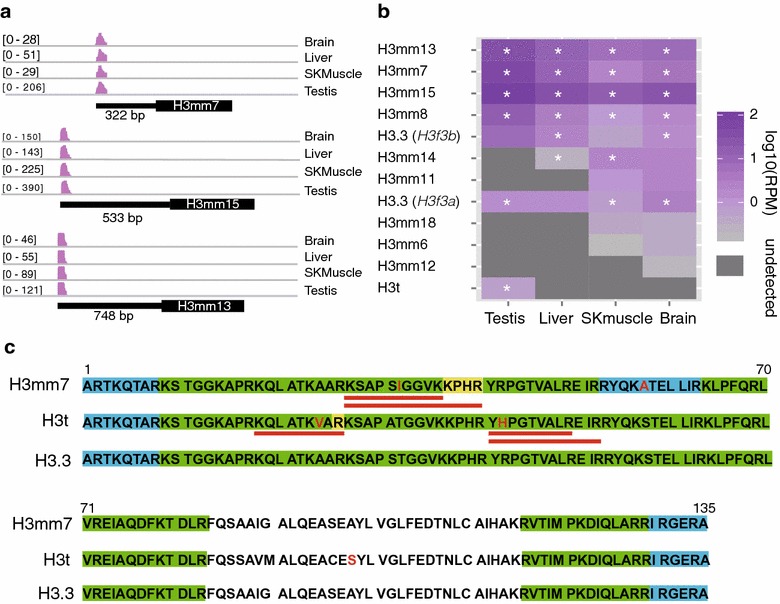
H3 variant genes are expressed in mouse tissues. **a** Representative IGV screenshots of 3′-seq data of the four tissues. The mRNA sequences mapped in four tissues (brain, liver, skeletal muscle and testis) are shown in *four lanes* at each locus of the H3 variants. The *black thick bars* indicate exons, and *black thin bars* indicate the predicted 3′-UTR of each H3 variant gene. The predicted lengths of the 3′-UTRs are shown below the genes. Piled-up depths are shown as (min–max) to the *left* of each *lane*. **b** The gene expression levels of novel H3 variants in four tissues. The expression levels of H3 variants (log_10_ RPM) are indicated by the *color bar* to the *right*. Tiles marked with *white asterisks* confirm H3 variant expression (RPM > 0) in the replicated data. **c** Identified peptides and their positions in histone sequences. LC–MS/MS analysis was performed using acid extracts from adult male mouse tissues (8 week old), as shown in Additional file 2: Figure S8A. *Red letters* mark amino acids specific to H3t and H3mm7. *Red underline* indicates unique peptide sequences. *Green*, *yellow* and *light blue* indicate peptides in the peptide sequence with high confidence, medium confidence and low confidence, respectively

To evaluate the expression level of each H3 gene, we first analyzed the standard mRNA-Seq data obtained from either public data sets, including those from ENCODE, and local data sets. However, those data sets were not adequate for quantification because the coding regions of the H3 variants were very similar and the number of uniquely mapped reads was very low (Additional file 2: Figure S4, 5). Such high similarities prevented us from performing reliable RT-PCR. ChIP-seq data for active histone marks and RNA polymerase II could have been useful to evaluate the transcription level of each variant; however, this was also difficult because the promoter regions were also very similar among the different variants, and the depths of uniquely mapped sequences were not sufficient for quantification (Additional file 2: Figure S5A–P). Consequently, we performed 3′-seq to identify their 3′-UTRs [22] (Fig. 2a), because 3′-UTRs showed relatively greater difference in nucleotide sequence compared with coding sequence (Additional file 2: Figure S6). 3′-seq expression profiles in mouse tissues (testis, liver, skeletal muscle and brain) showed that *H3mm7*, *H3mm8*, *H3mm13* and *H3mm15* were expressed in all four tissues, whereas the expression of *H3mm6*, *H3mm11*, *H3mm12*, *H3mm14* and *H3mm18* was biased for specific tissues (Fig. 2b; see also Additional file 2: Table S6). The expression level of *H3t* was low, but specifically detected in the testis. The expression levels of *H3mm7*, *H3mm8*, *H3mm13* and *H3mm15* were in fact similar or higher (1.2- to 16-fold) compared with those of *H3f3a* and *H3f3b* (Additional file 2: Figure S7).

We next used liquid chromatography tandem mass spectrometry (LC–MS/MS) to investigate the expression of new H3 variants at the protein level. Histones were acid-extracted from mouse adult tissues and separated by SDS-PAGE, before in-gel digestion and LC–MS/MS analysis (Additional file 2: Figure S8A). 72 peptides were identified to be derived from histone H3. Although most were shared among multiple variants, 14 peptides were specific to one of the novel H3 variants (H3mm6, H3mm7, H3mm9, H3mm13, H3mm17 or H3t). We then quantified the variant specific peptides corresponding to H3t and H3mm7, which were detected with high confidence (false discovery rate <0.05), as the area of the precursor ion chromatogram normalized to the area of a common histone peptide (Fig. 2c; Additional file 2: Figure S8A–D). These data suggested that the novel variants were significantly expressed both at mRNA and protein levels.

### Some novel H3 variants are stably incorporated in chromatin

To elucidate the functions of the novel histone H3 proteins described above, we forced all H3 variants in Fig. 1b in mouse C2C12 myoblast cells that endogenously express *H3mm7*, *H3mm8*, *H3mm13* and *H3mm15* (Fig. 2a, b; Additional file 2: Table S6). We established stable cell lines in which the expression of each N-terminal GFP-tagged histone H3 protein can be induced by doxycycline (Dox).

**Fig. 3:**
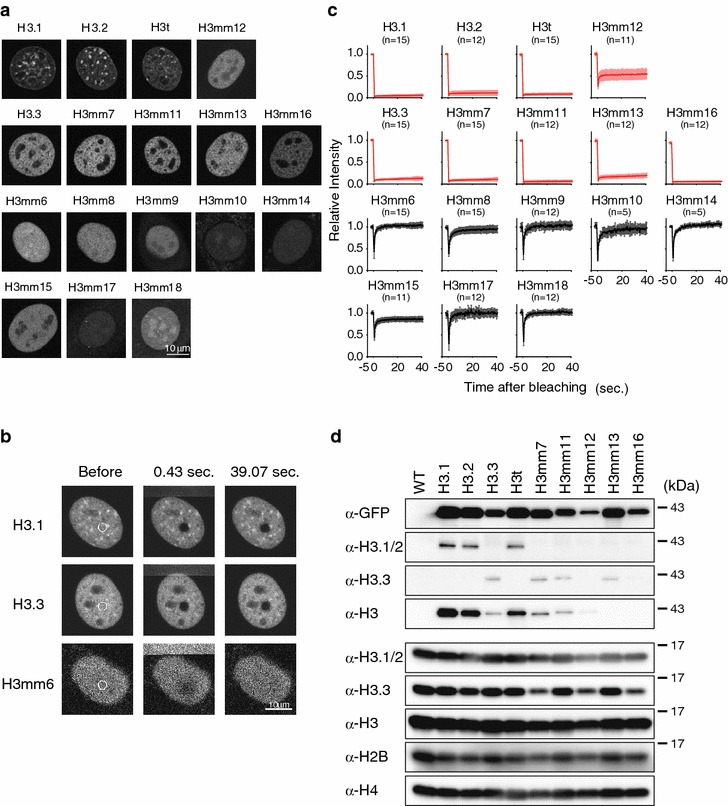
Six of 14 novel H3 variants incorporate into chromatin. **a** Images of GFP-tagged histone H3 variants show localizations in nuclei that were categorized into three groups. Variant expression was induced by tetracycline. *Scale bar* 10 μm. **b** Fluorescence recovery after photobleaching experiments to evaluate the nucleosomal stability of H3 variants. The areas marked with *white circles* were photobleached, and the recovery of the respective fluorescent signals was monitored (selected time points are shown). **c** GFP-tagged histone H3 variants were categorized into two groups: those that were stably incorporated into nuclei and those that were not. The mobility of the GFP-fused histone H3 variants was analyzed based on fluorescence recovery after photobleaching. Recovery curves of GFP-fused histone H3 variants are shown. Relative fluorescence intensities are mean ± standard deviation (*n* represents the number of trials). The rate of fluorescence recovery indicates the stability of the chromatin incorporation. *Red lines* show stable incorporation for GFP-H3.1, H3.2, H3.3, H3t, H3mm7, H3mm11, H3mm12, H3mm13 and H3mm16. *Black lines* show diffusion in the nucleus for GFP-H3mm6, H3mm8, H3mm9, H3mm10, H3mm14, H3mm15, and H3mm18. *Scale bars* 10 μm. **d** Exogenously expressed GFP-tagged histone H3 variants did not interfere with the endogenous expression of core histones. Immunoblots were performed using acid extract from GFP-tagged histone H3 variant-expressing C2C12 cells in the undifferentiated state. The *top four panels* show exogenously expressed GFP-tagged histone H3 variants; the *lower five panels* show the endogenous level of each core histone. Wild-type C2C12 cells (WT) were used as a control (no expression of GFP-tagged histones)

We first investigated the distribution of GFP-H3 variants. H3.1, H3.2, and H3t exhibited typical chromatin patterns with obvious heterochromatic foci (Fig. 3a). H3.3 and four H3.3-type variants (H3mm7, H3mm11, H3mm13 and H3mm16) exhibited euchromatic distributions. H3mm12 was also distributed like H3.3, but with higher diffusible background staining. Others (H3mm6, H3mm8, H3mm9, H3mm10, H3mm14, H3mm15, H3mm17 and H3mm18) showed nearly homogenous distribution throughout nuclei, suggesting that these variants diffuse freely. We next examined chromatin incorporation of H3 variants using fluorescence recovery after photobleaching (FRAP) (Fig. 3b). As expected, most variants showing chromatin distribution did not recover within 40 s after bleaching (H3.1, H3.2, H3.3, H3t, H3mm7, H3mm11, H3mm13 and H3mm16; Fig. 3c), consistent with stable chromatin incorporation. H3mm12 showed partial recovery because of two fractions: half diffused rapidly and the other half was stable. H3 variants that exhibited nearly homogenous distributions showed rapid recovery, indicating they diffuse rapidly. A chromatin-incorporated fraction of H3mm15 may also exist, as it did not recover to the original level. GFP-tagged H3 variants that showed stable association with DNA (i.e., H3t, H3mm7, H3mm11, H3mm12, H3mm13 and H3mm16) were also concentrated on condensed chromosomes in mitotic cells, as were H3.1, H3.2 and H3.3 (Additional file 2: Figure S9). H3mm15, which showed an incomplete recovery by FRAP, was also enriched in mitotic chromosomes over the cytoplasmic diffuse background. These results suggested that H3t, H3mm7, H3mm11, H3mm13 and H3mm16 are stably incorporated into chromatin. To evaluate the chromatin incorporation biochemically, we prepared mononucleosomes from cells expressing the GFP-H3 variants using micrococcal nuclease (MNase) digestion and hydroxyl apatite (HAP) purification, before immunoprecipitating GFP-H3-containing nucleosomes. Both GFP-H3t and GFP-H3mm7 were found in mono-nucleosome fractions together with other core histones, as were GFP-H3.1 and GFP-H3.3. Besides, H3mm14, which is chromatin non-incorporable variant (Fig. 3c), was not found in the fraction as expected (Additional file 2: Figure S10). These results supported the view that the H3 variants are stably bound to chromatin and that mitotic chromosomes are incorporated into nucleosomes.

To examine whether H3 variants are incorporated into chromatin in a replication-dependent or -independent manner, we performed cell fusion assays [23]. C2C12 cells expressing GFP-H3 were fused with HeLa cells expressing mCherry-PCNA. One hour later, cells were fixed, and the distribution of GFP-H3 was analyzed by confocal microscopy. If the GFP-H3 that entered into recipient HeLa nuclei in heterokaryons was incorporated into replicated chromatin, the distribution should be associated with mCherry-PCNA-positive replication foci [24]. In contrast, if chromatin incorporation of GFP-H3 was replication-independent, the distribution should not be associated with replication foci. As shown in Additional file 2: Figure S11, GFP-H3.1 and GFP-H3t were concentrated in mCherry-PCNA foci in recipient nuclei, whereas GFP-H3.3 and -GFP-H3mm7 were not. These results are consistent with the similarity of H3t and H3mm7 with H3.1 and H3.3, respectively, indicating that H3t is incorporated into chromatin in a replication-dependent manner, like H3.1, and that H3mm7 incorporation is replication-independent, like H3.3.

Interestingly, these results were consistent with the edit distance analysis. H3 variants classified into the H3.1/2 group (i.e., H3.1, H3.2, and H3t) were incorporated into chromatin, including heterochromatin. H3 variants classified into the H3.3 group were incorporated into euchromatin, except H3mm15, which is mostly mobile. In contrast to these variants, which are close to the major H3 variants (H3.1, H3.2 and H3.3), those classified into the distant A and B groups (Fig. 1c) were not stably incorporated into chromatin.

During the above microscopy analyses, we noticed that fluorescence intensities varied among different variants: those variants incorporated into chromatin generally gave bright fluorescence signals. Immunoblotting with anti-GFP confirmed this observation (Additional file 2: Figure S12A). H3 variants that were incorporated into chromatin were readily detected, but nucleosome-free variants were not, whereas their transcript levels were similar or higher when evaluated by RT-qPCR using a primer set that amplified the shared GFP region (Additional file 2: Figure S12B). These data suggested that nucleosome-free histones undergo rapid turnover [25–28]. This notion was confirmed by proteasome inhibitor treatment. When cells were incubated with a proteasome inhibitor, MG132, for 6 h, the levels of nucleosome-free H3 variants, such as GFP-H3mm14 and GFP-H3mm18, were massively increased (Additional file 2: Figure S13). Compared with GFP-H3.3, the levels of GFP-H3.1 and GFP-H3t were also increased. This could be because the cell cycle-independent expressions of these GFP-H3 proteins, unlike the endogenous H3.1; GFP-H3.1 and GFP-H3t expressed in non-S-phase cells, perhaps undergo degradation, as their chromatin incorporation is limited without DNA replication.

### The novel H3.3-type histones were preferentially enriched at active genes

**Fig. 4:**
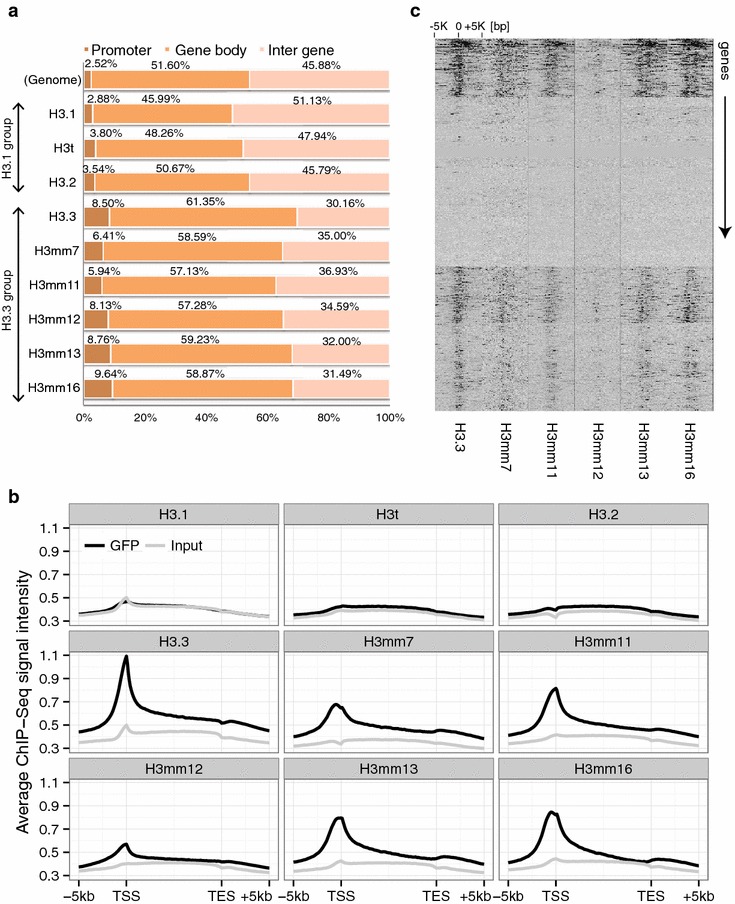
Genome-wide distributions of novel H3 variants are classified into H3.1-like or H3.3-like patterns. **a** Proportion of each incorporated H3 variant localized in three gene regions (promoter, gene body or inter-gene). The proportions were calculated from the ratio of ChIP-Seq peaks detected in the categories. The *top lane* (Genome) indicates the proportion (%) of each category with random (uniform) distribution on the genome to enable comparison. The other *lanes* are proportions of detected peaks for the H3.1 and H3.3 groups. **b** The distribution of H3 variants focused on gene loci. Data for the H3.1-type variant is shown as a control. The *x*-axis shows the relative coordinate of the gene from the TSS to the TES (transcription end site). The *y*-axis shows the average ChIP-Seq and the input signal intensity over all mouse genes. **c** Distribution of H3.3-type variants around the TSSs of all genes. The order of genes (*rows*) was determined by hierarchical clustering using total log_2_FCs within 5 kb from the TSSs. Higher fold-change (FC) is indicated by *thicker black shading*

To gain insight into the function of novel H3 variants, we analyzed the genome-wide distribution of the histone variants that are incorporated into chromatin (H3t, H3mm7, H3mm11, H3mm12, H3mm13 and H3mm16) by ChIP-Seq using GFP antibody and then compared these distributions with those of H3.1, H3.2 and H3.3. The genome-wide distributions of the GFP-tagged histone H3 were evaluated by calculating the proportion of peaks detected on each category (promoter, gene body and inter-gene in Fig. 4a) by MACS software [29] with the relaxed threshold and with the broad-calling option as previously utilized by Hussein et al. [30] to call dispersed histone modification peaks. In the mouse genome, the effective mappable genome size of mm9 is 1,865,500,000 bp defined in MACS, with promoter regions (within 2 kb of a transcription start site; TSS) occupying 2.52 % and gene body regions occupying 51.60 % (962,779,619 bp; 23,460 genes defined in refFlat) (Fig. 4a, top lane). Peak call data obtained from ChIP-Seq revealed that H3t was distributed uniformly, much like the distribution of H3.1 and H3.2, because the proportions of peaks in the promoter regions (2.52–3.80 %) and gene bodies (45.99–50.67 %) of these three variants were similar to the proportions in randomly chosen genomic regions (Fig. 4a, top lane). The uniform distributions of H3.1, H3.2 and H3t probably represent the replication-coupled chromatin assembly. In contrast, H3mm7, H3mm11, H3mm12, H3mm13 and H3mm16 were specifically localized in gene loci with peaks in promoter regions (5.94–9.64 %) and gene bodies (57.13–61.35 %). This property is similar to that of H3.3, which is specifically localized in gene loci [9, 31].

To evaluate the local distribution of the novel H3 variants in gene loci, aggregation plots were created (Fig. 4b). Compared to input control data, none of H3.1, H3t or H3.2 accumulated around TSSs. H3mm7, H3mm11, H3mm12, H3mm13 and H3mm16 were enriched near TSSs, similar to H3.3. These results suggest that these H3.3-type variants may have a similar role to H3.3 in selective gene expression. To investigate this possibility, we assessed the biased localization of the ChIP-Seq signal within ±5000 bp at all gene TSSs in the growth states by hierarchical clustering (Fig. 4c). None of H3mm7, H3mm11, H3mm13 or H3mm16 showed any remarkable exclusivity in signal localization compared with H3.3. H3mm12 was less concentrated in gene loci, but still had an incorporation pattern similar to that of H3.3.

### Overexpression of novel histone H3 variants modulates gene expression patterns during differentiation

To evaluate the function of chromatin-incorporated H3 variants with respect to gene expression during differentiation, we performed mRNA-Seq analysis before and after the differentiation of C2C12 cells that stably express the variants. During the growth state, gene expression profiles were similar, with correlation coefficients 0.88–0.99 (Additional file 2: Table S7). In contrast, when cells were differentiated, the profiles were more diverse, depending on the variant (correlation coefficient 0.79–0.98; Additional file 2: Table S7), indicating that the overexpressed specific variants have the potential to alter gene regulation during differentiation, as do H3.1 and H3.3 [10].

**Fig. 5:**
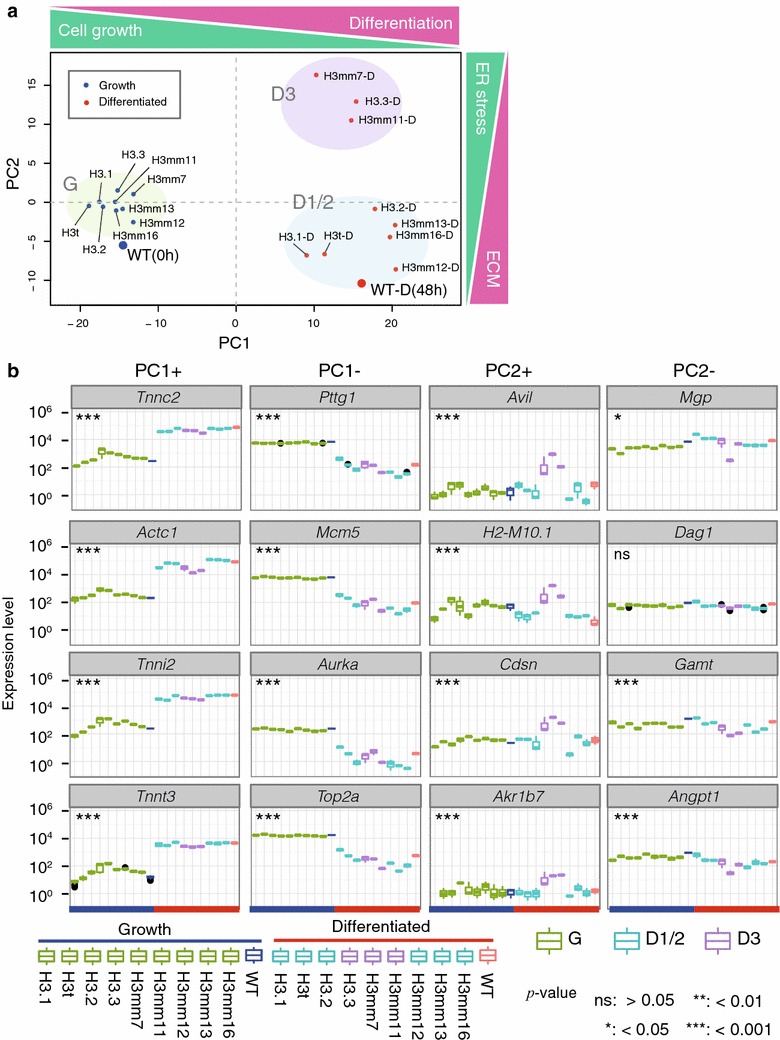
Histone variants correlate with two differentiation states. **a** Principal component analysis of gene expression patterns in H3 variant-overexpressing C2C12 cell differentiation. The *x*- and *y*-axes indicate the PC1 and PC2 scores, respectively, of the gene expression profiles in each cell. *Blue points* indicate the growth state; *red points* indicate the differentiated state. The distance between two points reflects the dissimilarity in gene expression patterns between cells. The higher PC1 score (PC1+) indicates a higher expression of muscle differentiation-related genes and lower expression of cell growth-related genes, as illustrated in the *top bar*. Similarly, higher PC2 scores (PC2+) indicate higher expression of ER-stress-related genes and lower expression of extracellular matrix (ECM)-related genes. Clusters of cells that have similar gene expression patterns, G (*green*), D1/2 (*blue*) and D3 (*purple*), are highlighted. **b** Gene expression levels of each H3 variant confirmed by RT-PCR amplicon-Seq. Expression levels of representative genes chosen from the top four contributors (genes) for each PC direction (PC1± and PC2±) are shown as *boxplots* calculated from three replicates. The illustration below shows the order of each H3 variant-expressing cell line in the growth and differentiated state. The *color* of *each box* corresponds to the expression groups shown in **a**: *blue*, undifferentiated wild-type (WT); *red*, differentiated WT; *green*, growth (G); *blue*, D1/2; and *purple*, D3. Two-sided Student’s *t* test was performed on group-average expression levels between “WT” vs. “Differentiated” for PC1± genes, and between “D1/2” vs. “D3” groups for PC2± genes

We next classified the gene expression profiles of different cell lines under growth and differentiation conditions by principal component (PC) analysis (Fig. 5a). The first principal component (PC1) had a large contribution rate (64.68 %). A positive PC1 score indicates gene expression patterns typical of skeletal muscle differentiation, whereas a negative score indicates the expression of cell growth-related genes based on gene set enrichment analysis (GSEA) of the top 100 high-scored genes using the REACTOME database [32, 33]. PC1 scores of all cells, including wild type, increased upon differentiation. Positive PC2 scores indicate higher expression of ER-stress-related genes, while negative PC2 scores indicate higher expression of extracellular matrix-related genes, according to GSEA. Group D1/2 (differentiated cells expressing H3.1 and H3.2; PC2 negative scores) included wild type and cells expressing H3t, H3mm12, H3mm13 and H3mm16, while group D3 (differentiated cells expressing H3.3; PC2 positive scores) included those expressing H3mm7 and H3mm11 (Fig. 5a). These data suggest that overexpression of any particular H3 variant has little effect on gene expression in the undifferentiated state but that upon differentiation overexpression of some variants will alter gene expression patterns. Because ER-stress-related genes are thought to have a special role in the efficient formation of myofibers during skeletal muscle differentiation [34], the D3 group might represent histone variants involved in the maturation of skeletal muscle differentiation.

To evaluate the expression levels of genes that contributed highly to PC scores (top four genes), we performed RT-PCR amplicon sequencing with three biological replicates for each gene (Fig. 5b). In all cells after differentiation, PC1-positive *Tnnc2*, a skeletal muscle differentiation-related gene, was upregulated, whereas PC1-negative *Pttg1*, a cell growth-related gene, was downregulated (Fig. 5b). Other PC1-contributing genes behaved similarly, consistent with cell cycle arrest upon differentiation [35]. We confirmed the statistical significance by a two-sided Student’s *t* test between average expression levels of growth and differentiation (Fig. 5b; *p* value <0.001 for all PC1 genes). PC2-positive *Avil* was upregulated in group D3. PC2-negative *Mgp*, however, did not show differential expression between the D3 and D1/2 groups, except for down-regulation of H3mm7 (*p* value <0.001; D1/2 vs. H3mm7-D), which may reflect milder negative PC2 scores (Fig. 5b; two-sided Student’s *t* test between the D1/2 and D3 groups). The results from PC analysis indicate that the expressions of H3.3, H3mm7 and H3mm11 during cell differentiation lead to changes in gene expression patterns that enhance differentiation.

The H3 variants in the D3 group could stimulate the expression of PC2-positive genes by being specifically incorporated into these genes. To test this possibility, we evaluated the level of incorporation of each variant around the TSSs (TSS ± 2 kb) of the top 40 PC2+ contributing genes (Additional file 2: Figure S14A). PC2+ genes were largely divided into two large clusters of H3.3-type histone incorporated or non-incorporated patterns, with the former not showing substantial differences between variants in the D1/2 and D3 groups. Nearly identical distributions of different variants on a specific PC2+ gene locus (*Cdsn*) were observed before and after differentiation (Additional file 2: Figure S14B). Thus, upon differentiation, the altered levels of expression could be involved in the incorporation of each variant, which depended on small amino acid differences.

The characterization of the novel mouse H3 variants is summarized in Table 2.

**Table 2:** Characterization of histone H3 variants incorporated into chromatin

H3 variant	AA alternation (from)	Motif 87–90	Edit distance [nt] (from)	mRNA/protein	Chromatin incorporable	Tissue specificity	Expression pattern	Distribution pattern
H3.1	S96C (H3.2)	SAVM	–	–	Yes	–	D1/2	Global
H3t	A24V, R42H, A98S (H3.1)	SAVM	52 (*Hist1h3a*)	Protein	Yes	Testis	D1/2	Global
H3.2	C96S (H3.1)	SAVM	10 (*Hist1h3a*)	–	Yes	–	D1/2	Global
H3.3	A31S, S87A, V89I, M90G (H3.2)	AAIG	–	–	Yes	–	D3	Local
H3mm6	#11 (H3.3)	AAIG	15 (*H3f3a*)	None	No	–	–	–
H3mm7	T32I, S57A (H3.3)	AAIG	3 (*H3f3a*)	Protein	Yes	SKM^a^, testis	D3	Local
H3mm8	#23 (H3.3)	AAIG	68 (*H3f3b*)	mRNA	No	–	–	–
H3mm9	#51 (H3.3)	AAIG	102 (*H3f3a*)	None	No	–	–	–
H3mm10	#23 (H3.3)	AVIG	32 (*H3f3a*)	mRNA	No	–	–	–
H3mm11	R2L, A25T (H3.3)	AAIG	4 (*H3f3a*)	None	Yes	–	D3	Local
H3mm12	K14N, A15S, E97G, Y99F (H3.3)	AAIG	6 (*H3f3a*)	None	Yes	–	D1/2	Local
H3mm13	A29V, R69G, R131C (H3.3)	AAIG	4 (*H3f3a*)	mRNA	Yes	–	D1/2	Local
H3mm14	#37 (H3.3)	AAIG	89 (*H3f3a*)	mRNA	No	–	–	–
H3mm15	L100Q, I112V, R128C (H3.3)	AAIG	5 (*H3f3a*)	mRNA	No	–	–	–
H3mm16	R8H, Q19K, R128C, G132R (H3.3)	AAIG	7 (*H3f3a*)	None	Yes	–	D1/2	Local
H3mm17	#16 (H3.3)	SAIG	24 (*H3f3a*)	mRNA	No	–	–	–
H3mm18	#12 (H3.3)	AAIG	17 (*H3f3a*)	None	No	–	–	–

^a^Skeletal muscle

## Discussion

We have identified 14 novel mouse histone H3 variants by in silico hybridization. Most of the H3 genes we identified could be computationally predicted genes using NCBI’s GNOMON pipeline [36] (GNOMON IDs have a “GM” prefix followed by a number, e.g. *GM12260*, which is equivalent to our *H3t*) and some of them are deposited as pseudogenes (H3t, H3mm7). This software uses a strategy similar to the one we employed; it splits known cDNA or peptide sequences into short fragments and scans the genome. The detection of *H3f3a* and *H3f3b* in the mouse genome confirmed that the software was applicable for genes with exon–intron structure. Our approach is more straightforward and comprehensive, and can be applied to any protein.

We also comprehensively identified histone H3 genes both in human and rat genomes. Phylogenetic analysis revealed that most histone H3 variants, except H3t, were not conserved even between mouse and rat. Histone genes have been suggested to have a various pseudogenes [15]. In our screen, many typical pseudogenes, which obviously lack the whole open reading frame by frameshift or insertion of a stop codon, were identified. Although some H3 variants reported here have been annotated as “pseudogenes”, at least H3mm7 and H3t should not be categorized as pseudogenes because their protein products were confirmed by LC–MS/MS. Recent studies have shown that some “pseudogenes” are constitutively or conditionally expressed at RNA level, which might also be translated [37, 38]. It is therefore questionable whether these are non-functional pseudogenes, newly evolved genes or DNA elements with specific functions. Although it has been difficult to determine the function of such potential pseudogenes, such as by making knockout mice or knockout cell lines, using the recently developed CRISPR/Cas9 genome-editing technology will allow us to address this question, in addition to determining the function of the variant per se. The set of new histone H3 variants will be a good target for understanding the mechanism of molecular evolution because a variety of (pseudo)gene types are present, including those with protein expression, those with RNA expression, those without expression, and those with ORF truncation. A deeper analysis of 3′-seq may also reveal the expression states of other “pseudogenes” than histones.

To characterize the properties of the individual variants, we established C2C12 cells expressing all 14 variants. FRAP analysis revealed that six variants were assembled into chromatin. One variant showed high similarity to H3.1 at both the DNA and amino acid sequence levels (H3t). The H3t gene is located in a histone cluster and has a stem-loop sequence in the 3′-UTR, similar to H3.1. In addition, GFP-H3t is distributed throughout the genome, again much like GFP-H3.1. Cell fusion analysis confirmed that H3t is incorporated in a replication-dependent manner, as is H3.1 [15]. Other chromatin-incorporated variants are similar to H3.3 in terms of gene structure (i.e., no stem-loop), amino acid sequence, and distribution. However, they can be separated into two distinct groups based on their effect on gene expression in differentiated cells. H3mm7 and H3mm11 alter gene expression patterns and increase the levels of ER-stress-related genes, much like H3.3, suggesting that they can contribute to gene selection and lineage potential, similarly to H3.3 [4, 10]. The functional difference between groups D1/2 and D3 might be explained by the unique amino acids in the N-terminal tail of H3 that affect post-translational modifications and/or structural differences in the nucleosome.

A number of new H3 variants did not appear to be incorporated into chromatin. In two variants (H3mm10 and H3mm17), amino acid substitutions in the histone chaperone-binding domain may explain the lack of chromatin incorporation because of poor binding to chaperones [18–20]. In contrast, the chaperone-binding domains are conserved in other non-incorporated variants, suggesting that they can interact with chaperones but that stable nucleosomes are not formed. Indeed, H3mm8 lacks the amino acids required for the C-terminal helix (α3 in Fig. 1b), and H3mm9 and H3mm14 have extended C-terminal amino acids, which may disrupt nucleosome structure. Although other variants (H3mm6, H3mm15 and H3mm18) do not have such large deletions or additions, amino acid substitutions in the histone fold domain may drastically alter the nucleosome stability, as has been shown for human H3T [39]. The function of these non-incorporated variants remains unknown. One possibility is that their transient interaction with chromatin may mediate chromatin remodeling. Another possibility is that they may sequestrate histone binding proteins, competing with chromatin-incorporated variants, like, for example, influenza virus protein NS1, which has a C-terminal tail that contains an ARSK sequence similar to the ARTK sequence in the N-terminal of histone H3 [40]. This NS1 region competes with histone H3 for interaction with transcription elongation factors to suppress the expression of anti-virus-related genes. Similar regulation might occur when unincorporated histones are expressed.

The novel histone variant genes except H3t were not conserved between human and mouse, unlike H3.1, H3.2 and H3.3. H3t has a high similarity to human H3T, sharing two common amino acids (Val 24 and Ser 98), and its expression in either species is testis specific [14, 39]. However, in contrast to human H3T, which forms unstable nucleosomes [39], FRAP analysis indicates that H3t stably assembles into nucleosomes. Moreover, amino acids that cause instability (Met 71 and Val 111 in human H3T) are not conserved in H3t. Rat *Hist3h3* encodes a protein with an identical amino acid sequence to mouse H3t. Further biochemical, structural, and genetic studies are required to elucidate the function of H3t. Other novel H3 variants do not have counterparts in human, suggesting that these minor histone variants were acquired after species separation. This theory supports the idea that H3 genes evolve according to a birth-and-death process [41]. It may be that the species-specific variants contribute to the establishment of species-specific gene regulation. Thus, functional differences among individual H3 variants should be addressed to understand the evolution of chromatin dynamics.

## Conclusions

We identified novel H3 variant genes in the mouse genome. Thirteen out of the 14 genes that appear to be derived from H3.3 are not conserved among species, including human and rat, even though tissue-specific expression was confirmed for some variants. Another one, H3t, an H3.1 type, showed replication-dependent chromatin incorporation, and appears to have human and rat counterparts. Forced expression of novel histone H3 variants affected gene expression patterns during myogenesis. Although the functions of these variants remain unknown, constructing knockout mice and cell lines will address their biological relevance and provide insight into the molecular evolution of pseudogene diversification.

## Methods

### Identification of novel H3 variants by in silico hybridization

Histone H3 variant genes in mouse were explored as shown in Fig. 1. First, 136 amino acids (a.a.) of the histone H3.2 sequence (CAA56577.1) were divided into 8 a.a. sequences in 1 a.a. iterations. The obtained 129 a.a. sequences were converted into all possible combinations of 24 nt DNA sequences based on mammalian codon usage. This conversion resulted in 4,162,752 DNA sequences that potentially code histone genes. The obtained DNA sequences were mapped onto the mouse genome (mm9) by Bowtie (version 0.12.7 with option −a to report all candidates). Ultimately, 168,299 DNA sequences were mapped, including multi-hit reads. The mapped DNA sequences were concatenated if more than two reads were mapped within 90 nt of each other. Eighty-seven regions shared homology to the H3.2 coding sequence, yielding 16 genes that potentially encode H3 histones.

### 3′-seq and 3′-seq data analysis

Sample preparations and data analysis for 3′-seq were performed as previously reported [22] using total RNA extracted from 8-week-old C57BL/6 male mouse tissue, including testis, liver, brain and skeletal muscle. Deep sequencing was performed using the Illumina Hiseq 1500 system. The 3′-seq yielded total reads of 26,880,228–42,145,105 for the tissue samples and 13,206,076 and 20,670,900 for C2C12 growth and differentiated cells. The uniquely mapped reads were 3,602,579–14,060,394 (11.23–36.65 %) for tissue samples and 539,990–3,952,088 (4.09–19.12 %) for C2C12 growth and differentiated cells. The number of unique mapped reads of ~1 to ~10 million were comparable to that reported by Lianoglou et al. [22]. Reads were mapped to the mouse genome (mm9) with STAR alignment software [42] and the parameter “–*outFilterMultimapNmax 1* –*alignIntronMax 1*” (no multi-hit reads, no splice prediction) to treat poly-A containing reads. Quantification of each gene was performed by counting the number of reads that were mapped in the 3′-UTR region and then normalizing the number as reads per million (RPM) per region. In the case of novel histone H3 variant genes, the region within 3 kb from the end of a coding sequence was defined as the putative UTR.

### In-gel digestion

Proteins were fractionated by SDS-PAGE (10–20 %) and stained with Coomassie Brilliant Blue G250 (CBB G250). Protein bands were cut out and subjected to in-gel digestion as described previously [43]. Obtained peptides were dried and stored at −80 °C.

### LC–MS/MS analysis

Liquid chromatography tandem mass spectrometry (LC–MS/MS) was performed on an LTQ Orbitrap Velos Pro mass spectrometer (Thermo Fisher Scientific, Waltham, MA, USA) coupled with a nanoLC instrument (Advance, Michrom BioResources, Auburn, CA, USA) and an HTC-PAL autosampler (CTC Analytics, Zwingen, Switzerland). Collision-induced dissociation (CID) spectra were acquired automatically in the data-dependent scan mode with the dynamic exclusion option. The CID raw spectra were extracted using Proteome Discoverer 1.4 (Thermo Fisher Scientific) and subjected to database searches using the Sequest algorithm. Peak list was compared with the Mouse International Protein Index version 3.84 database (European Bioinformatics Institute) including sequences of histone variants with the use of the Sequest algorithm. Additional details can be seen in Additional file 2: Supplemental Methods.

### GFP-fused histone H3.1 variant constructs and cell line selection

All cDNAs for histone H3 variants were purchased (Eurofins Genomics, Tokyo, Japan). The coding sequences are shown in Additional file 2: Figure S1. cDNAs were ligated into the Bidirectional Tet expression vector, pT2A-TRETIBI (modified Clontech Tet-On system), which contains TolII transposon elements and an EGFP cDNA located upstream of the cDNA sequence, and which was modified from pT2AL200R150G. pT2A-TRETIBI/EGFP-H3.1 transfection was performed using Lipofectamine 2000 (Life Technologies, Carlsbad, CA, USA). C2C12 cells at 20–30 % confluence were transfected with an expression vector (4 μg plasmid DNA per 100-mm plate), pCAGGS-TP encoding transposase (kindly provided by Dr. Kawakami, National Institute of Genetics, Japan), and pT2A-CAG-rtTA2S-M2 and incubated for 24 h. To create cell lines stably expressing each GFP-tagged histone variant, transfected cells were cultured for 14–21 days in the presence of 1 μg/ml doxycycline and 1 μg/ml G418. Finally, GFP-positive cells were selected using fluorescence activating cell sorting.

### Cells

C2C12 cells or stable clones were grown in Dulbecco’s modified Eagle’s medium (DMEM) supplemented with 20 % fetal bovine serum. Undifferentiated cells were harvested at 60–70 % confluence. Differentiated cells were transferred to DMEM containing 2 % horse serum upon reaching confluence and harvested 48 h later.

### Frap

Live-cell imaging was performed using a confocal microscope (FV-1000; Olympus) equipped with a heated stage (Tokai Hit) with a CO_2_-control system (Tokken) using a 60× PlanApoN Oil SC (NA = 1.4) objective lens. For FRAP, images were collected (256 × 256 pixels, zoom 8, scan speed 2 µs/pixel, pinhole 800 μm, BA505 emission filter, and 0.2 % transmission of an 488-nm Ar laser) without intervals, a 2-μm-diameter spot was bleached using 100 % transmission of a 488-nm laser, and a further 90 images were collected using the original setting. The fluorescence intensity of the bleached area was measured using Fiji (http://fiji.sc). For Fig. 3a, fluorescence images were collected under the following conditions: 512 × 512 pixels, zoom 8, scan speed 4 µs/pixel, pinhole 100 μm, 6 line averaging, BA505 emission filter, and 1 % transmission of a 488-nm Ar laser.

### Immunoblotting

Cells were washed twice with phosphate-buffered saline (PBS), centrifuged, and then resuspended in 2× SDS sample buffer containing prepared acid extract [44]. Samples were separated by SDS-PAGE and transferred to polyvinylidene fluoride membranes using the Trans-Blot Turbo Transfer System (Bio-Rad Laboratories, Hercules, CA, USA). Membranes were blocked for 1 h in 5 % (w/v) skimmed milk in Tris-buffered saline containing 0.05 % (v/v) Tween 20 (TBST), then incubated with primary antibodies in Hikari Solution A (Nacalai Tesque) followed by incubation with horseradish peroxidase-labeled secondary antibodies and detected using the Chemi-Lumi One Ultra (Nacalai Tesque). The primary antibodies used included rabbit anti-Hsp90 (H-114, Santa Cruz Biotechnology, 1:1000), anti-H2B (Imagenex, 1:1000), anti-H4 (Abcam, 1:1000), mouse anti-GFP (GF200, Nacalai Tesque, 1:500), rat anti-H3.3 (6C4A3, hybridoma supernatant, 1:1000), anti-H3.1/H3.2 (6G3C7, hybridoma supernatant, 1:1000) and anti-H3 (1G1, hybridoma supernatant, 1:1000). Secondary antibodies were horseradish peroxidase-conjugated anti-rabbit, anti-mouse and anti-rat IgG antibodies (GE Healthcare, 1:5000).

### Quantitative RT-PCR

Total RNA was isolated and reversed-transcribed using PrimeScript Reverse Transcriptase (Takara Bio) and an oligo dT primer, as previously described [4]. qPCR was performed using Thunderbird qPCR Mix (Toyobo). Primers used are listed in Additional file 2: Supplementary Information. qPCR data were normalized to *Gapdh* expression levels and presented as the mean ± standard deviation of three independent experiments.

### Chromatin immunoprecipitation

Cultured cells were cross-linked in 0.5 % formaldehyde and suspended in ChIP buffer (5 mM PIPES, 200 mM KCl, 1 mM CaCl2, 1.5 mM MgCl_2_, 5 % sucrose, 0.5 % NP-40, and protease inhibitor cocktail; Nacalai Tesque). Samples were sonicated for 5 s three times and digested with micrococcal nuclease (1 μl; New England Biolabs, Ipswich, MA, USA) at 37 °C for 40 min. The digested samples were centrifuged at 15,000×*g* for 10 min. Supernatant containing 4–8 μg DNA was incubated with a rat monoclonal antibody against GFP (1A5, 2 μg, Bio Academia) pre-bound to magnetic beads at 4 °C overnight with rotation. The immune complexes were eluted from the beads using 1 % SDS in TE, followed by washing with ChIP buffer and TE buffer (both twice). Cross-links were reversed, and DNA was purified using a Qiaquick PCR purification kit (Qiagen, Valencia, CA, USA).

### ChIP sequencing, read alignments and ChIP-Seq data analysis

ChIP sample preparations from GFP-tagged histone H3 variant-expressing cells were performed as described above. The ChIP library was prepared according to the Illumina protocol and sequenced on the Illumina HiSeq 1500 system. The sequence reads for GFP and Input were aligned to the reference mouse genome (mm9, build 37) using Bowtie 2 software (version 2.2.2) [45]. PCR duplicates were removed from uniquely mapped reads using samtools (version 0.1.19). To call peaks, we used MACS (version 2.0.10) and the parameters: *callpeak–gsize mm–nomodel–broad –extsize fragment-size–to-large–pvalue 1e-3* [29]. We defined “ChIP-Seq signal intensity” as described below. First, mapped reads on the genome in a defined window size (in the case of an IGV; Integrated Genome Browser screenshot: 10,000 bp windows by 1000 bp intervals or 1000 bp windows by 100 bp intervals; in other cases: 2 kb from the TSS) were counted and then normalized as RPKM (reads per kilobases per million reads) [46]. The ChIP-Seq signal intensities were then calculated as RPKM differences between ChIP and input DNA control data (i.e., ChIP–control) for each window.

### mRNA-Seq and mRNA-Seq data analysis

Total RNAs from growth and differentiated (i.e., post-differentiation) state C2C12 cells were obtained as previously described [4]. Library preparation was performed according to the protocol developed by Illumina. Sequenced reads of GFP-tagged H3 variant-overexpressed cells were mapped onto the mouse genome (mm9) using Tophat (version 2.0.8) [47]. Gene expression levels (FPKM; Fragments per kilobase of exon per million mapped sequence reads) were estimated using the cuffdiff program in Cufflinks (version 2.0.1) [47] using mapped reads and the software’s default parameters. Principal component (PC) analysis was performed against an FPKM matrix of gene expression profiles with rows of genes and columns of samples (H3 variant-expressing cells). The matrix was log_10_ transformed and the column scaled as mean = 0 and standard deviation = 1. The expression profiles (log_10_ transformed FPKM matrix) of wild-type (WT) cells at growth state (0 h) and differentiated state (48 h) were orthogonally projected onto the plane spanned by the 1st and 2nd PCs to compare with profiles of H3 variant-expressing cells.

### RT-PCR amplicon-Seq data analysis

The expression levels of PC contributing genes were evaluated by counting the amplicons of specific primers for each gene (amplicon sequencing) with three biological replicates. All sequenced reads were mapped on mouse transcript references converted from refFlat’s GTF file using the *gffread* command in Cufflink. The primer list is shown in Additional file 2: Supplementary Information.

### Data access

All deep-sequencing data in this study including ChIP-Seq, mRNA-Seq and 3′-seq were submitted to DDBJ Sequence Read Archive with the accession number [DDBJ:DRA002463]. The processed data including gene expression tables and ChIP-Seq track data (bigWig file used for IGV screen shot) are also accessible through GEO Series accession number [GEO:GSE63890].

## Additional files

**Additional file 1:** Predicted CDS of mouse H3 variants, contains Table S1, which lists the coding DNA sequence (CDS) locus information of the predicted mouse H3 variant genes in an Excel file.
**Additional file 2:** Supplementary Information, contains Supplemental Methods, Figures S1–S14 and Tables S5–S7 in a single PDF file.
**Additional file 3:** Predicted CDS of human histone H3/H4 variants, contains Table S2, which lists the CDS locus information of the predicted human histone H3 and H4 variants in an Excel file.
**Additional file 4:** Predicted CDS of rat histone H3 variants, contains Table S3 for the rat H3 sequence prediction results as an Excel file, which is the same format as Table S2.
**Additional file 5:** Similarity of amino acid and DNA sequence, contains Table S4, which shows amino acid and DNA sequence similarities between mouse H3 variant proteins/genes in Excel file.

## Abbreviations

GFP: green fluorescence protein
TSS: transcription start site
ChIP: chromatin immunoprecipitation
FRAP: fluorescence recovery after photobleaching
ORF: open reading frame
UTR: untranslated region
FPKM: fragments per kilobase per million fragments mapped
PC: principal component
