# Supplementary material for: Tissue-specific expression of histone H3 variants diversified after species separation
Source: Epigenetics Chromatin. 2015 Sep 17;8:35. doi: 10.1186/s13072-015-0027-3 (PMC4574566; doi:10.1186/s13072-015-0027-3)
Supplement: Supplementary file 2 — Additional file 2: Supplementary Information, contains Supplemental Methods, Figures S1–S14 and Tables S5–S7 in a single PDF file. [file 13072_2015_27_MOESM2_ESM.pdf]

Supplementary Information

Tissue-specific expression of histone H3 variants diversified after species separation

Kazumitsu Maehara[1,4], Akihito Harada[1,4], Yuko Sato[2], Masaki Matsumoto[3], Keiichi I. Nakayama[3], Hiroshi Kimura[2], Yasuyuki Ohkawa[1*]

[1]Department of Advanced Medical Initiatives, JST-CREST, Faculty of Medicine, Kyushu University, Fukuoka 812-8582, Japan. [2]Department of Biological Sciences, Graduate School of Bioscience and Biotechnology, Tokyo Institute of Technology, Yokohama, 226-0026, Japan. [3]Department of Molecular and Cellular Biology, Medical Institute of Bioregulation, Kyushu University, 3-1-1 Maidashi, Higashi-ku, Fukuoka, Fukuoka 812-8582, Japan.

[4] These authors contributed equally to this work.

*To whom correspondence should be addressed

E-mail: yohkawa@epigenetics.med.kyushu-u.ac.jp

Phone: +81-92-642-6216, Fax: +81-92-642-6099

Supplemental Methods

Phylogenic tree of histone H3 family

The 1,102 known histone H3 related protein sequences from all organisms (search term "family:histone H3") were taken from the UniProt database [1]. We used only reviewed protein sequences and newly determined sequences of mouse and human. The rat provisional genes were incorporated from TrEMBL database ("tr-" prefix). The phylogenic distance matrix was calculated using Clustal-Omega [2] and visualized with Dendroscope [3].

Gene expression analysis using ENCODE CSHL RNA-seq

The raw sequence data (FASTQ file) of ENCODE CSHL long RNA-seq data of mouse tissues were downloaded from: http://hgdownload.cse.ucsc.edu/goldenPath/hg19/encodeDCC/wgEncodeCshlLongRnaSeq/

We treated pair-end tags as separate sequence single-end tags to detect exactly matching genomic DNA sequences among the highly similar H3 gene family. The read count of overlapping uniquely mapped reads on the H3 gene defined by refFlat annotation were normalized as RPM. The RPMs of replicates and read-1/2 were averaged.

Proteasome inhibitor assay

The proteasome inhibitor assay was performed according to a previous report [4], except that treatment times were for 6 h. Cells were grown on 6-well plates and treated with proteasome inhibitor, 5 μM MG132 (Calbiochem, San Diego, CA, USA) or 5 μM lactacystin (Calbiochem) for 6 h. The cells were immunoblotting as described according to the immunoblot protocol in [4].

Mono-nucleosome analysis

Analysis of mono-nucleosomes was performed as described previously [5], except that 5 μl MNase (New England Biolabs) was added.

Cell fusion assay

Cell fusion assays were performed as previously described [6, 7]. C2C12 cells expressing histone-EGFP were fused with HeLa cells expressing mCherry-PCNA [8] using polyethylene glycol 1500 (Roche). After one hour, cells were fixed and observed using a confocal microscope (FV-1000; Olympus, Japan) with a 60X PlanApo SC (NA = 1.4) objective lens.

LC-MS/MS analysis

Peptides were dissolved in a solution containing 0.1% trifluoroacetic acid and 2% acetonitrile and analyzed by an LTQ Orbitrap Velos Pro mass spectrometer (Thermo Fisher Scientific, Waltham, MA) coupled with a nanoLC instrument (Advance, Michrom BioResources, Auburn, CA) and an HTC-PAL autosampler (CTC Analytics, Zwingen, Switzerland). Peptide separation was performed with an in-house pulled fused silica capillary (internal diameter, 0.1 mm; length, 15 cm; tip internal diameter, 0.05 mm) packed with a 3 μm C_{18} L-column (Chemicals Evaluation and Research Institute, Japan). The mobile phases consisted of 0.1% formic acid (A) and 100% acetonitrile (B). Peptides were eluted with a gradient of 5–35% acetonitrile for 20 min at a flow rate of 300 nL/min. Collision-induced dissociation (CID) spectra were acquired automatically in the data-dependent scan mode with the dynamic exclusion option. Full MS spectra were obtained with Orbitrap in the mass/charge (m/z) range of 300–2000 with a resolution of 30,000 at m/z 400. The six most intense precursor ions (minimum ion count threshold of 1,000) in the full MS spectra were selected for subsequent ion-trap MS/MS analysis with the automated gain control (AGC) mode. The AGC was set to 1.00×10^1 for full MS and 1.00×10^4 for CID MS/MS. The

normalized collision energy values were set to 35%. Lock mass function was activated to minimize mass error during analysis. The CID raw spectra were extracted using Proteome Discoverer 1.4 (Thermo Fisher Scientific) and subjected to database searches using the Sequest algorithm. Peak list was compared with the Mouse International Protein Index version 3.84 database (European Bioinformatics Institute) including sequences of histone variants with the use of the Sequest algorithm. Trypsin was selected as the enzyme used, the allowed number of missed cleavages was set at 2, and carbamidomethylation of cysteine was selected as the fixed modification. Oxidized methionine, phosphorylations on serine/threonine/tyrosine and protein NH_2-acetylation were searched as variable modifications. Precursor mass tolerance was 10 ppm, and tolerance of MS/MS ions was 0.8 Da.

Primers list for quantitative RT-PCR

Gapdh:

5'-GACTTCAACAGCAACTCCCACTCT-3'

5'-GGTTTCTTACTCCTTGGAGGCCAT-3'

NeoR:

5'-ATCAGCCATGATGGATACTTTCTC-3'

5'-GTTCTTTTTGTCAAGACCGACCT-3'

EGFP:

5'-AAGCTGACCCTGAAGTTCATCTGC-3'

5'-TAGTTGCCGTCGTCCTTGAAGAAG-3'

Primer list for amplicon sequence

		Forward		Reverse	
	**gene symbol**	**locus (from TSS)**	**sequence**	**locus (from TSS)**	**sequence**
**PC1+**	*Actc1*	1136-1159	ACCTTCCAGCAAATGTGGATCAGC	1283-1306	AATGACTGATGAGAGATGGGGAGG
	Tnnc2	387-410	CGCAGACGGCTACATTGATGCTGA	558-581	CTGGATGGACACGAACAAAGACCG
	Tnni1	820-843	AGCCCCACTCACCTATCTTTCTGC	918-941	CATCTCTCTTCCCCTTTGTGTGCC
	Tnni2	463-486	AAGACACTGAGAAGGAACGGGACC	638-661	TGAATCCTTTATTGGAGCGAGGCC
	Tnnt3	812-835	GCTGGAAGTAAAAGAGCAGAGAGG	961-984	ACTTTATTCCTAGACCCCAGAAGG
**PC1-**	*Aurka*	20-40	CTGGATGCTGCAAACGGATAG	124-102	CGAAGGGAACAGTGGTCTTAACA
	Cdc20	10-30	TTCGTGTTCGAGAGCGATTTG	200-179	ACCTTGGAACTAGATTTGCCAG
	Mcm5	94-114	CAGAGGCGATTCAAGGAGTTC	211-191	CGATCCAGTATTCACCCAGGT
	Pttg1	45-63	CCGCCGTTTGGCATCTAAG	217-197	TCATAGGCTTTTCGGCAACTC
	Top2a	112-134	CAACTGGAACATATACTGCTCCG	296-274	GGGTCCCTTTGTTTGTTATCAGC
**PC2+**	*Acox2*	7-25	AACCCAGGGGATCGAGTGT	149-130	CGCAGCTCAGTGTTTGGGAT
	Akr1b7	128-150	TTGACTGTGCCTATGTGTATCAC	288-268	GGTGTTGTCGAAGGCTTTCTT
	Avil	93-113	GAGTGCTCACGGCAACTTCTA	205-187	GGGAGGAGTCCTTCCCGAT
	Cdsn	58-78	TTGCTGATGGCCGGTCTTATT	183-161	GCCAGTCTTTCCAATGAGACAAG
	H2-M10.1	53-73	ACCTGAACCACTATTGTGCAG	201-181	TGCAGATGGGTCTTTGCTGTC
**PC2-**	*Angpt1*	46-65	CACATAGGGTGCAGCAACCA	154-134	CGTCGTGTTCTGGAAGAATGA
	Dag1	130-148	CTTGAGGCGTCCATGCACT	258-236	GGCAATTAAATCCGTTGGAATGC
	Gamt	93-111	CACGCACCTGCAAATCCTG	211-191	TACCGAAGCCCACTTCCAAGA
	Mgp	45-64	GGCAACCCTGTGCTACGAAT	178-156	CCTGGACTCTCTTTTGGGCTTTA
	Synpo	2425-2443	CCTGCCCGTAACTTCCGTG	2555-2537	GAGCGGCGGTAGGGAAAAG

Supplemental Figure Legends

Figure S1 | Alignment of mouse histone H3 variant coding sequences

Clustal-Omega was used to align all mouse histone H3 variant coding sequences.

Figure S2 | Phylogenic tree of the histone H3 family and the novel H3 variants

(A) The phylogenic tree of the histone H3 protein family is shown. UniProt IDs are used to label the proteins. An enlarged view of the H3.3 **(B)** or H3.1 **(C)** cluster is shown in a separate panel. The red labels are the novel mouse and human H3-related sequences determined in this paper; blue indicates known H3 proteins of mouse and human; and purple indicates rat pseudogenes found in the TrEMBL database.

Figure S3 | Protein sequence of H3t is similar to that of human H3T and novel histone H2A gene (*H2A.J*-like and *H2A.Z* like)

(A) Clustal-Omega was used to align H3t, human H3T and H3.1. Mouse H3t and human H3T share two characteristic amino-acids compared with H3.1, which are A26V and A100S.

(B) Clustal-Omega was used to align the H2A genes, *H2A.Z*, *H2A.J* (known genes) and *H2A.Z*-like and *H2A.J*-like (novel genes). *H2A.Z*-like gene does not contain the full core domain sequence. Accordingly, the *H2A.Z*-like gene was dropped from the identification list.

Figures S4 | Limited evaluation of the expression of histone H3 genes using RNA-seq

The gene expression levels of histone H3 genes derived from ENCODE CSHL long RNA-seq data are shown as a heatmap. The columns represent tissues and the rows represent H3 variants. The rainbow colors indicate expression level (log2RPM) as indicated in the color key (blue: low, red: high). The order of rows/columns was determined by hierarchical clustering of expression levels.

Figure S5 | Genomic and Epigenomic feature around the novel H3 variants

IGV screenshots of *H3f3a* (A) and *H3f3b* (B) 14 novel H3 variant genes (C-P). The lanes above Refseq gene shows the ENCODE ChIP-Seq signals of H3ac, H3K4me3, H3K27me3 and the ChIP-Seq Input in C2C12 cells provided by Caltech. The lane named "Prediction" shows the predicted gene structure of novel H3 variants by *in silico* hyblidyzation. The mappability lane shows the unique mappabilities on mouse genome for 50 mer sequence. The rest of data shows CpG Island and Repbase for repetitive DNA elements were shown. The ENCODE ChIP-Seq, CpG islands and Repbase data were loaded by IGV software's utility. The CRG mappability score were downloaded from below:

http://hgdownload.cse.ucsc.edu/goldenPath/mm9/encodeDCC/wgEncodeMapability

Figure S6 | Alignment of mouse histone H3 variant's 3'-UTR sequences

Clustal-Omega was used to align sequences. The reverse complement sequences were shown to start from 3'-end sequence of 3'-UTR.

Figure S7 | Relative expression levels of novel histone H3 genes

The relative gene expression levels of all 14 variants compared to replication-independent histone genes *H3f3a* and *H3f3b* are shown. The y-axis indicates the ratio of expression level of each gene compared to the sum of 3'-seq tags of H3.3 coding genes (*H3f3a* + *H3f3a*).

Figure S8 | Identification of endogenous H3t and H3mm7

(A) Coomassie Brilliant Blue (CBB) stain of acid extracted fractions of mouse tissue. Red line indicates fractions that were analyzed by mass spectrometry for the detection of novel histone H3 variants.

(B–D) MS/MS spectra assigned for novel H3 variants (H3t and H3mm7). MS/MS spectra corresponding to ions m/z 507.82306 (B), 338.52264 (C) and 472.28198 (D) are represented. The right tables show the observed product ions matched with theoretical ones (y-ions: red and b-ions: blue).

Figure S9 | Stable incorporation of GFP-fused histone variants in mitotic cells

Confocal images of GFP-H3 variants in mitotic cells. H3.1, H3.2, H3.3, H3t, H3mm7, H3mm11, H3mm12, H3mm13 and H3mm16 were localized to mitotic chromosomes. H3mm15 and H3mm18 were partly localized to mitotic chromosomes. H3mm6, H3mm8, H3mm9, H3mm10, H3mm14 and H3mm17 appeared to be diffused freely. Scale bars, 10 μm

Figure S10 | Histone variants incorporated into chromatin form nucleosomes.

Mononucleosomes were prepared from C2C12 myoblast cells (WT) and those expressing GFP-tagged histone H3 variants, using MNase digestion. After binding to HAP resin and extensive washing with 0.5 M NaCl, nucleosomes were eluted using 0.5 M sodium phosphate. **(A)** Nucleosome size was determined by 1.5% agarose gel electrophoresis. The size of the DNA was slightly shorter than the typical mononucleosome (~146 bp), which was probably caused by an extensive MNase digestion to avoid leaving oligonucleosomes. **(B)** GFP-H3 variant-containing mononucleosomes were precipitated using a GFP-specific antibody. All GFP-H3 variants analyzed (i.e., H3.1, H3.3, H3t, H3mm7 and H3mm14) were recovered from mononucleosome samples with other core histones. As the expression of GFP-H3mm7 was lower than the other variants, GFP-H3mm7 was present at a lower concentration in the input mononucleosome sample. To highlight GFP-H3mm7 immunoprecipitates, the WT, H3mm7 and H3mm14 samples were run in a separate gel and subjected to longer exposure.

Figure S11 | Cell fusion assay

C2C12 cells expressing histone-EGFP were fused with recipient HeLa cells expressing mCherry-PCNA. In heterokaryons, H3.1-EGFP and H3t-EGFP, but not H3.3-EGFP and H3mm7-EGFP, were concentrated in mCherry-PCNA-positive replication foci in recipient HeLa nuclei (open arrowheads). Histone-EGFP donor C2C12 nuclei are indicated by closed arrowheads. Scale bar, 10 μm.

Figure S12 | The expression of non-incorporated GFP-tagged histone H3 variants is unstable

(A) Protein levels of GFP-tagged histone H3 variants based on immunoblots of whole cell lysates of histone H3 variant-expressing C2C12 cells in the undifferentiated state. Immunoblots using anti-GFP antibody could not detect H3mm6, H3mm8m H3mm9, H3mm10, H3mm14 or H3mm17. Hsp90 was used as a loading control.

(B) Transcript levels of GFP-tagged histone H3 variants as detected by quantitative RT-PCR. The mRNA level of GFP and neomycin resistance genes (*neoR*) showed that H3mm14 and H3mm18 mRNA levels were high; however, both had low protein levels, as shown in A. mRNA levels were normalized to the expression level of *Gapdh*. The mRNA level of *Eef1a1* was used as a control for the amount of RNA used.

Figure S13 | The proteasome is involved in the degradation of histone H3 variants that are unincorporated into chromatin

C2C12 cells (Wild-type, GFP-H3.1, GFP-H3.3, GFP-H3t, GFP-H3mm7, GFP-H3mm8, GFP-H3mm14 and GFP-H3mm18) were pre-treated with 5 μm MG132 for 6 h. Whole cell lysates were analyzed by western blotting using GFP and H3-specific antibodies. The H3-specific antibody was a protein loading control. Unincorporated histone H3 variants

(H3mm8, H3mm14 and H3mm18) increased when the proteasome inhibitor was added to the C2C12 cells expressing them.

Figure S14 | H3 variant incorporation map of the top 40 contributing genes for PC1+/− and PC2+/−

(A) The columns of the matrix are H3 variant-expressing cells and rows are genes, which are ordered by hierarchical clustering using the measure, log2 fold-change (FC); log2(RPM_{ChIP}/RPM_{Input}) calculated within 2 kb of the TSS. Colors indicate the intensities of the FCs as scale-bar located on the left.

(B) IGV screen shot of ChIP-Seq data near the PC2+ contributing gene, *Cdsn*. The distribution of H3 variants around *Cdsn* showed specific expression of the D3 group. The signals colored in light and dark orange are the H3 variants of D3 group in the growth and differentiated state, respectively. ChIP-Seq signal intensities are indicated [min-max] to the right of each lane.

Supplemental Tables

Table S1 | Predicted CDS of mouse H3 variants

(Additional file 2 in Excel format)

Table S2 | Predicted CDS of human histone H3/H4 variants

(Additional file 3 in Excel format)

Table S3 | Predicted CDS of rat histone H3 variants

(Additional file 4 in Excel format)

Table S4 | Similarity of amino acid and DNA sequence

(Additional file 5 in Excel format)

Table S5-S7

(in next page)

Table S5 | Predicted histone H2A and H2B variant

Gene symbol	Locus (Strand)	Product	Type	Status
-	chr5:143918809-143919139 (-)	H2A.J like[a]	unknown	full-frame
(Gm8203)	chr6:116123754-116124141 (+)	H2A.Z like[a]	unknown	full-frame
-	chr9:17902398-17902725 (+)	H2A.J like	unknown	Nonsense
(Gm17284)	chr12:84961630-84961834 (-)	H2A.Z like	predicted	missense
(Gm11336)	chr13:23837988-23838276 (-)	H2A.3 like	predicted	missense
(Gm12957)	chr4:119193595-119193971 (+)	H2B1F like	predicted	missense
(Gm6574)	chr7:111677552-111677585 (-)	-	pseudo	missense
(Hist3h2bb-ps)	chr11:58767549-58767930 (-)	H2B3B	pseudo	full-frame

[a] Used sequences in Figure S3B.

Table S6 | 3’-seq-quantified individual gene expression levels of novel H3 variants

H3 variant	Brain	Liver	SKMuscle	Testis	C2C12-Growth state	C2C12-Differentiated state
H3f3a	1.55	0.00	0.13	1.09	7.41	2.53
H3f3b	0.00	0.00	0.13	2.18	0.00	3.80
H3mm6	0.00	0.00	0.00	0.14	0.00	0.00
H3mm7	5.68	1.58	7.80	39.48	9.26	9.36
H3mm8	1.72	1.11	2.08	8.98	7.41	2.28
H3mm9	0.00	0.00	0.00	0.14	0.00	0.00
H3mm10	0.00	0.00	0.00	0.00	0.00	0.00
H3mm11	0.00	0.95	0.00	0.00	0.00	0.00
H3mm12	0.00	0.00	0.00	0.00	0.00	0.00
H3mm13	12.73	3.48	21.85	15.11	1.85	6.33
H3mm14	0.00	0.63	0.13	0.27	0.00	0.00
H3mm15	35.43	9.81	69.96	55.41	37.04	43.52
H3mm16	0.00	0.00	0.00	0.14	0.00	0.00
H3mm17	0.00	0.00	0.00	0.00	0.00	0.00
H3mm18	0.00	0.00	0.00	0.00	0.00	0.00
H3t	0.00	0.00	0.00	0.41	0.00	0.00

Table S7 | Correlation coefficients between gene expression profile pairs

		H3.1 (G)	H3t (G)	H3.2 (G)	H3.3 (G)	H3mm7 (G)	H3mm11 (G)	H3mm12 (G)	H3mm13 (G)	H3mm16 (G)	H3.1 (D)	H3t (D)	H3.2 (D)	H3.3 (D)	H3mm7 (D)	H3mm11 (D)	H3mm12 (D)	H3mm13 (D)
**Growth (G)**	**H3t**	0.90																
	**H3.2**	0.96	0.92															
	**H3.3**	0.99	0.90	0.95														
	**H3mm7**	0.96	0.91	0.92	0.97													
	**H3mm11**	0.95	0.93	0.96	0.95	0.95												
	**H3mm12**	0.95	0.92	0.97	0.94	0.93	0.98											
	**H3mm13**	0.92	0.88	0.97	0.92	0.90	0.96	0.97										
	**H3mm16**	0.94	0.90	0.96	0.94	0.93	0.97	0.98	0.98									
**Differentiated (D)**	**H3.1**	0.91	0.85	0.90	0.90	0.87	0.90	0.91	0.87	0.87								
	**H3t**	0.85	0.87	0.90	0.84	0.81	0.89	0.91	0.88	0.88	0.95							
	**H3.2**	0.80	0.81	0.84	0.79	0.76	0.81	0.83	0.81	0.80	0.91	0.91						
	**H3.3**	0.89	0.80	0.87	0.90	0.85	0.85	0.85	0.83	0.82	0.92	0.87	0.89					
	**H3mm7**	0.87	0.83	0.85	0.88	0.89	0.84	0.83	0.80	0.80	0.87	0.81	0.82	0.95				
	**H3mm11**	0.86	0.80	0.88	0.87	0.82	0.85	0.85	0.85	0.84	0.90	0.89	0.89	0.96	0.91			
	**H3mm12**	0.76	0.72	0.80	0.76	0.72	0.78	0.80	0.80	0.77	0.89	0.88	0.93	0.85	0.79	0.87		
	**H3mm13**	0.80	0.76	0.83	0.80	0.77	0.82	0.83	0.83	0.82	0.90	0.90	0.94	0.89	0.82	0.91	0.98	
	**H3mm16**	0.80	0.76	0.83	0.80	0.77	0.82	0.83	0.83	0.82	0.90	0.90	0.92	0.88	0.81	0.91	0.98	0.98

Supplemental Tables Legends

Table S1 | Predicted CDS of mouse H3 variants

H3 variant screening results from mouse genome (mm9). Prediction IDs come from our screening method and are written sequentially in genomic coordinate. The list contains predicted CDS coding > 30 a.a.. Gene names (in parentheses) are the known provisional or computationally predicted gene names.

Table S2 | Predicted CDS of human histone H3/H4 variants

H3 and H4 variant screening results from human genome (hg19). Gene names assigned in the column "Ensemble gene name" are based on overlapping genes annotated by Ensembl project. The columns "Blast gi" and "Description" are based on BLAST (Basic Local Alignment Search Tool) search results using the protein sequence shown in "Predicted product".

Table S3 | Predicted CDSs of rat histone H3 variants

H3 variant screening results from rat genome (rn5). The table shows predicted locus, protein product and of rat H3 variants. The gene name is given by overlapping gene definition of Ensemble genes (Rnor 5.0.73).

Table S4 | Similarity of amino acid and DNA sequence

Sequence similarities between mouse H3 variants. Data below the diagonal show DNA sequence similarities; above show amino acid sequence similarities. Two similarity measures in each cell are separated by the vertical dotted lines: left is the edit distance (Levenshtein distance) with the unit of a.a./base; right is the percent identity.

Table S5 | Predicted histone H2A and H2B variants

Screening results of mouse histone H2A/B. The column "Gene symbol" shows the known provisional or computationally predicted gene names (pseudo or predicted in Type column).

Table S6 | 3'-seq-quantified individual gene expression levels of novel H3 variants

Gene expression levels of histone H3 variants quantified by 3'-seq analysis. Numbers indicate expression levels calculated as RPM normalized read counts mapped in the 3'-UTR region.

Table S7 | Correlation coefficients between gene expression profile pairs

Pearson's correlation coefficients of gene expression profiles of forced H3 variant expression cells.

Supplemental References

1. Bateman A, Martin MJ, O'Donovan C, Magrane M, Apweiler R, Alpi E et al. UniProt: a hub for protein information. Nucleic Acids Research. 2015;43(D1):D204-D12. doi:DOI 10.1093/nar/gku989.
2. Sievers F, Wilm A, Dineen D, Gibson TJ, Karplus K, Li WZ et al. Fast, scalable generation of high-quality protein multiple sequence alignments using Clustal Omega. Molecular Systems Biology. 2011;7. doi:ARTN 539
DOI 10.1038/msb.2011.75.
3. Huson DH, Richter DC, Rausch C, Dezulian T, Franz M, Rupp R. Dendroscope: An interactive viewer for large phylogenetic trees. Bmc Bioinformatics. 2007;8. doi:Artn 460
Doi 10.1186/1471-2105-8-460.
4. Lee JO, Lee SK, Kim N, Kim JH, You GY, Moon JW et al. E3 ubiquitin ligase, WWP1, interacts with AMPKalpha2 and down-regulates its expression in skeletal muscle C2C12 cells. J Biol Chem. 2013;288(7):4673-80. doi:10.1074/jbc.M112.406009.
5. Harada A, Maehara K, Sato Y, Konno D, Tachibana T, Kimura H et al. Incorporation of histone H3.1 suppresses the lineage potential of skeletal muscle. Nucleic Acids Res. 2015;43(2):775-86. doi:10.1093/nar/gku1346.
6. Schmidtzachmann MS, Dargemont C, Kuhn LC, Nigg EA. Nuclear Export of Proteins - the Role of Nuclear Retention. Cell. 1993;74(3):493-504. doi:Doi 10.1016/0092-8674(93)80051-F.
7. Kimura H, Cook PR. Kinetics of core histones in living human cells: Little exchange of H3 and H4 and some rapid exchange of H2B. J Cell Biol. 2001;153(7):1341-53. doi:DOI 10.1083/jcb.153.7.1341.
8. Sato K, Ishiai M, Toda K, Furukoshi S, Osakabe A, Tachiwana H et al. Histone chaperone activity of Fanconi anemia proteins, FANCD2 and FANCI, is required for DNA crosslink repair. Embo J. 2012;31(17):3524-36. doi:DOI 10.1038/emboj.2012.197.

Figure S1

```
Hist1h3a   ATGGCTCGTACTAAGCAGACCGCTCGCAAGTCCACCGGCGGCAAGGCCCCGCGCAAG 57
Hist2h3b   ATGGCTCGTACTAAGCAGACCGCTCGCAAGTCCACCGGCGGCAAGGCCCCGCGCAAG 57
H3 t       ATGGCACGCACCAAGCAGACGGCACGGAAGTCGACGGGAGGCAAGGCTCCGCGCAAG 57
H3mm8      ATGGCCCGAACCAAGCAGACCGCTAGGAAGTCCACCGGTGGGAAAGCCCCCCCCAAA 57
H3f3b      ATGGCCCGAACCAAGCAGACCGCTAGGAAGTCCACCGGTGGGAAAGCCCCCCGCAAA 57
H3f3a      ATGGCTCGTACAAAGCAGACTGCCCGCAAATCCACCGGTGGTAAAGCACCCAGGAAA 57
H3mm11     ATGGCTCTTACAAAGCAGACTGCCCGCAAATCCACCGGTGGTAAAGCACCCAGGAAA 57
H3mm15     ATGGCTCGTACAAAGCAGACTGCCCGCAAATCCACCGGTGGTAAAGCACCCAGGAAA 57
H3mm16     ATGGCTCGTACAAAGCAGACTGCCCACAAATCCACCGGTGGTAAAGCACCCAGGAAA 57
H3mm13     ATGGCTCGTACAAAGCAGACTGCCCGCAAATCCACCGGTGGTAAAGCACCCAGGAAA 57
H3mm7      ATGGCTCGTACAAAGCAGACTGCCCGCAAATCCACCGGTGGTAAAGCACCCAGGAAA 57
H3mm12     ATGGCTCGTACAAAGCAGACTGCCCGCAAATCCACGGGTGGTAACTCACCCAGGAAA 57
H3mm6      ATGGCTCGTACAAAGCAGACTGCCTGCAAATCCACCGGTGGTAAAGCACCCAGGAAA 57
H3mm18     ATGGCTCGTACGAAGCAGACTGCCCGCAAATCCACCGGTGATAAAGCACCCAGGAAA 57
H3mm17     ATGGCTCGTACAAAGCAGACTGCCCACAAATCCACCGGAAATAAAGCACCCAGGAAA 57
H3mm10     ATGGCTTGTACAAAGCAGACTGGCCACAAATCCACCGGTGGTAAAGCACCCAGGAAA 57
H3mm14     ATGGCTCCTACAAAGCAGACTGCCCGCAAATCCACCTGTGGTAAAGCACCCAGGAAA 57
H3mm9      ATGGCTCTTACAAAGCAGACTGCACACAAATCCAACGGTGGTAAAGCATCCAGGAAA 57

Consensus
           ATGGCTCGTACAAAGCAGACTGCCCGCAAATCCACCGGTGGTAAAGCACCCAGGAAA
```

```
Hist1h3a   CAGCTAGCCACCAAAGCCGCCCGCAAGAGCGCCCCGGCCACCGGCGGCGTGAAGAAG 114
Hist2h3b   CAGCTGGCCACCAAGGCCGCCCGCAAGAGCGCCCCGGCCACCGGCGGCGTGAAGAAG 114
H3 t       CAGCTAGCCACGAAGGTGGCCCGCAAGAGCGCCCCGGCCACCGGGGGCGTGAAGAAG 114
H3mm8      CAGCTGGCCACTAAGGCGGCTCGGAAAAGCGCGCCCTCTACCGGCAGGGTGAAGAAG 114
H3f3b      CAGCTGGCCACCAAGGCGGCTCGGAAAAGCGCGCCCTCTACCGGCGGGGTGAAGAAG 114
H3f3a      CAACTGGCTACAAAAGCCGCTCGCAAGAGTGCGCCCTCTACTGGAGGGGTGAAGAAA 114
H3mm11     CAACTGGCTACAAAAGCCACTCGCAAGAGTGCGCCCTCTACTGGAGGGGTGAAGAAA 114
H3mm15     CAACTGGCTACAAAAGCCGCTCGCAAGAGTGCGCCCTCTACTGGAGGGGTGAAGAAA 114
H3mm16     AAACTGGCTACAAAAGCCGCTCGCAAGAGTGCGCCCTCTACTGGAGGGGTGAAGAAA 114
H3mm13     CAACTGGCTACAAAAGCCGCTCGCAAGAGTGTGCCCTCTACTGGAGGGGTGAAGAAA 114
H3mm7      CAACTGGCTACAAAAGCCGCTCGCAAGAGTGCGCCCTCTATTGGAGGGGTGAAGAAA 114
H3mm12     CAACTGGCTACAAAAGCCGCTCGCAAGAGTGCGCCCTCTACTGGAGGGGTGAAGAAA 114
H3mm6      CAACTGGCTACAAAAGCCGCTCGCAAGAGTGCGCCCACTACTGGAGGGGTGAAGAAA 114
H3mm18     CAACTGGCTACAAAAGCCGCTCGCAAGAGTGCGCCCTCTACTGGAGGGGTGAAGAAA 114
H3mm17     CAACTGGCTACAAAAGCTGCTTGCAAGAGTGCGCCCTCTACTGGAGGGGTGAAGAAA 114
H3mm10     CAACGGGCTACAAAAGCCGCTCGCAAGAGTGCGCCCTCTACTGGATGGGTGAAGAAA 114
H3mm14     AAACTGGCTACAAAAGCCGCTCACAAGAGTGCGCCCTCTACTGGAGGAGTGAAGAAA 114
H3mm9      GAACTGCCTACAAAAGCCGCTCGCAAAAGGGCATCCTCTACTGGAGGGGTGAAGAAA 114

Consensus
           CAACTGGCTACAAAAGCCGCTCGCAAGAGTGCGCCCTCTACTGGAGGGGTGAAGAAA
```

```
Hist1h3a   CCTCACCGCTACCGTCCCGGCACCGTGGCGCTGCGCGAGATCCGGCGCTACCAGAAG 171
Hist2h3b   CCTCACCGCTACCGTCCCGGCACCGTGGCGCTGCGCGAGATCCGGCGCTACCAGAAG 171
H3 t       CCGCACCGCTACCACCCTGGCACGGTGGCGCTGCGCGAGATCCGGCGCTACCAGAAG 171
H3mm8      CCTCACCGCTACAGGCCAGGGACCGTGGCTCTGAGAGAGATCCTTCGTTACCAGAAA 171
H3f3b      CCTCACCGCTACAGGCCAGGGACCGTGGCTCTGAGAGAGATCCGTCGTTACCAGAAA 171
H3f3a      CCTCATCGTTACAGGCCTGGTACTGTGGCCCTCCGTGAAATCAGACGCTATCAGAAG 171
H3mm11     CCTCATCGTTACAGGCCTGGCACTGTGGCACTCCGTGAAATCAGACGCTATCAGAAG 171
H3mm15     CCTCATCGTTACAGGCCTGGTACTGTGGCACTCCGTGAAATCAGACGCTATCAGAAG 171
H3mm16     CCTCATCGTTACAGGCCTGGTACTGTGGCACTCCGTGAAATCAGACGCTATCAGAAG 171
H3mm13     CCTCATCGTTACAGGCCTGGTACTGTGGCACTCCGTGAAATCAGACGCTATCAGAAG 171
H3mm7      CCTCATCGTTACAGGCCTGGTACTGTGGCCCTCCGTGAAATCAGACGCTATCAGAAG 171
H3mm12     CCTCATCGTTACAGGCCTGGTACTGTGGCACTCCGTGAAATCAGACGCTATCAGAAG 171
H3mm6      CCTCATCTTTACAGGCCTGGTACTGTGGCACTCCATGAAATCAGACGCTATCAGAAA 171
H3mm18     CCTCATTGTTACAGGCCTGGTACTGTGGCACTCCGTGAAATCAGAAGCTATCAGAAG 171
H3mm17     CCTCATCGTTACAGGCCTGGTTCTGTGGCACACCCTGAAATCAGATGCTATCAGAAG 171
H3mm10     CCTCATCGTTACAGGCCTGGTACTGTGGCACTCAGTGAAATCAGACACTATCAGAAG 171
H3mm14     CCTCATCGATACAGGCCTGGTACTGTGGCACTCTGTGAAATCAGACGCTATCAGAAG 171
H3mm9      CCTCATCGTTACGGGCCTGGTACTGTAGCACTCCATGAAATCAGAAGCTATCAGAAG 171

Consensus
           CCTCATCGTTACAGGCCTGGTACTGTGGCACTCCGTGAAATCAGACGCTATCAGAAG
```

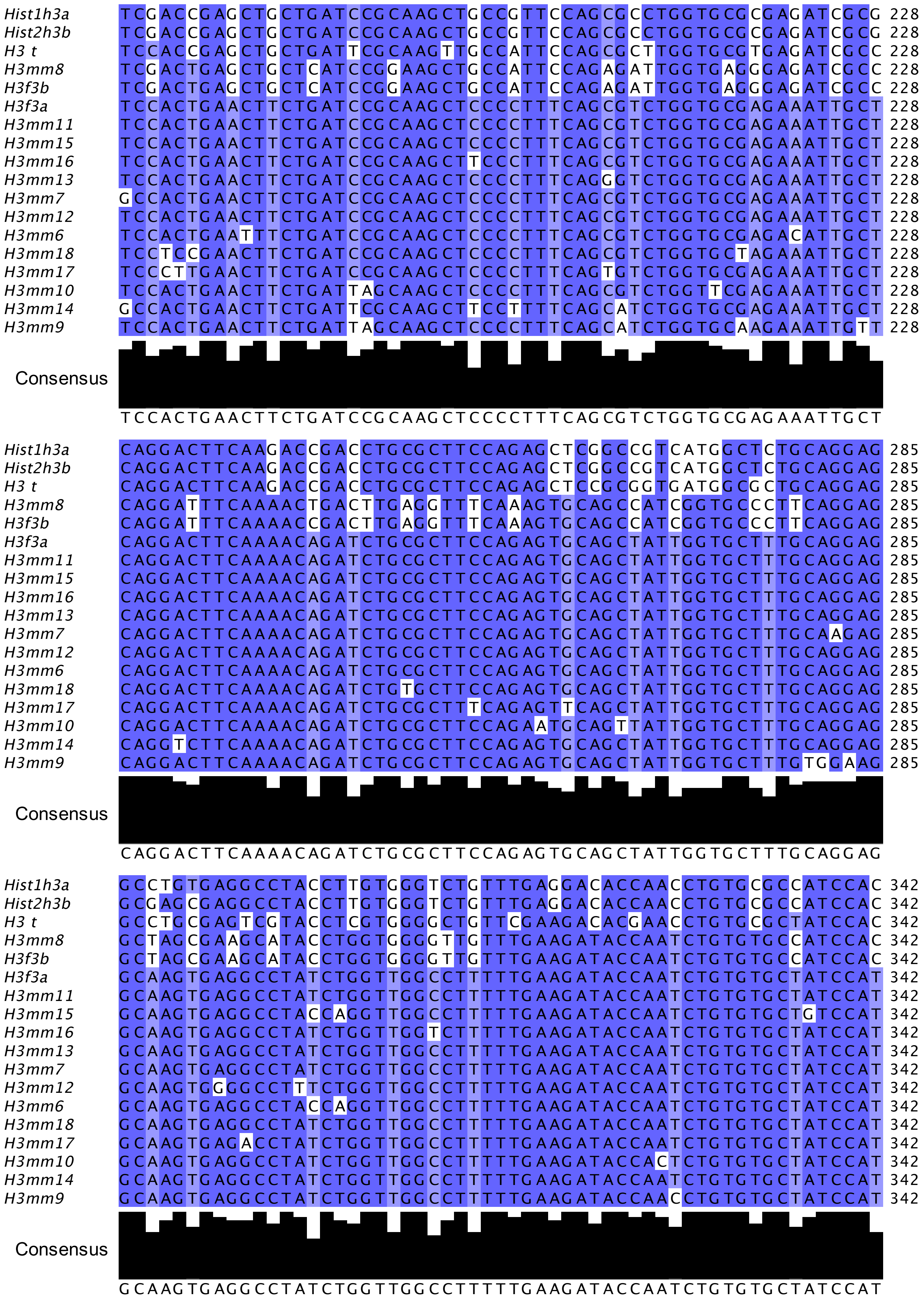

Hist1h3a TCGACCGAGCTGCTGATCCGCAAGCTGCCGTTCCAGCGCCTGGTGCGCGAGATCGCG 228
Hist2h3b TCGACCGAGCTGCTGATCCGCAAGCTGCCGTTCCAGCGCCTGGTGCGCGAGATCGCG 228
H3 t TCCACCGAGCTGCTGATTCGCAAGTTGCCATTCCAGCGCTTGGTGCGTGAGATCGCC 228
H3mm8 TCGACTGAGCTGCTCATCCGGAAGCTGCCATTCCAGAGATTGGTGAGGGAGATCGCC 228
H3f3b TCGACTGAGCTGCTCATCCGGAAGCTGCCATTCCAGAGATTGGTGAGGGAGATCGCC 228
H3f3a TCCACTGAACTTCTGATCCGCAAGCTCCCCTTTCAGCGTCTGGTGCGAGAAATTGCT 228
H3mm11 TCCACTGAACTTCTGATCCGCAAGCTCCCCTTTCAGCGTCTGGTGCGAGAAATTGCT 228
H3mm15 TCCACTGAACTTCTGATCCGCAAGCTCCCCTTTCAGCGTCTGGTGCGAGAAATTGCT 228
H3mm16 TCCACTGAACTTCTGATCCGCAAGCTTCCCTTTCAGCGTCTGGTGCGAGAAATTGCT 228
H3mm13 TCCACTGAACTTCTGATCCGCAAGCTCCCCTTTCAGGGTCTGGTGCGAGAAATTGCT 228
H3mm7 GCCACTGAACTTCTGATCCGCAAGCTCCCCTTTCAGCGTCTGGTGCGAGAAATTGCT 228
H3mm12 TCCACTGAACTTCTGATCCGCAAGCTCCCCTTTCAGCGTCTGGTGCGAGAAATTGCT 228
H3mm6 TCCACTGAATTTCTGATCCGCAAGCTCCCCTTTCAGCGTCTGGTGCGAGACATTGCT 228
H3mm18 TCCTCCGAACTTCTGATCCGCAAGCTCCCCTTTCAGCGTCTGGTGCTAGAAATTGCT 228
H3mm17 TCCCTTGAACTTCTGATCCGCAAGCTCCCCTTTCAGTGTCTGGTGCGAGAAATTGCT 228
H3mm10 TCCACTGAACTTCTGATTAGCAAGCTCCCCTTTCAGCGTCTGGTTCGAGAAATTGCT 228
H3mm14 GCCACTGAACTTCTGATTCGCAAGCTTCCTTTTCAGCATCTGGTGCGAGAAATTGCT 228
H3mm9 TCCACTGAACTTCTGATTAGCAAGCTCCCCTTTCAGCATCTGGTGCAAGAAATTGTT 228
Consensus
TCCACTGAACTTCTGATCCGCAAGCTCCCCTTTCAGCGTCTGGTGCGAGAAATTGCT
Hist1h3a CAGGACTTCAAGACCGACCTGCGCTTCCAGAGCTCGGCCGTCATGGCTCTGCAGGAG 285
Hist2h3b CAGGACTTCAAGACCGACCTGCGCTTCCAGAGCTCGGCCGTCATGGCTCTGCAGGAG 285
H3 t CAGGACTTCAAGACCGACCTGCGCTTCCAGAGCTCCGCGGTGATGGCGCTGCAGGAG 285
H3mm8 CAGGATTTCAAAACTGACTTGAGGTTTCAAAGTGCAGCCATCGGTGCCCTTCAGGAG 285
H3f3b CAGGATTTCAAAACCGACTTGAGGTTTCAAAGTGCAGCCATCGGTGCCCTTCAGGAG 285
H3f3a CAGGACTTCAAAACAGATCTGCGCTTCCAGAGTGCAGCTATTGGTGCTTTGCAGGAG 285
H3mm11 CAGGACTTCAAAACAGATCTGCGCTTCCAGAGTGCAGCTATTGGTGCTTTGCAGGAG 285
H3mm15 CAGGACTTCAAAACAGATCTGCGCTTCCAGAGTGCAGCTATTGGTGCTTTGCAGGAG 285
H3mm16 CAGGACTTCAAAACAGATCTGCGCTTCCAGAGTGCAGCTATTGGTGCTTTGCAGGAG 285
H3mm13 CAGGACTTCAAAACAGATCTGCGCTTCCAGAGTGCAGCTATTGGTGCTTTGCAGGAG 285
H3mm7 CAGGACTTCAAAACAGATCTGCGCTTCCAGAGTGCAGCTATTGGTGCTTTGCAAGAG 285
H3mm12 CAGGACTTCAAAACAGATCTGCGCTTCCAGAGTGCAGCTATTGGTGCTTTGCAGGAG 285
H3mm6 CAGGACTTCAAAACAGATCTGCGCTTCCAGAGTGCAGCTATTGGTGCTTTGCAGGAG 285
H3mm18 CAGGACTTCAAAACAGATCTGTGCTTCCAGAGTGCAGCTATTGGTGCTTTGCAGGAG 285
H3mm17 CAGGACTTCAAAACAGATCTGCGCTTTCAGAGTTCAGCTATTGGTGCTTTGCAGGAG 285
H3mm10 CAGGACTTCAAAACAGATCTGCGCTTCCAGAATGCAGTTATTGGTGCTTTGCAGGAG 285
H3mm14 CAGGTCTTCAAAACAGATCTGCGCTTCCAGAGTGCAGCTATTGGTGCTTTGCAGGAG 285
H3mm9 CAGGACTTCAAAACAGATCTGCGCTTCCAGAGTGCAGCTATTGGTGCTTTGTGGAAG 285
Consensus
CAGGACTTCAAAACAGATCTGCGCTTCCAGAGTGCAGCTATTGGTGCTTTGCAGGAG
Hist1h3a GCCTGTGAGGCCTACCTTGTGGGTCTGTTTGAGGACACCAACCTGTGCGCCATCCAC 342
Hist2h3b GCGAGCGAGGCCTACCTTGTGGGTCTGTTTGAGGACACCAACCTGTGCGCCATCCAC 342
H3 t GCCTGCGAGTCGTACCTCGTGGGGCTGTTCGAAGACACGAACCTGTGCGCTATCCAC 342
H3mm8 GCTAGCGAAGCATACCTGGTGGGGTTGTTTGAAGATACCAATCTGTGTGCCATCCAC 342
H3f3b GCTAGCGAAGCATACCTGGTGGGGTTGTTTGAAGATACCAATCTGTGTGCCATCCAC 342
H3f3a GCAAGTGAGGCCTATCTGGTTGGCCTTTTTGAAGATACCAATCTGTGTGCTATCCAT 342
H3mm11 GCAAGTGAGGCCTATCTGGTTGGCCTTTTTGAAGATACCAATCTGTGTGCTATCCAT 342
H3mm15 GCAAGTGAGGCCTACCAGGTTGGCCTTTTTGAAGATACCAATCTGTGTGCTGTCCAT 342
H3mm16 GCAAGTGAGGCCTATCTGGTTGGTCTTTTTGAAGATACCAATCTGTGTGCTATCCAT 342
H3mm13 GCAAGTGAGGCCTATCTGGTTGGCCTTTTTGAAGATACCAATCTGTGTGCTATCCAT 342
H3mm7 GCAAGTGAGGCCTATCTGGTTGGCCTTTTTGAAGATACCAATCTGTGTGCTATCCAT 342
H3mm12 GCAAGTGGGGCCTTTCTGGTTGGCCTTTTTGAAGATACCAATCTGTGTGCTATCCAT 342
H3mm6 GCAAGTGAGGCCTACCAGGTTGGCCTTTTTGAAGATACCAATCTGTGTGCTATCCAT 342
H3mm18 GCAAGTGAGGCCTATCTGGTTGGCCTTTTTGAAGATACCAATCTGTGTGCTATCCAT 342
H3mm17 GCAAGTGAGACCTATCTGGTTGGCCTTTTTGAAGATACCAATCTGTGTGCTATCCAT 342
H3mm10 GCAAGTGAGGCCTATCTGGTTGGCCTTTTTGAAGATACCACTCTGTGTGCTATCCAT 342
H3mm14 GCAAGTGAGGCCTATCTGGTTGGCCTTTTTGAAGATACCAATCTGTGTGCTATCCAT 342
H3mm9 GCAAGTGAGGCCTATCTGGTTGGCCTTTTTGAAGATACCAACCTGTGTGCTATCCAT 342
Consensus
GCAAGTGAGGCCTATCTGGTTGGCCTTTTTGAAGATACCAATCTGTGTGCTATCCAT

```
Hist1h3a   GCCAAGCGTGTCACCATCATGCCCAAG-GACATCCAACTGGCCCGCCGCATCCGCGG 398
Hist2h3b   GCCAAGCGTGTCACCATCATGCCCAAG-GACATCCAGCTCGCCCGCCGCATTCGTGG 398
H3 t       GCCAAGCGGGTGACCATCATGCCCAAA-GACATCCAGTTGGCTCGGCGCATCCGCGG 398
H3mm8      GCCAAG--------------------------------------------------- 348
H3f3b      GCCAAGAGAGTCACCATCATGCCCAAA-GACATCCAGTTGGCTCGCCGGATACGGGG 398
H3f3a      GCCAAACGTGTAACAATTATGCCAAAA-GATATCCAGCTAGCACGCCGCATACGCGG 398
H3mm11     GCCAAACGTGTAACAATTATGCCAAAA-GATATCCAGCTAGCACGCCGCATACGCGG 398
H3mm15     GCCAAACGTGTAACAATTATGCCAAAA-GATATCCAGCTAGCATGCCGCATACGCGG 398
H3mm16     GCCAAACGTGTAACAATTATGCCAAAA-GATATCCAGCTAGCATGCCGCATACGCAG 398
H3mm13     GCCAAACGTGTAACAATTATGCCAAAA-GATATCCAGCTAGCACGCCGCATATGCGG 398
H3mm7      GCCAAACGTGTAACAATTATGCCAAAA-GATATCCAGCTAGCACGCCGCATACGCGG 398
H3mm12     GCCAAACGTGTAACAATTATGCCAAAA-GATATCCAGCTAGCACGCCGCATACGCGG 398
H3mm6      GCCAAACATGTAACAATTATGCCAAAA-GATATCCAGCTAGCAAGCCACATACATGG 398
H3mm18     GCCAAACGTGTAACAATTATGCCAAAA-GATACCCAACTAGCAGGCTACATATGCAG 398
H3mm17     GCCAAACGTGTAACAATTATGCCAAAA-GATATCCAGCTAGCTAGC---ATACGTGG 395
H3mm10     GCCAAACGTGTAACAATTATGCCAAAA-GAGATCCAGCTAGCACACTGCATC-GTGG 397
H3mm14     GCCAAACGTGTAGTAACTATGCCAAAA-GATATCCAGCTAACACTCCGCATATGCGG 398
H3mm9      GCCAAATGTGTAACAATTACGCCAAAAAAATTTCCAGCTAACACGCTGCATATGTGG 399

Consensus
           GCCAAACGTGTAACAATTATGCCAAAA-GATATCCAGCTAGCACGCCGCATACGCGG

Hist1h3a   GGAGAGGGCGTAA-------------------------------------------- 411
Hist2h3b   GGAGAGGGCTTAA-------------------------------------------- 411
H3 t       AGAACGGGCTTAG-------------------------------------------- 411
H3mm8      ---------------------------------------------------------
H3f3b      GGAGAGAGCTTAA-------------------------------------------- 411
H3f3a      AGAACGTGCTTAA-------------------------------------------- 411
H3mm11     AGAACGTGCTTAA-------------------------------------------- 411
H3mm15     AGAACGTGCTTAA-------------------------------------------- 411
H3mm16     AGAACGTGCTTAA-------------------------------------------- 411
H3mm13     AGAACGTGCTTAA-------------------------------------------- 411
H3mm7      AGAACGTGCTTAA-------------------------------------------- 411
H3mm12     AGAACGTGCTTAA-------------------------------------------- 411
H3mm6      AGAACGTGCTTAA-------------------------------------------- 411
H3mm18     AGAATGTGCTTAA-------------------------------------------- 411
H3mm17     AGAATGTGCTTAA-------------------------------------------- 408
H3mm10     AGAACGTGCTTAAGAGTCCACTATGA------------------------------- 423
H3mm14     AGTACATGTTCAAGAGTCCACTATGAGGGGAAACATTTCATTCTCAAAAATTTTTT 455
H3mm9      AGAACATTCTTAAGAGTCCACTATGAGGGGAAACATTTCATTCTCAAAAAAA-AATT 455

Consensus
           AGAACGTGCTTAAGAGTCCACTATGAGGGGAAACATTTCATTCTCAAAAA-----TT

Hist1h3a   -------------------------
Hist2h3b   -------------------------
H3 t       -------------------------
H3mm8      -------------------------
H3f3b      -------------------------
H3f3a      -------------------------
H3mm11     -------------------------
H3mm15     -------------------------
H3mm16     -------------------------
H3mm13     -------------------------
H3mm7      -------------------------
H3mm12     -------------------------
H3mm6      -------------------------
H3mm18     -------------------------
H3mm17     -------------------------
H3mm10     -------------------------
H3mm14     TTCCTCTTCTTCCTGTTATCAGTAG 480
H3mm9      TTCCTCTTCTTCCTGTTATCAGTAG 480

Consensus
           TTCCTCTTCTTCCTGTTATCAGTAG
```

Figure S2

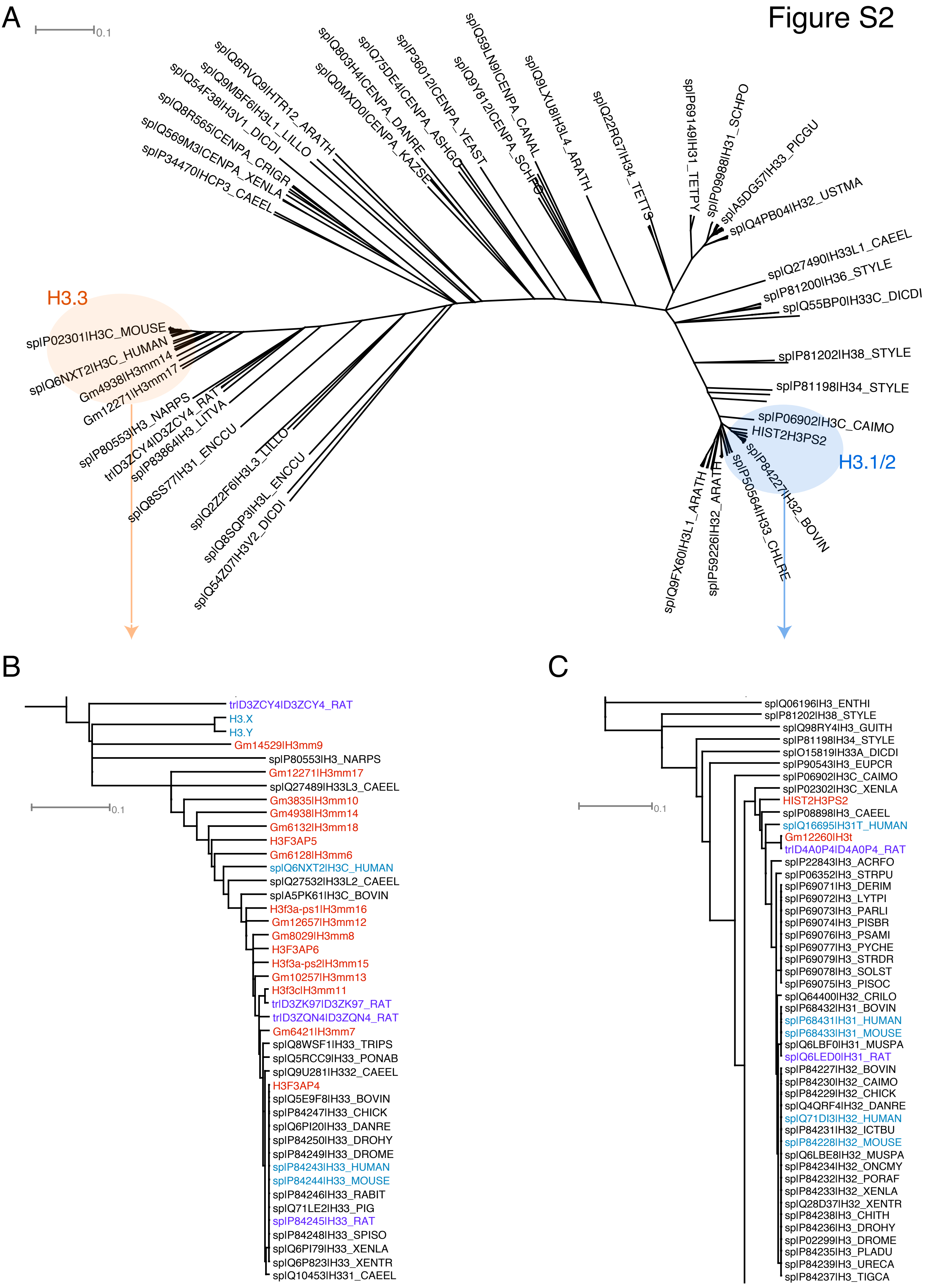
A
0.1
sp|Q8RVQ9|HTR12_ARATH
sp|Q9MBF6|H3L1_LILLO
sp|Q54F38|H3V1_DICDI
sp|Q8R565|CENPA_CRIGR
sp|Q569M3|CENPA_XENLA
sp|P34470|HCP3_CAEEL
sp|Q803H4|CENPA_DANRE
sp|Q0MXD0|CENPA_KAZSE
sp|Q75DE4|CENPA_ASHGO
sp|P36012|CENPA_YEAST
sp|Q9Y812|CENPA_SCHPO
sp|Q59LN9|CENPA_CANAL
sp|Q9LXU8|H3L4_ARATH
sp|Q22RG7|H34_TETTS
sp|P69149|H31_TETPY
sp|P09988|H31_SCHPO
sp|A5DG57|H33_PICGU
sp|Q4PB04|H32_USTMA
sp|Q27490|H33L1_CAEEL
sp|P81200|H36_STYLE
sp|Q55BP0|H33C_DICDI
sp|P81202|H38_STYLE
sp|P81198|H34_STYLE
sp|P06902|H3C_CAIMO
HIST2H3PS2
H3.1/2
sp|P84227|H32_BOVIN
sp|P50564|H33_CHLRE
sp|P59226|H32_ARATH
sp|Q9FX60|H3L1_ARATH
H3.3
sp|P02301|H3C_MOUSE
sp|Q6NXT2|H3C_HUMAN
Gm4938|H3mm14
Gm12271|H3mm17
sp|P80553|H3_NARPS
tr|D3ZCY4|D3ZCY4_RAT
sp|P83864|H3_LITVA
sp|Q8SS77|H31_ENCCU
sp|Q2Z2F6|H3L3_LILLO
sp|Q8SQP3|H3L_ENCCU
sp|Q54Z07|H3V2_DICDI
B
0.1
tr|D3ZCY4|D3ZCY4_RAT
H3.X
H3.Y
Gm14529|H3mm9
sp|P80553|H3_NARPS
Gm12271|H3mm17
sp|Q27489|H33L3_CAEEL
Gm3835|H3mm10
Gm4938|H3mm14
Gm6132|H3mm18
H3F3AP5
Gm6128|H3mm6
sp|Q6NXT2|H3C_HUMAN
sp|Q27532|H33L2_CAEEL
sp|A5PK61|H3C_BOVIN
H3f3a-ps1|H3mm16
Gm12657|H3mm12
Gm8029|H3mm8
H3F3AP6
H3f3a-ps2|H3mm15
Gm10257|H3mm13
H3f3c|H3mm11
tr|D3ZK97|D3ZK97_RAT
tr|D3ZQN4|D3ZQN4_RAT
Gm6421|H3mm7
sp|Q8WSF1|H33_TRIPS
sp|Q5RCC9|H33_PONAB
sp|Q9U281|H332_CAEEL
H3F3AP4
sp|Q5E9F8|H33_BOVIN
sp|P84247|H33_CHICK
sp|Q6PI20|H33_DANRE
sp|P84250|H33_DROHY
sp|P84249|H33_DROME
sp|P84243|H33_HUMAN
sp|P84244|H33_MOUSE
sp|P84246|H33_RABIT
sp|Q71LE2|H33_PIG
sp|P84245|H33_RAT
sp|P84248|H33_SPISO
sp|Q6PI79|H33_XENLA
sp|Q6P823|H33_XENTR
sp|Q10453|H331_CAEEL
C
0.1
sp|Q06196|H3_ENTHI
sp|P81202|H38_STYLE
sp|Q98RY4|H3_GUITH
sp|P81198|H34_STYLE
sp|O15819|H33A_DICDI
sp|P90543|H3_EUPCR
sp|P06902|H3C_CAIMO
sp|P02302|H3C_XENLA
HIST2H3PS2
sp|P08898|H3_CAEEL
sp|Q16695|H31T_HUMAN
Gm12260|H3t
tr|D4A0P4|D4A0P4_RAT
sp|P22843|H3_ACRFO
sp|P06352|H3_STRPU
sp|P69071|H3_DERIM
sp|P69072|H3_LYTPI
sp|P69073|H3_PARLI
sp|P69074|H3_PISBR
sp|P69076|H3_PSAMI
sp|P69077|H3_PYCHE
sp|P69079|H3_STRDR
sp|P69078|H3_SOLST
sp|P69075|H3_PISOC
sp|Q64400|H32_CRILO
sp|P68432|H31_BOVIN
sp|P68431|H31_HUMAN
sp|P68433|H31_MOUSE
sp|Q6LBF0|H31_MUSPA
sp|Q6LED0|H31_RAT
sp|P84227|H32_BOVIN
sp|P84230|H32_CAIMO
sp|P84229|H32_CHICK
sp|Q4QRF4|H32_DANRE
sp|Q71DI3|H32_HUMAN
sp|P84231|H32_ICTBU
sp|P84228|H32_MOUSE
sp|Q6LBE8|H32_MUSPA
sp|P84234|H32_ONCMY
sp|P84232|H32_PORAF
sp|P84233|H32_XENLA
sp|Q28D37|H32_XENTR
sp|P84238|H3_CHITH
sp|P84236|H3_DROHY
sp|P02299|H3_DROME
sp|P84235|H3_PLADU
sp|P84239|H3_URECA
sp|P84237|H3_TIGCA

Figure S3

A

```
H3.1               MARTKQTARKSTGGKAPRKQLATKAARKSAPATGGVKKPHRYRPGTVALREIRRYQKSTE
Human H3T          MARTKQTARKSTGGKAPRKQLATKVARKSAPATGGVKKPHRYRPGTVALREIRRYQKSTE
Mouse H3t          MARTKQTARKSTGGKAPRKQLATKVARKSAPATGGVKKPHRYHPGTVALREIRRYQKSTE
                   ************************.*****************:*****************

H3.1               LLIRKLPFQRLVREIAQDFKTDLRFQSSAVMALQEACEAYLVGLFEDTNLCAIHAKRVTI
Human H3T          LLIRKLPFQRLMREIAQDFKTDLRFQSSAVMALQEACESYLVGLFEDTNLCVIHAKRVTI
Mouse H3t          LLIRKLPFQRLVREIAQDFKTDLRFQSSAVMALQEACESYLVGLFEDTNLCAIHAKRVTI
                   ***********:**************************:************.********

H3.1               MPKDIQLARRIRGERA
Human H3T          MPKDIQLARRIRGERA
Mouse H3t          MPKDIQLARRIRGERA
                   ****************
```

B

```
H2A.Z            MAGGKAGKDSGKAKTKAVSRSQRAGLQFPVGRIHRHLKSRTTSHGRVGATAAVYSAAILE
H2A.Z like       MAGGKAGKDSGKAKTKAVSRSQRAGLQFPVGR----------------------------
H2A.J            --MSGRGKQGGKVRAKAKSRSSRAGLQFPVGRVHRLLRKG-NYAERVGAGAPVYLAAVLE
H2A.J like       ------------------------------MGRVHRLLRKG-NYAERVGAGAPVYPAAVLE
                                               :**

H2A.Z            YLTAEVLELAGNASKDLKVKRITPRHLQLAIRGDEELDSLIK-ATIAGGGVIPHIHKSLI
H2A.Z like       ------------------------PRHLQLAIRGDEELDSLIK-ATIAGGGVIPHIHKSLI
H2A.J            YLTAEILELAGNAARDNKKTRIIPRHLQLAIRNDEELNKLLGRVTIAQGGVLPNIQAVLL
H2A.J like       CL--EILELAGNVARDNKKTRIIPGHLQLAIHNDQKLNKLLGLVTIAQGGVLPNIQAVLL
                                         * ******: *::*:.*:  .*** ***:*.*:  *:

H2A.Z            GKKGQQKTV------------
H2A.Z like       GKKGQQKTV------------
H2A.J            PKKTESQKVKSK---------
H2A.J like       PKKTESQKVKSKCRATTVHSQ
                  ** :.:.*
```

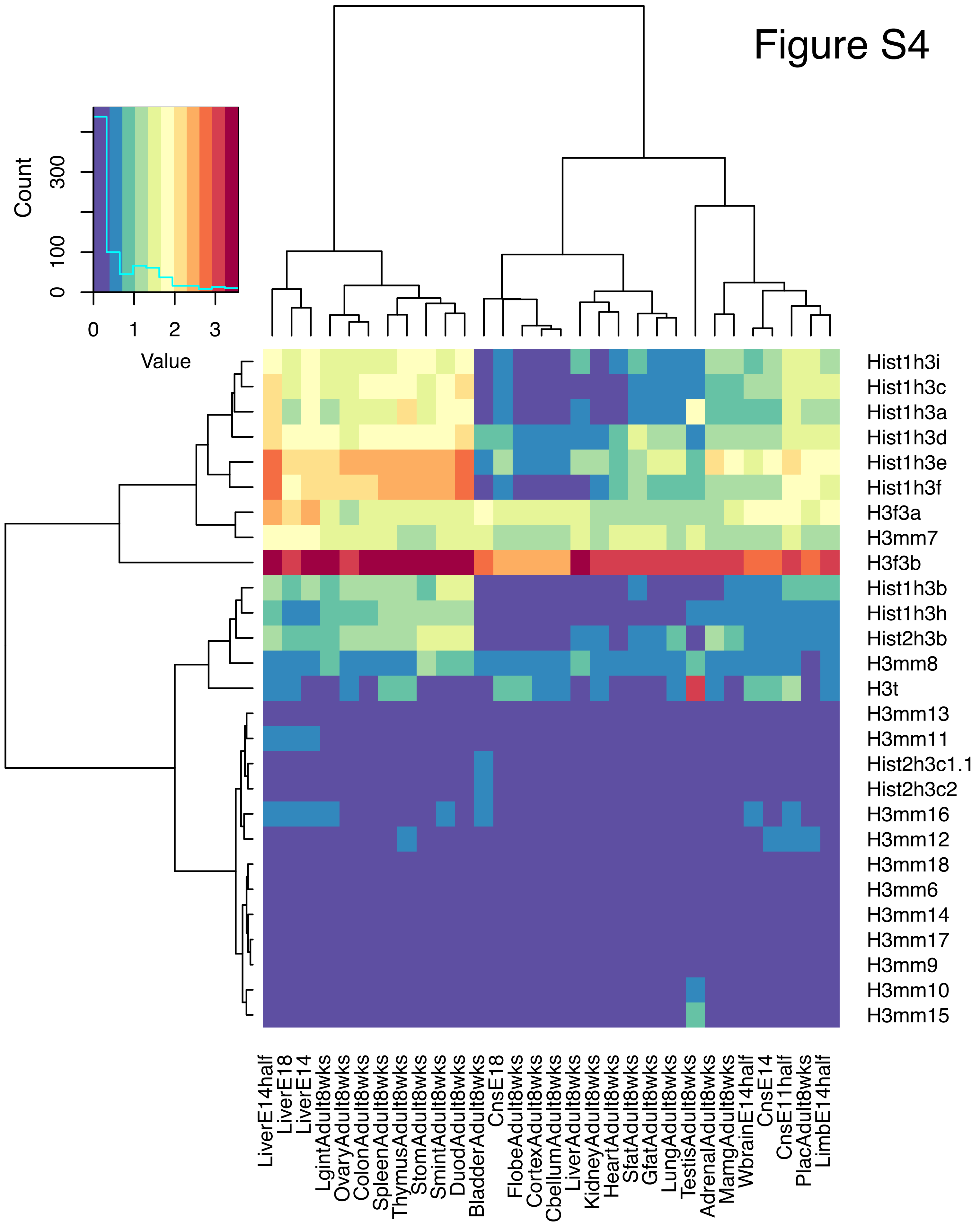
Figure S4
Count
Value
0
100
300
0
1
2
3
Hist1h3i
Hist1h3c
Hist1h3a
Hist1h3d
Hist1h3e
Hist1h3f
H3f3a
H3mm7
H3f3b
Hist1h3b
Hist1h3h
Hist2h3b
H3mm8
H3t
H3mm13
H3mm11
Hist2h3c1.1
Hist2h3c2
H3mm16
H3mm12
H3mm18
H3mm6
H3mm14
H3mm17
H3mm9
H3mm10
H3mm15
LiverE14half
LiverE18
LiverE14
LgintAdult8wks
OvaryAdult8wks
ColonAdult8wks
SpleenAdult8wks
ThymusAdult8wks
StomAdult8wks
SmintAdult8wks
DuodAdult8wks
BladderAdult8wks
CnsE18
FlobeAdult8wks
CortexAdult8wks
CbellumAdult8wks
LiverAdult8wks
KidneyAdult8wks
HeartAdult8wks
SfatAdult8wks
GfatAdult8wks
LungAdult8wks
TestisAdult8wks
AdrenalAdult8wks
MamgAdult8wks
WbrainE14half
CnsE14
CnsE11half
PlacAdult8wks
LimbE14half

Fig. S5A (H3.3)

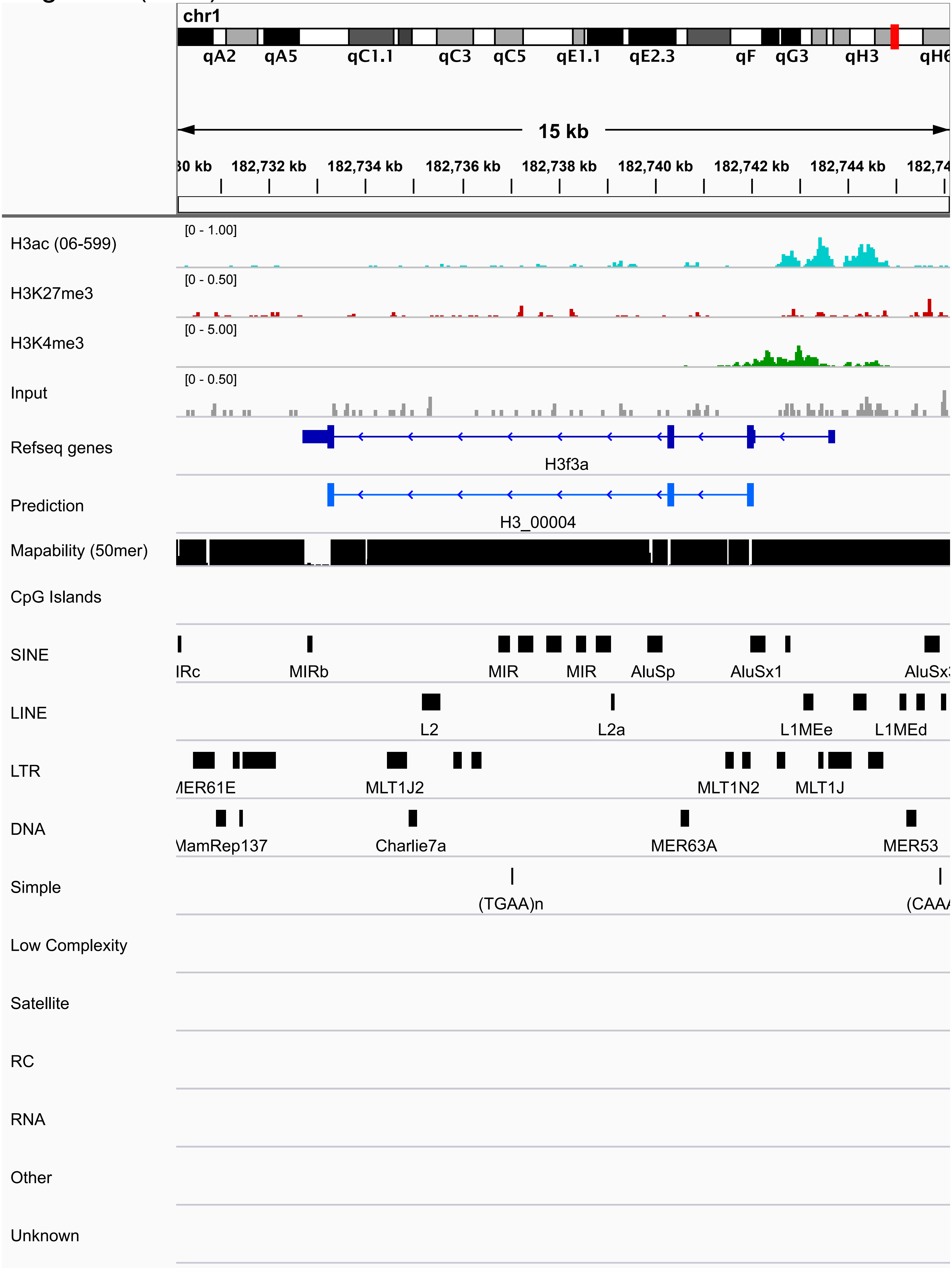
chr1
qA2
qA5
qC1.1
qC3
qC5
qE1.1
qE2.3
qF
qG3
qH3
15 kb
182,732 kb
182,734 kb
182,736 kb
182,738 kb
182,740 kb
182,742 kb
182,744 kb
H3ac (06-599)
[0 - 1.00]
H3K27me3
[0 - 0.50]
H3K4me3
[0 - 5.00]
Input
[0 - 0.50]
Refseq genes
H3f3a
Prediction
H3_00004
Mapability (50mer)
CpG Islands
SINE
MIRb
MIR
MIR
AluSp
AluSx1
LINE
L2
L2a
L1MEe
L1MEd
LTR
MLT1J2
MLT1N2
MLT1J
DNA
Charlie7a
MER63A
MER53
Simple
(TGAA)n
Low Complexity
Satellite
RC
RNA
Other
Unknown

Fig. S5B (H3.3)

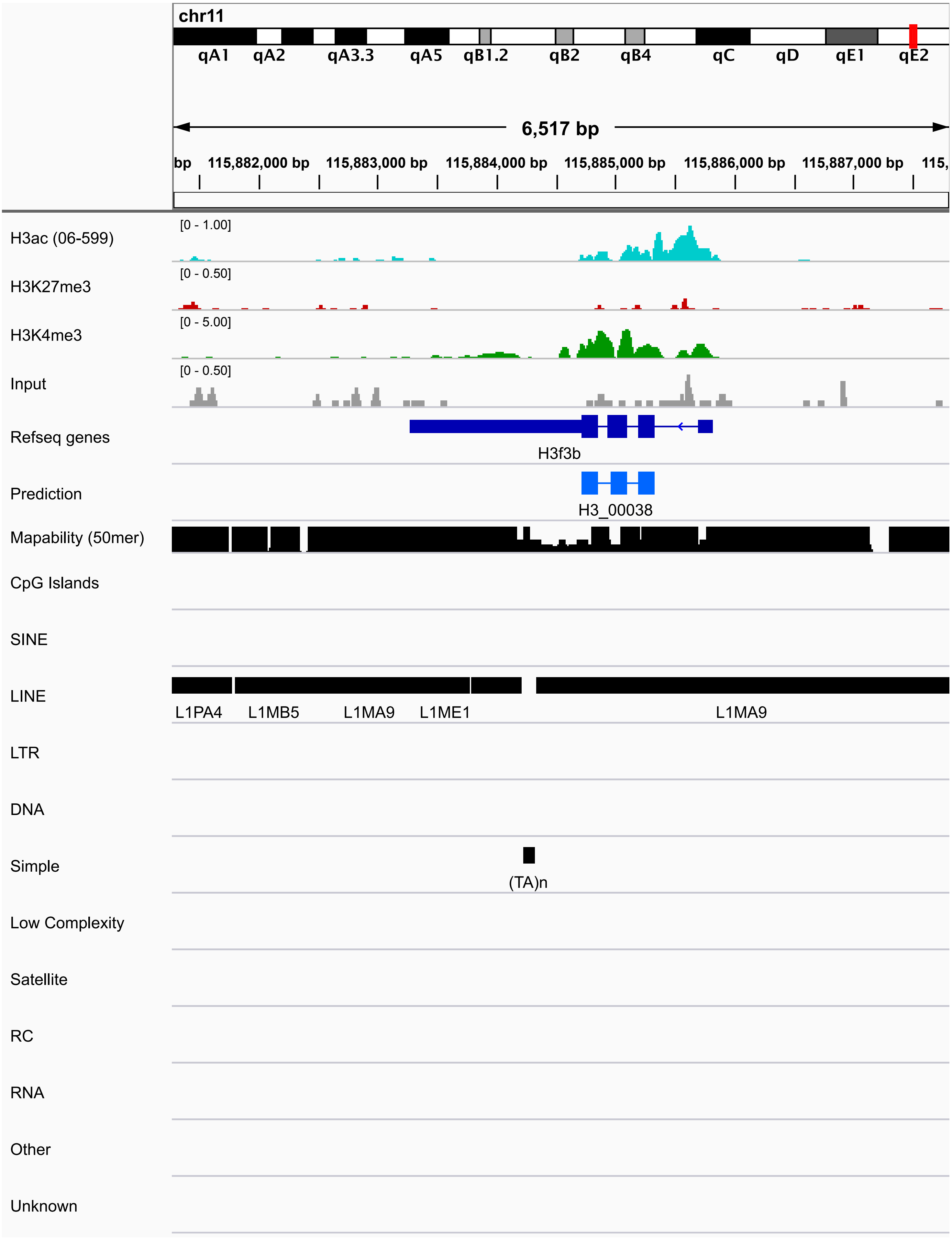
chr11
qA1
qA2
qA3.3
qA5
qB1.2
qB2
qB4
qC
qD
qE1
qE2
6,517 bp
bp
115,882,000 bp
115,883,000 bp
115,884,000 bp
115,885,000 bp
115,886,000 bp
115,887,000 bp
115,
H3ac (06-599)
[0 - 1.00]
H3K27me3
[0 - 0.50]
H3K4me3
[0 - 5.00]
Input
[0 - 0.50]
Refseq genes
H3f3b
Prediction
H3_00038
Mapability (50mer)
CpG Islands
SINE
LINE
L1PA4
L1MB5
L1MA9
L1ME1
L1MA9
LTR
DNA
Simple
(TA)n
Low Complexity
Satellite
RC
RNA
Other
Unknown

Fig. S5C (H3mm8)

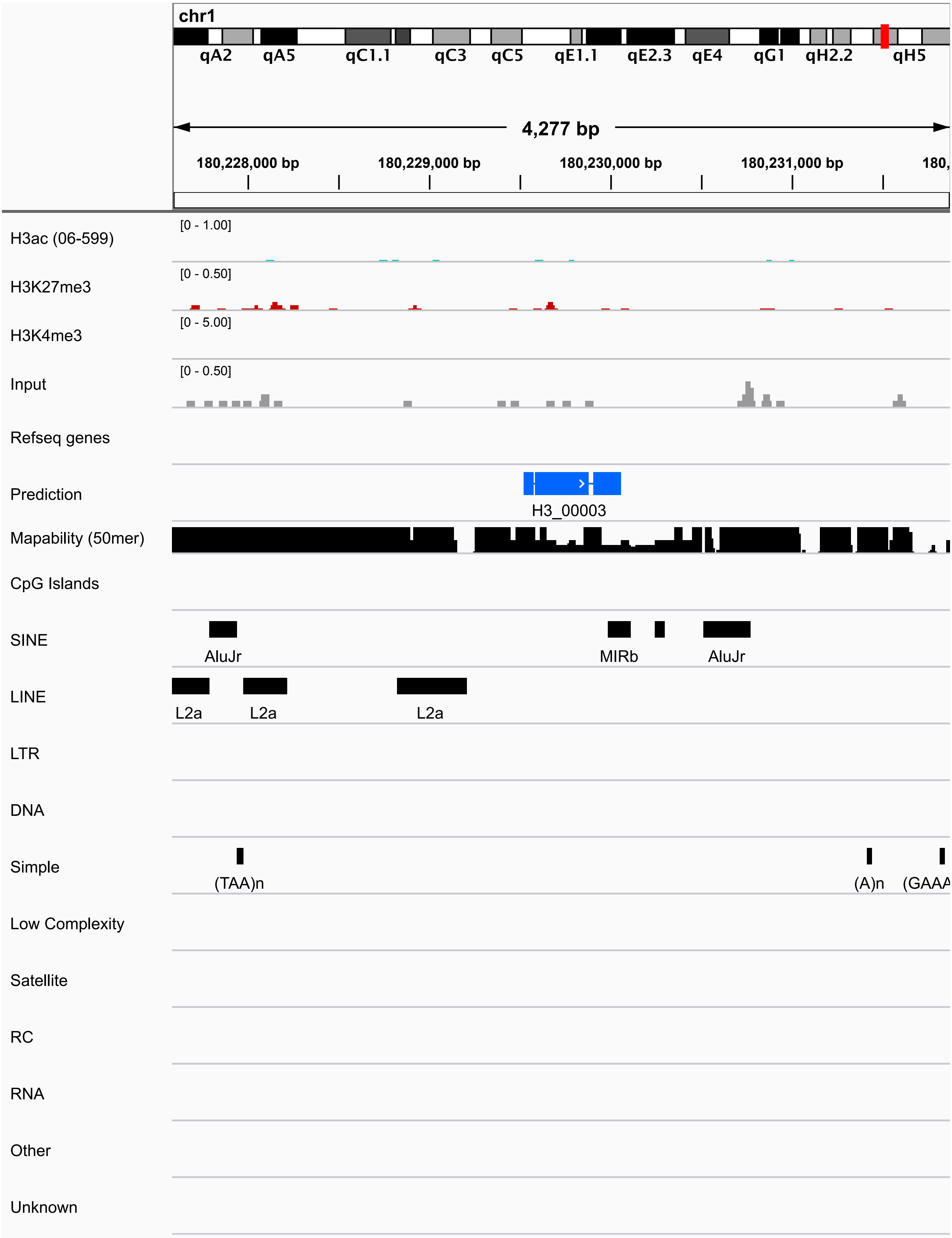

Fig. S5D (H3mm11)

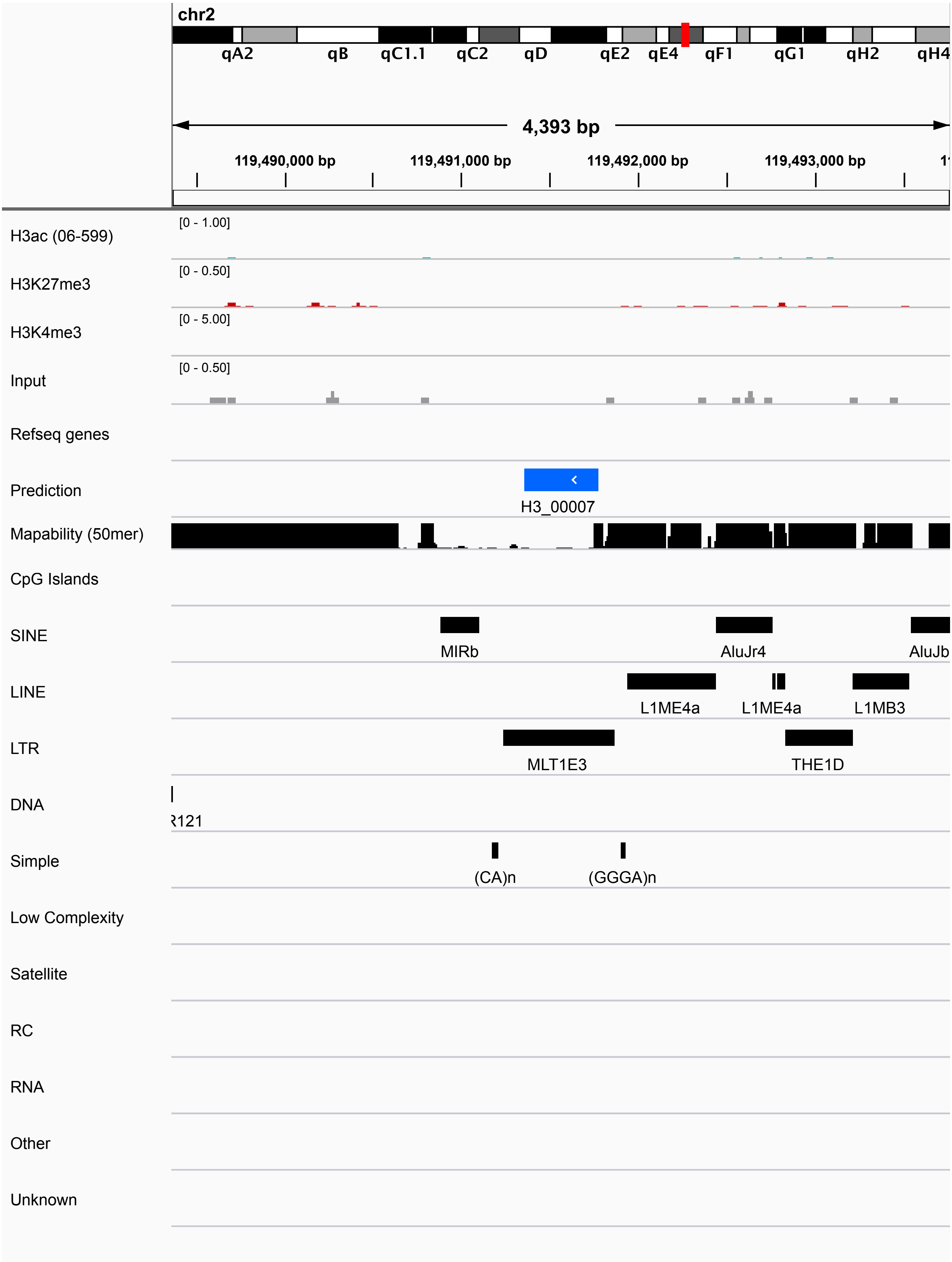

Fig. S5E (H3mm12)

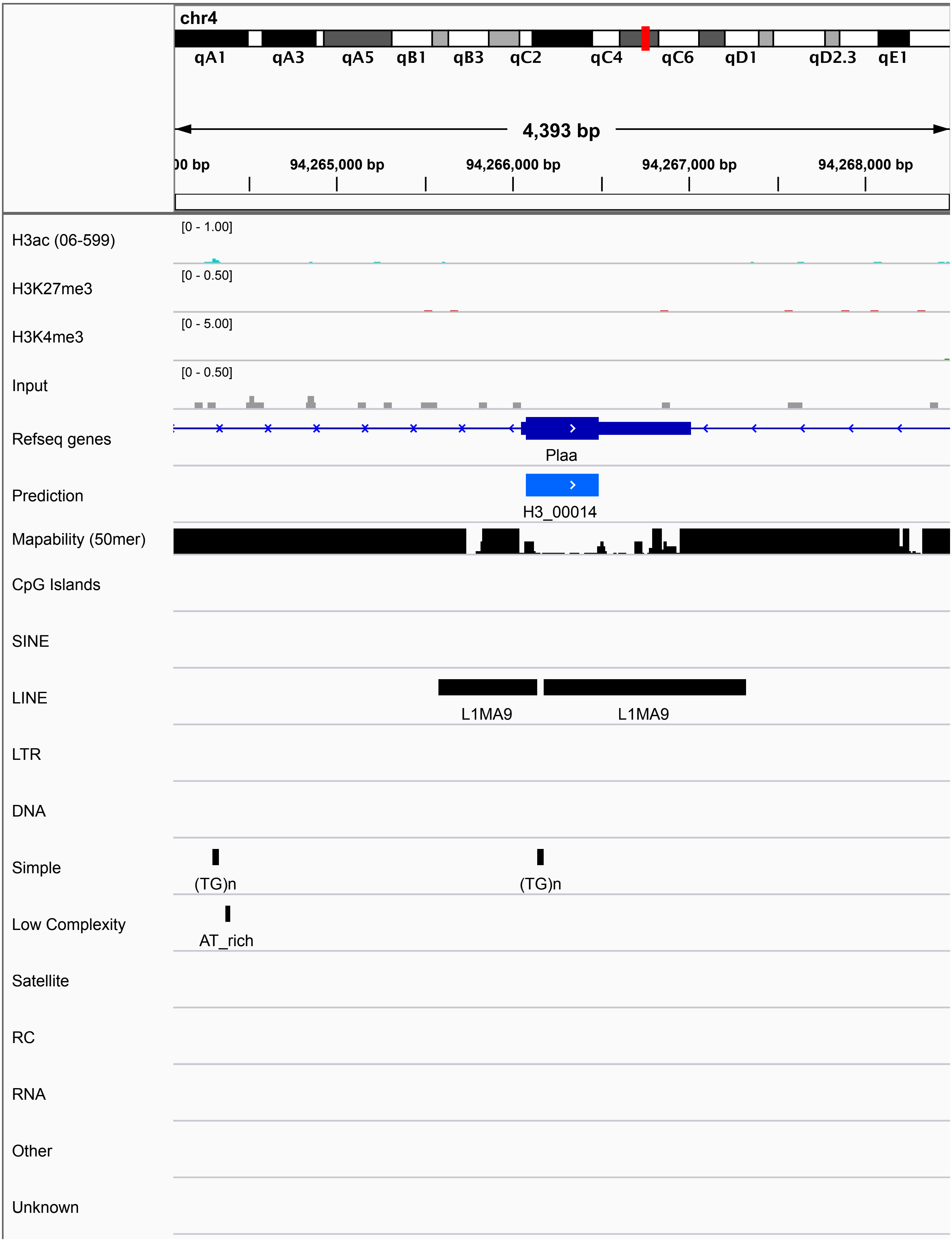

Fig. S5F (H3mm10)

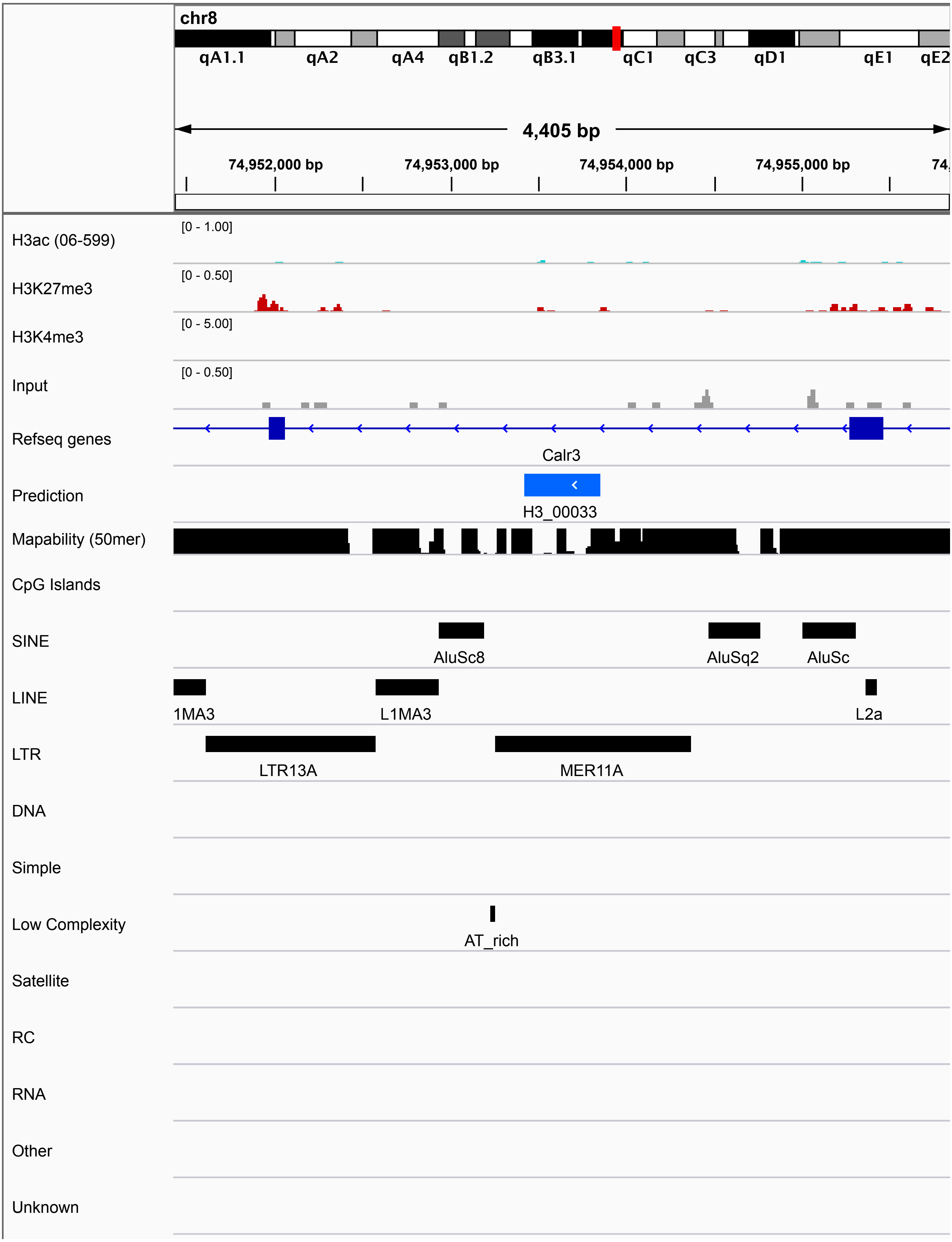

Fig. S5G (H3t)

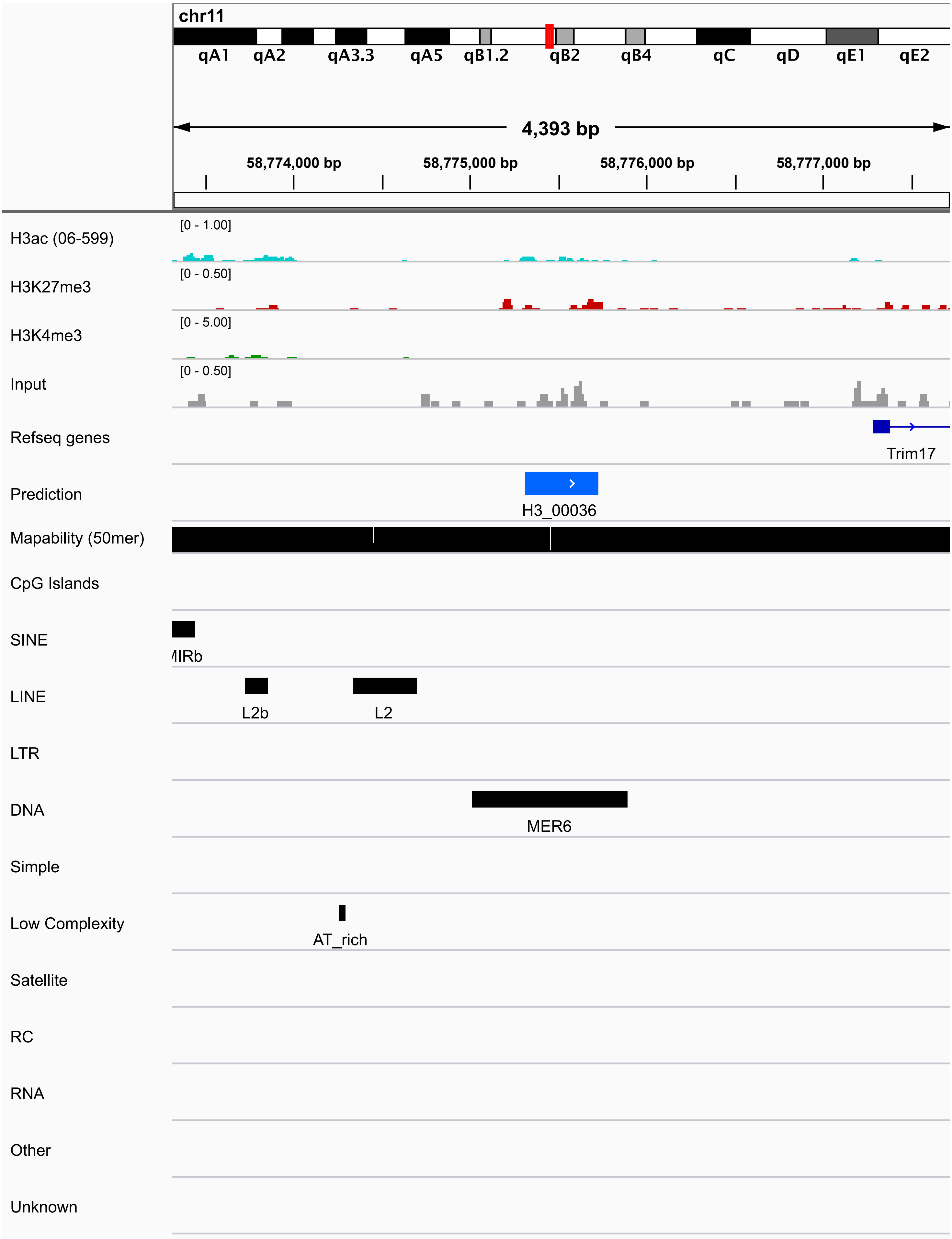

chr11
qA1
qA2
qA3.3
qA5
qB1.2
qB2
qB4
qC
qD
qE1
qE2
4,393 bp
58,774,000 bp
58,775,000 bp
58,776,000 bp
58,777,000 bp
H3ac (06-599)
[0 - 1.00]
H3K27me3
[0 - 0.50]
H3K4me3
[0 - 5.00]
Input
[0 - 0.50]
Refseq genes
Trim17
Prediction
H3_00036
Mapability (50mer)
CpG Islands
SINE
MIRb
LINE
L2b
L2
LTR
DNA
MER6
Simple
Low Complexity
AT_rich
Satellite
RC
RNA
Other
Unknown

Fig. S5H (H3mm17)

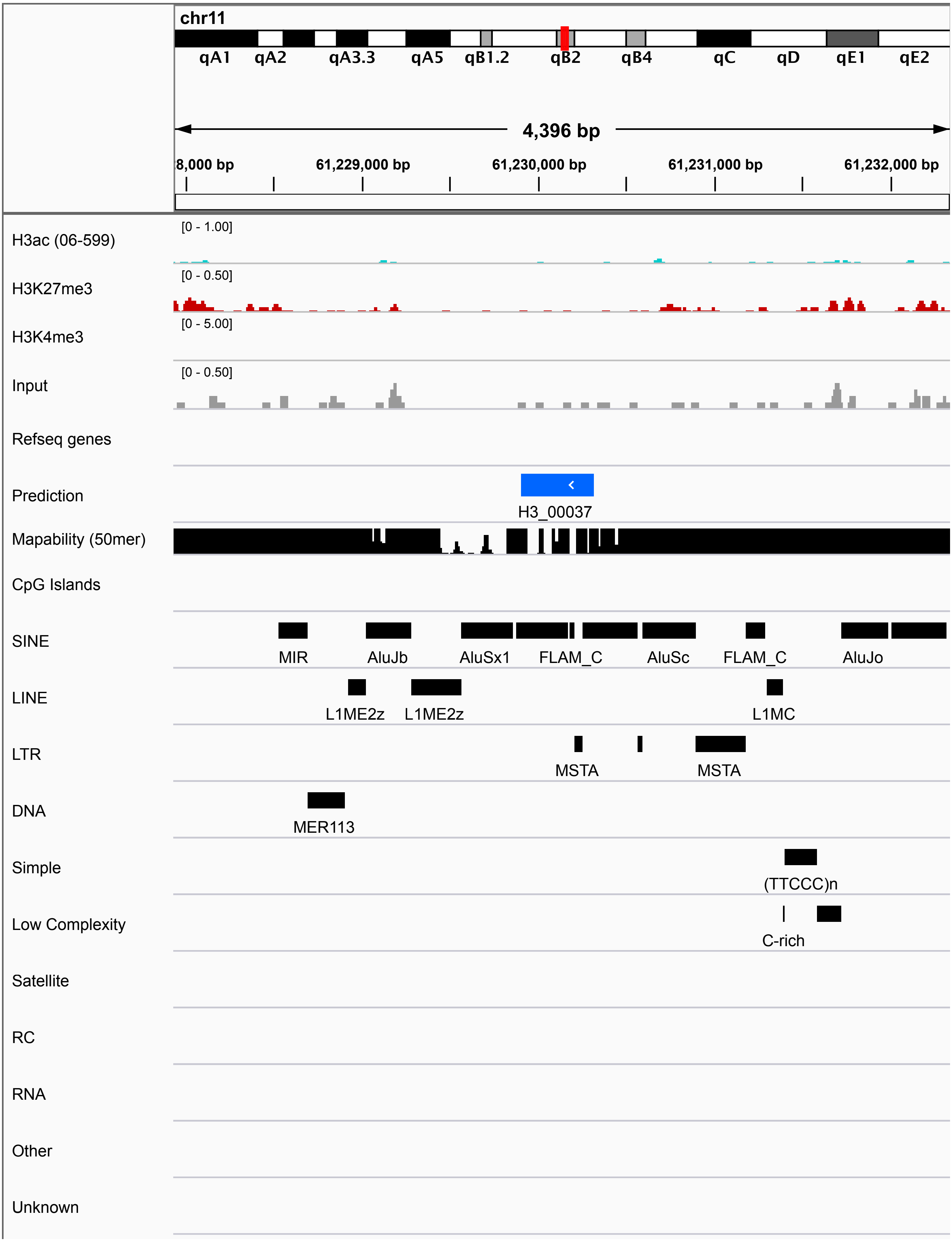

Fig. S5I (H3mm18)

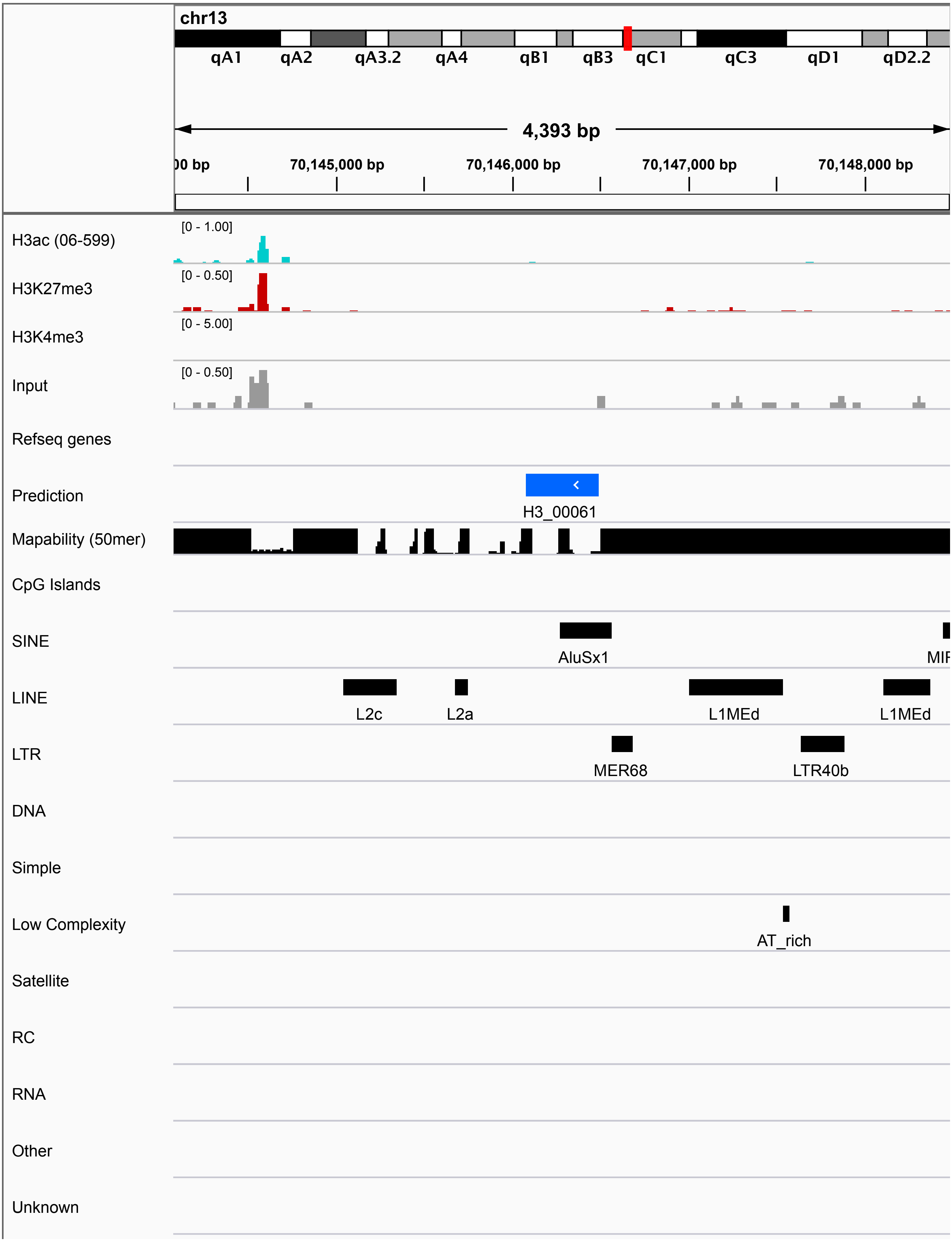

Fig. S5J (H3mm13)

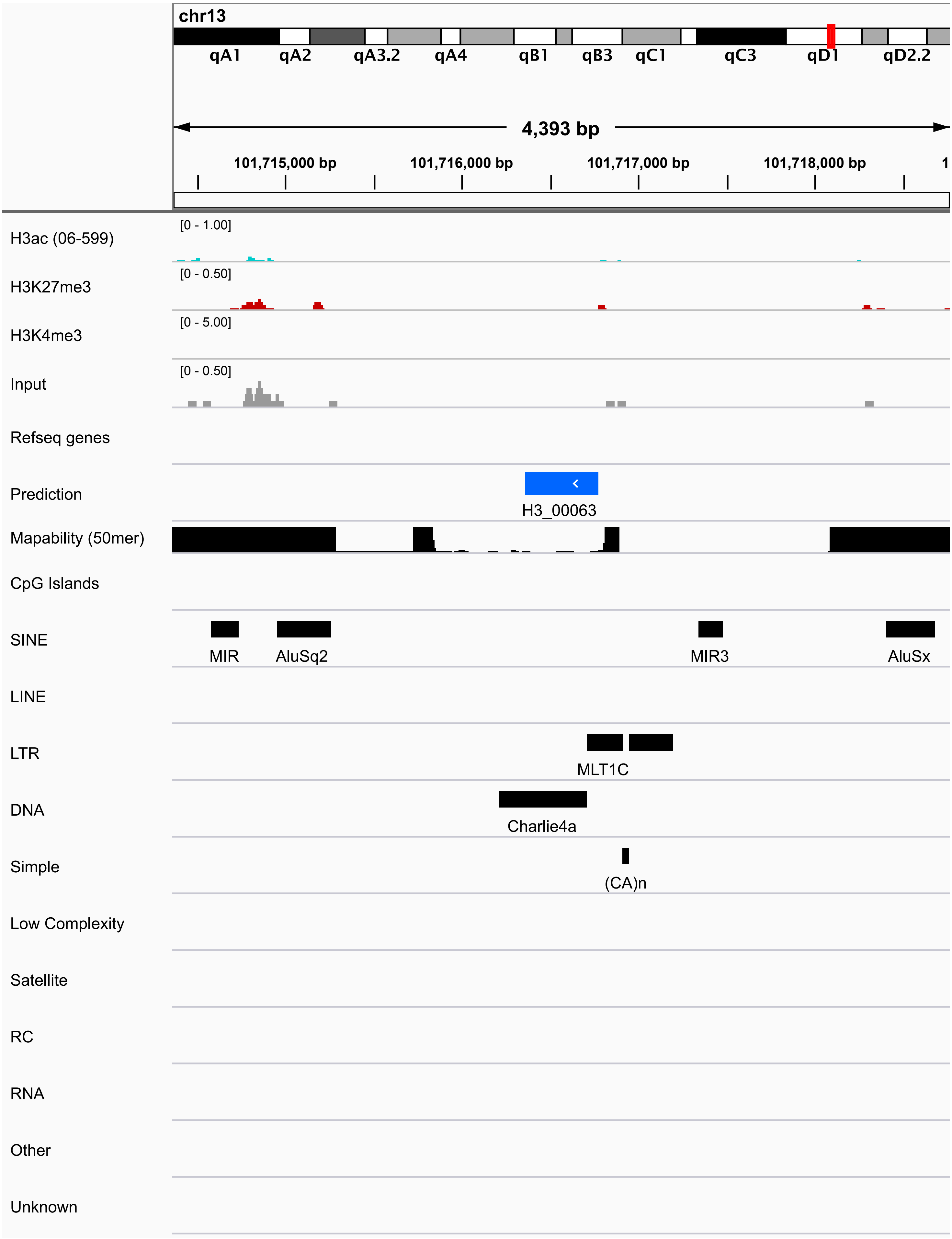

Fig. S5K (H3mm14)

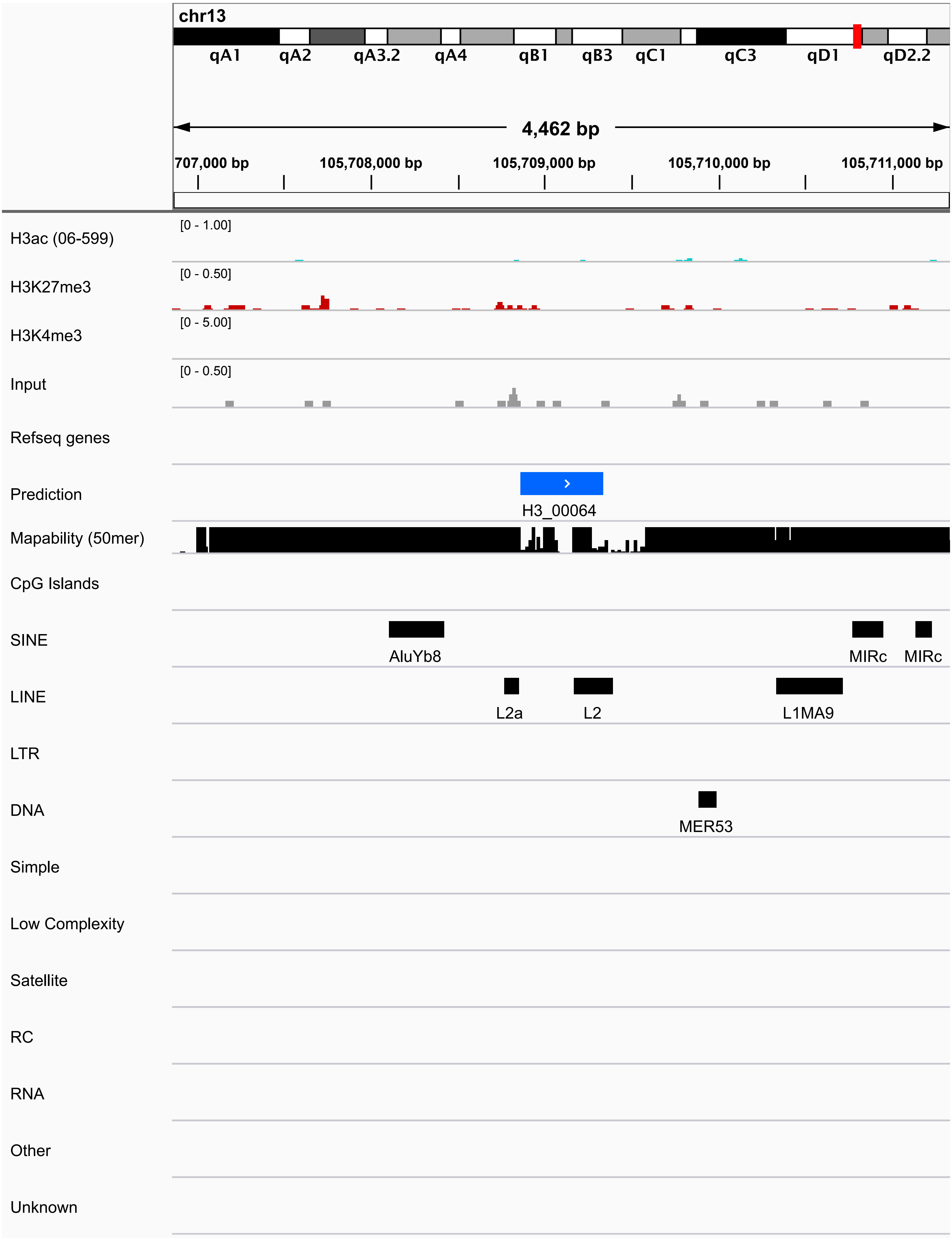

Fig. S5L (H3mm7)

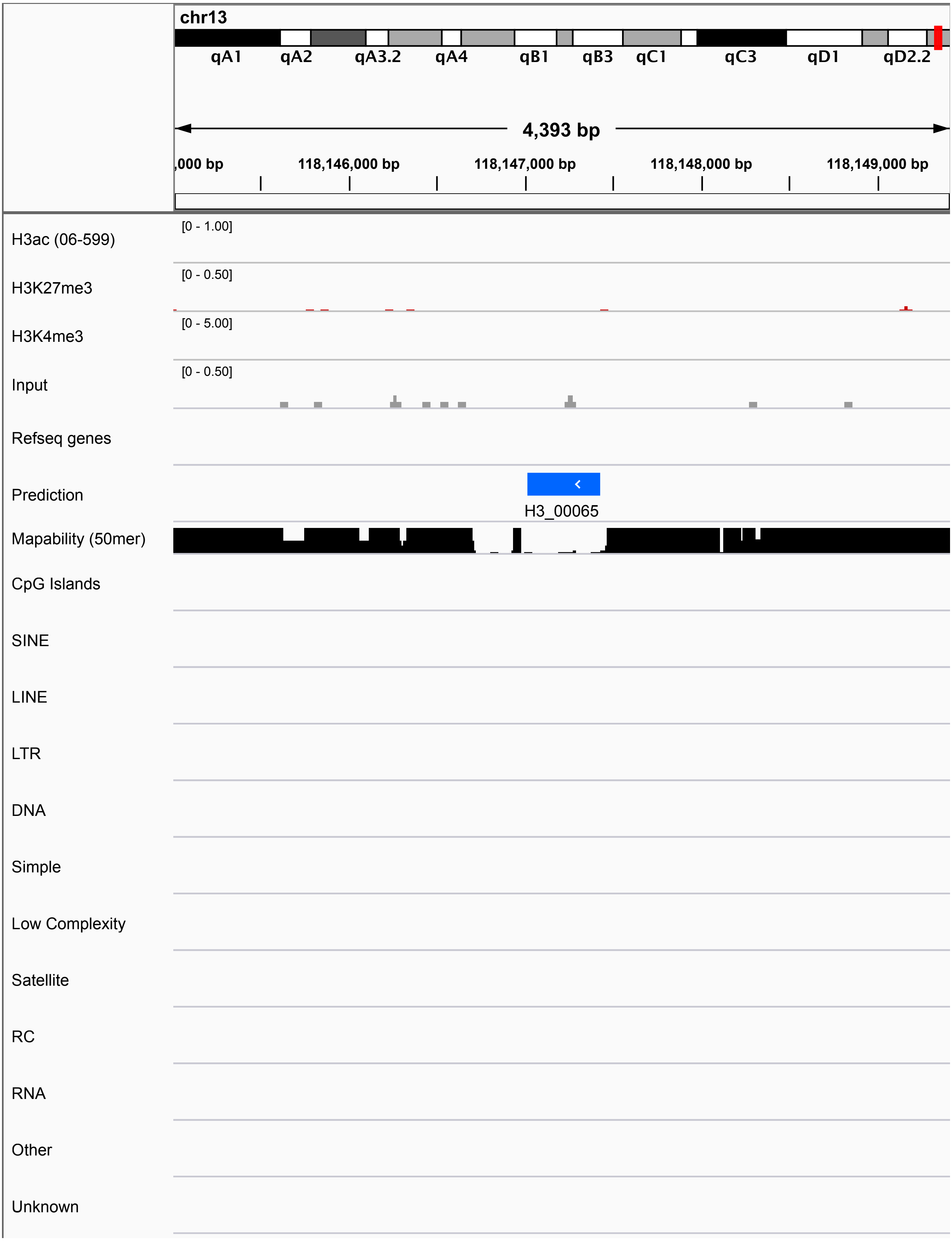

Fig. S5M (H3mm6)

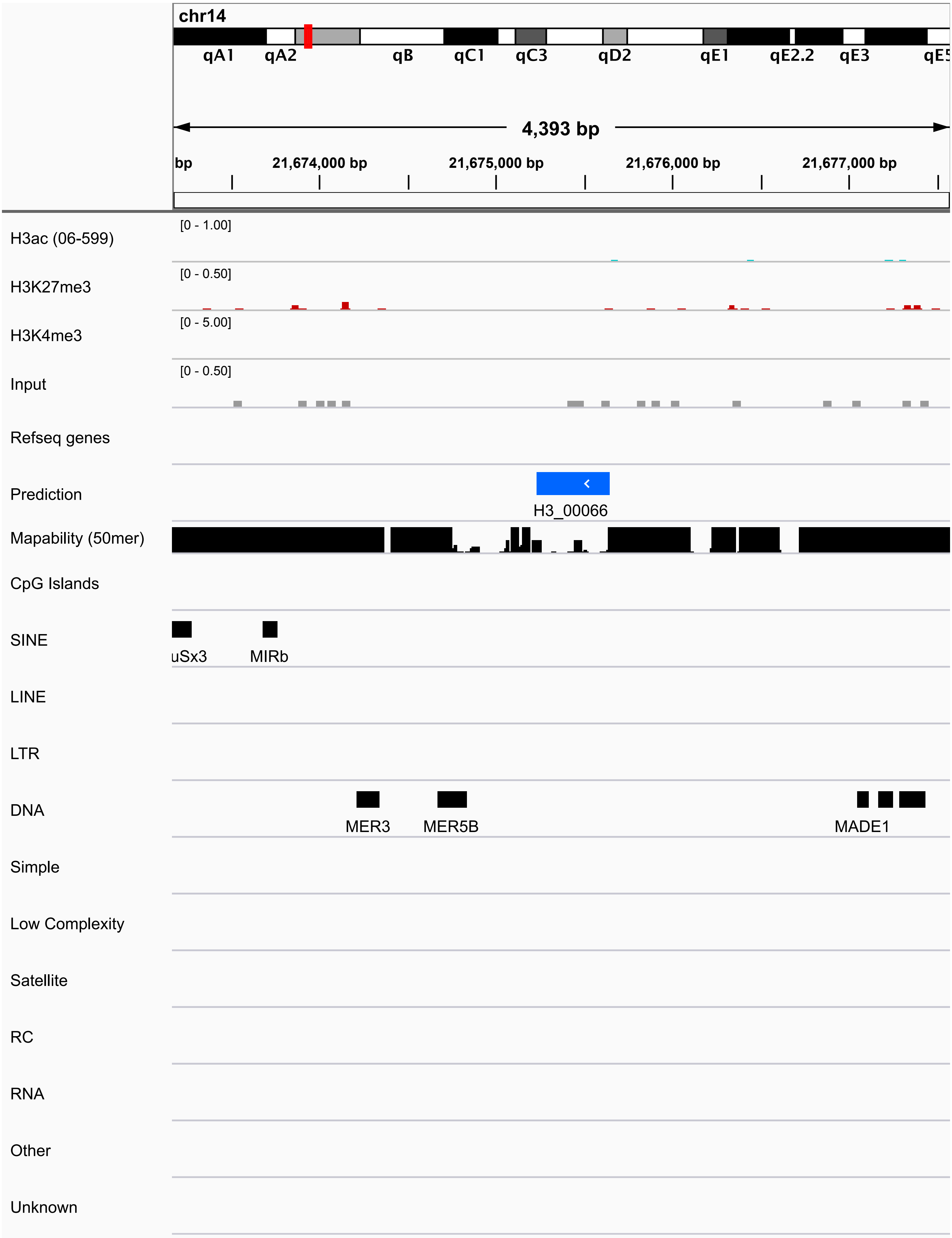

Fig. S5N (H3mm15)

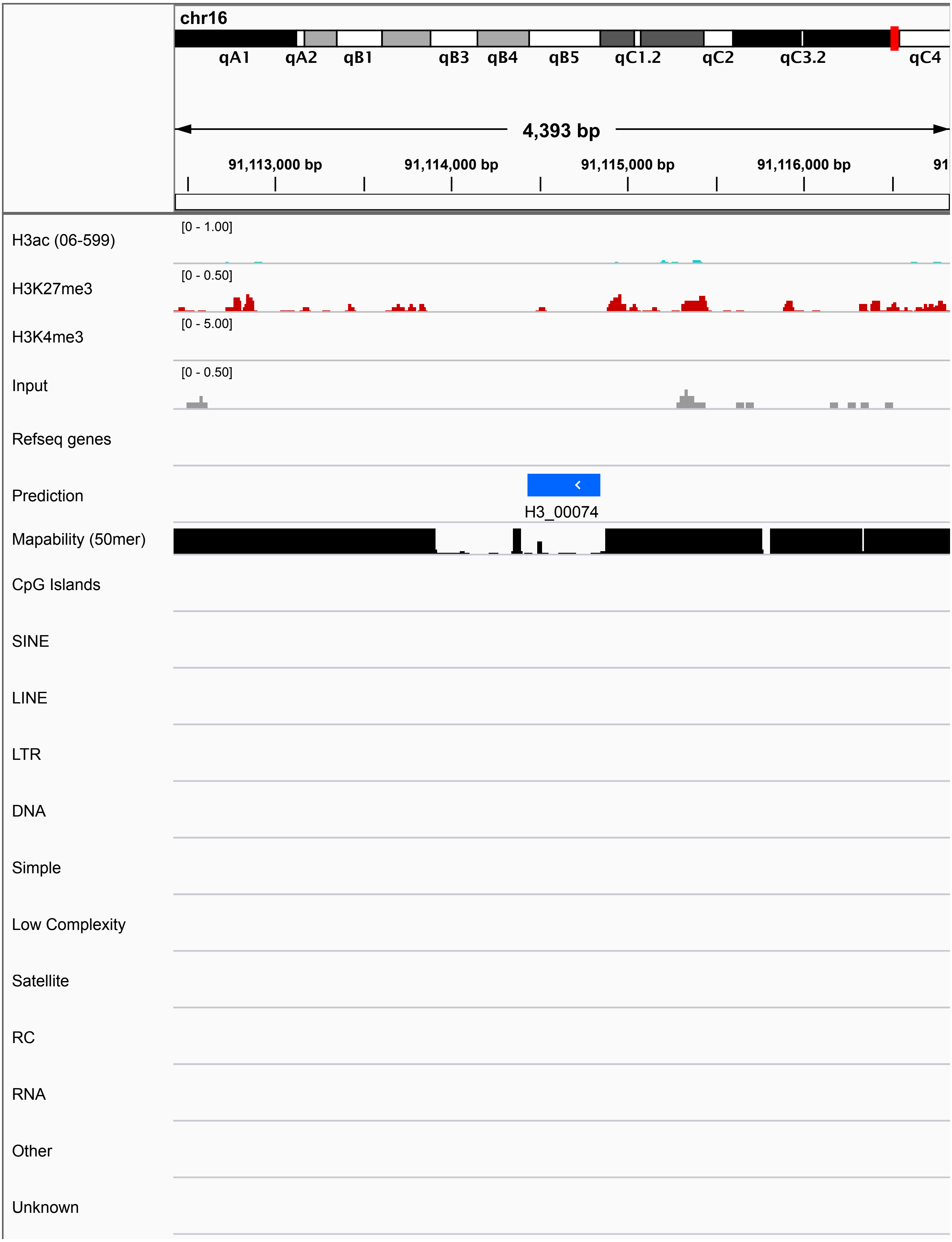

Fig. S5O (H3mm9)

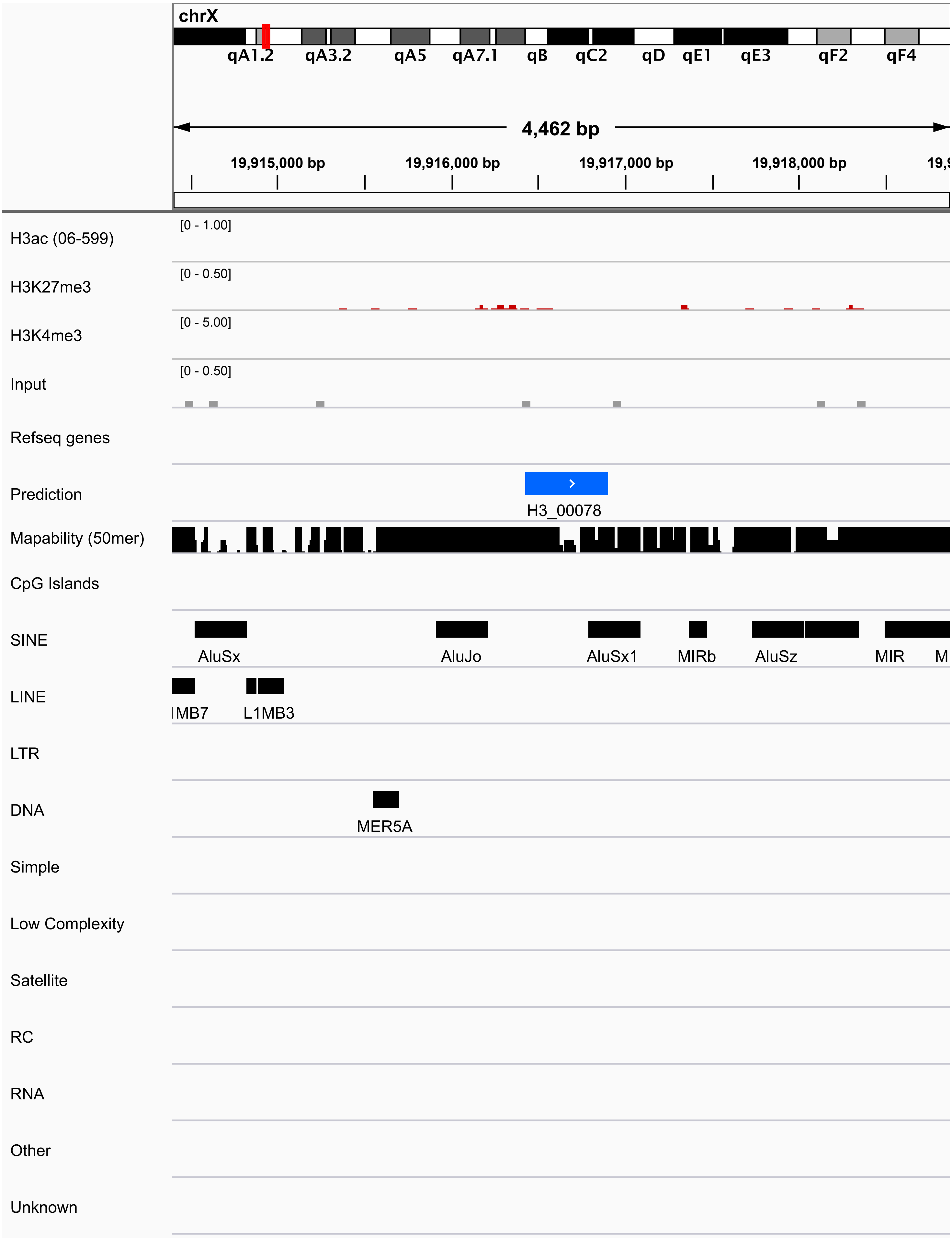

Fig. S5P (H3mm16)

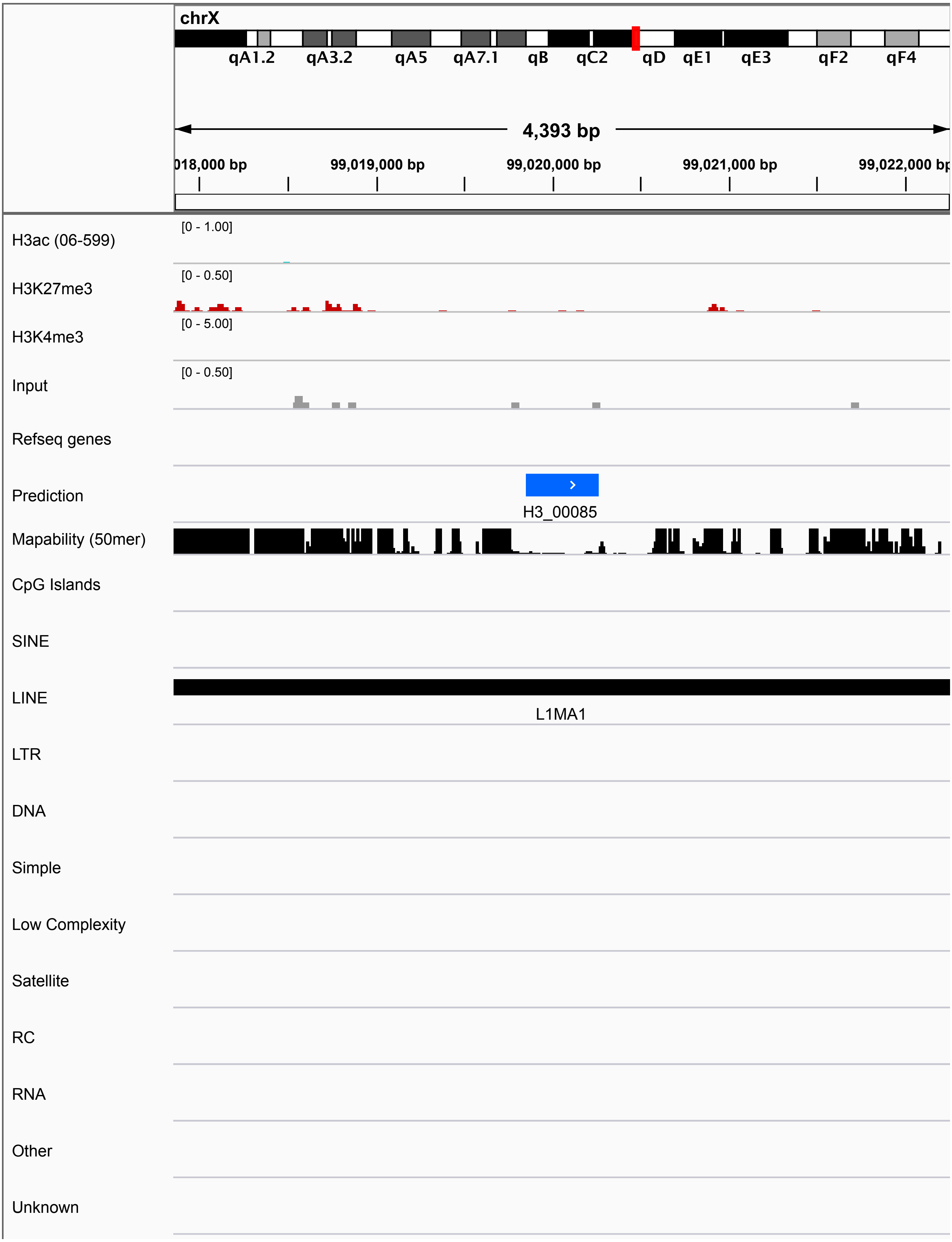

Figure S6

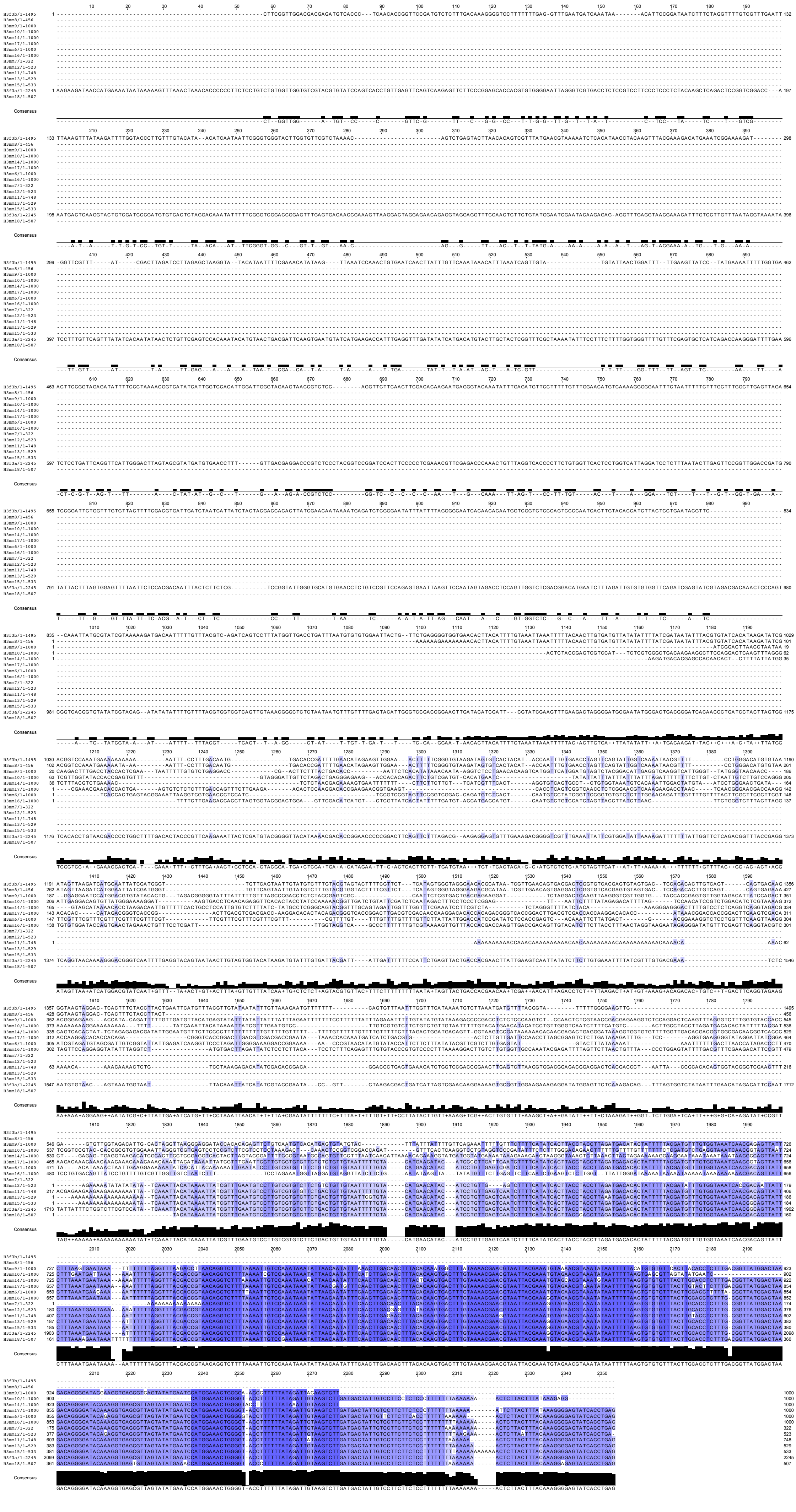

Figure S7

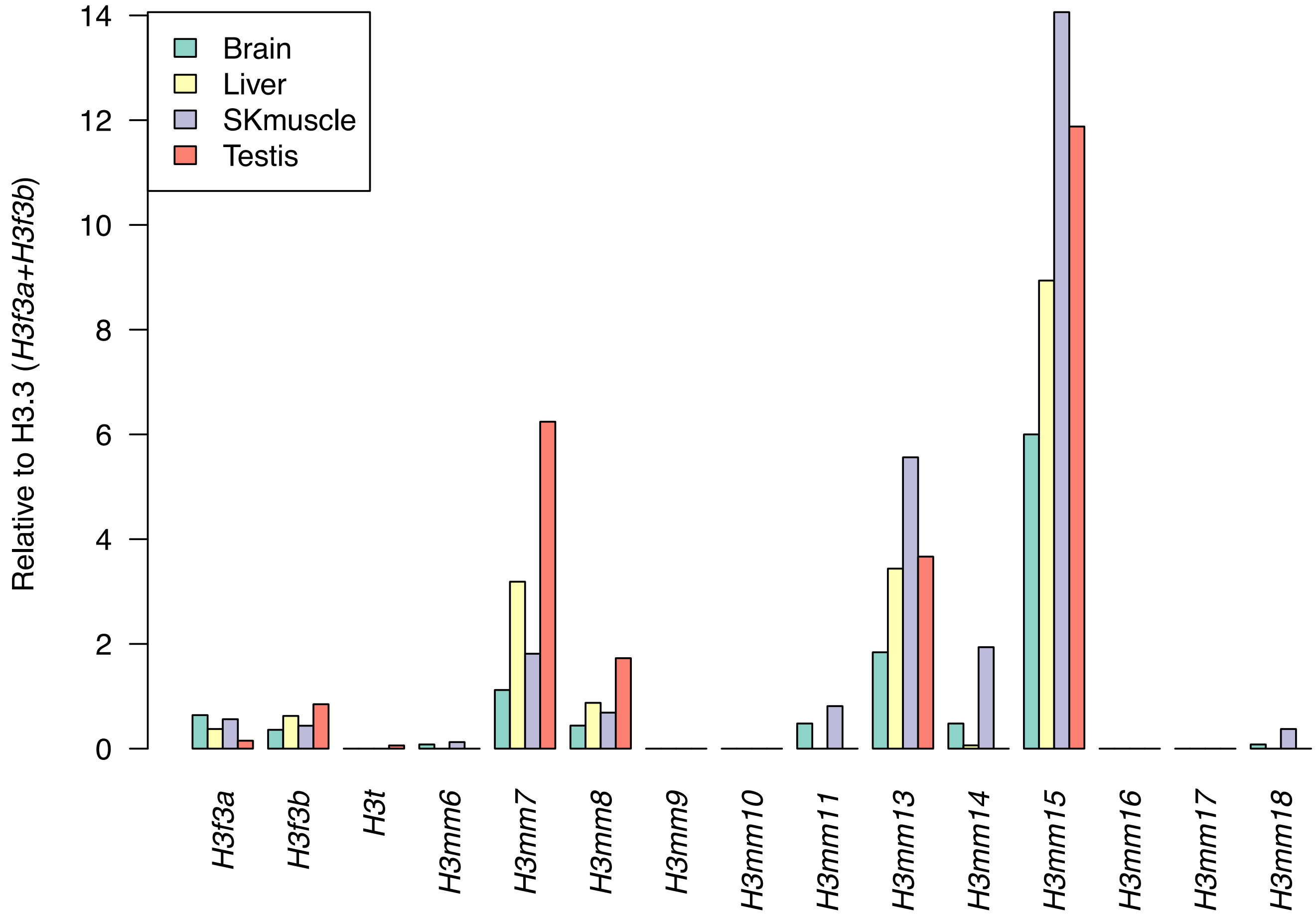

Brain
Liver
SKmuscle
Testis
Relative to H3.3 (*H3f3a+H3f3b*)
0
2
4
6
8
10
12
14
H3f3a
H3f3b
H3t
H3mm6
H3mm7
H3mm8
H3mm9
H3mm10
H3mm11
H3mm13
H3mm14
H3mm15
H3mm16
H3mm17
H3mm18

Figure S8

A

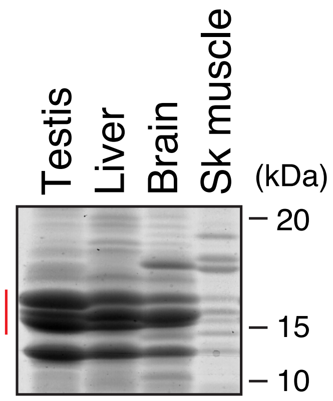

B

H3t: KQLATKVAR (18-26)

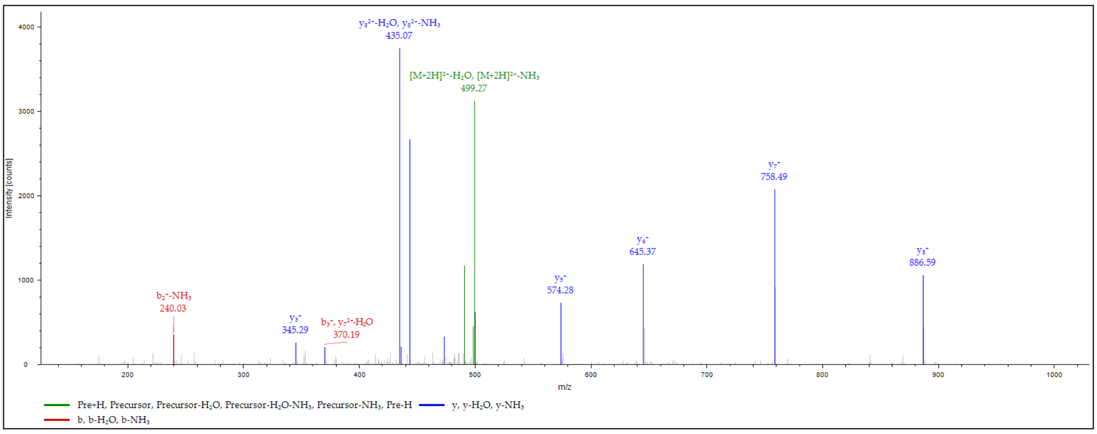

b+	b2+	Seq.	y+	y2+
129.10225	65.05476	K		
257.16083	129.08405	Q	886.54692	443.7771
370.2449	185.62609	L	758.48834	379.74781
441.28202	221.14465	A	645.40427	323.20577
542.3297	271.66849	T	574.36715	287.68721
670.42467	335.71597	K	473.31947	237.16337
769.49309	385.25018	V	345.2245	173.11589
840.53021	420.76874	A	246.15608	123.58168
		R	175.11896	88.06312

C

H3t: YHPGTVALR (41-49)

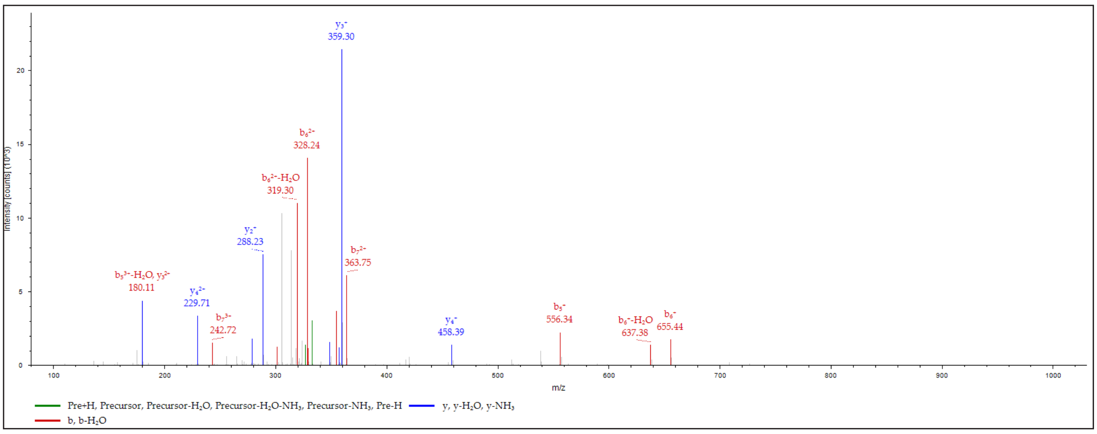

b+	b2+	Seq.	y+	y2+
164.0706	82.53894	Y		
1.12951	151.06839	H	850.4894	425.74834
398.18228	199.59478	P	713.43049	357.21888
455.20375	228.10551	G	616.37772	308.6925
6.25143	278.62935	T	559.35625	280.18176
5.31985	328.16356	V	458.30857	229.65792
726.35697	363.68212	A	359.24015	180.12371
839.44104	420.22416	L	288.20303	144.60515
		R	175.11896	88.06312

D

H3mm7: KSAPSIGGVK (27-36)

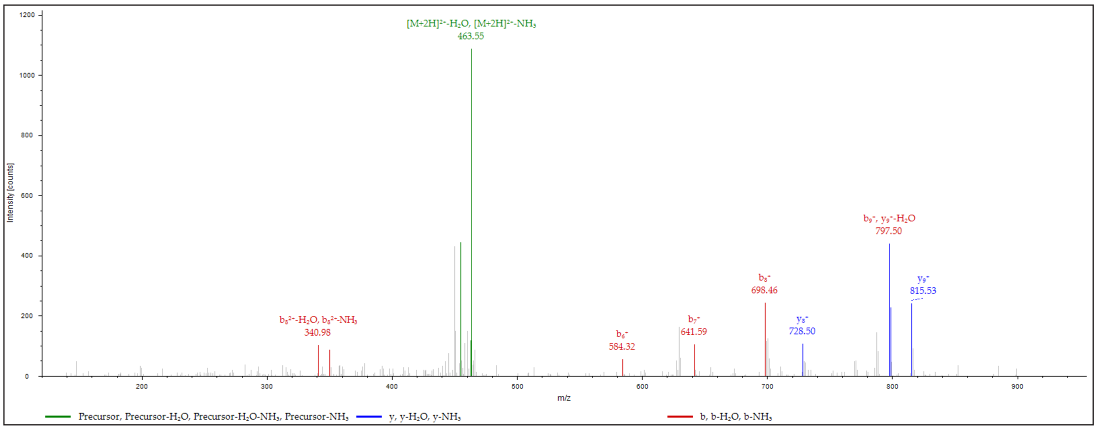

b+	b2+	Seq.	y+	y2+
129.10225	65.05476	K		
216.13428	108.57078	S	815.46219	408.23473
287.1714	144.08934	A	728.43016	364.71872
384.22417	192.61572	P	657.39304	329.20016
471.2562	236.13174	S	560.34027	280.67377
4.34027	292.67377	I	473.30824	237.15776
1.36174	321.18451	G	360.22417	180.61572
8.38321	349.69524	G	303.2027	152.10499
7.45163	399.22945	V	246.18123	123.59425
		K	147.11281	74.06004

Figure S9

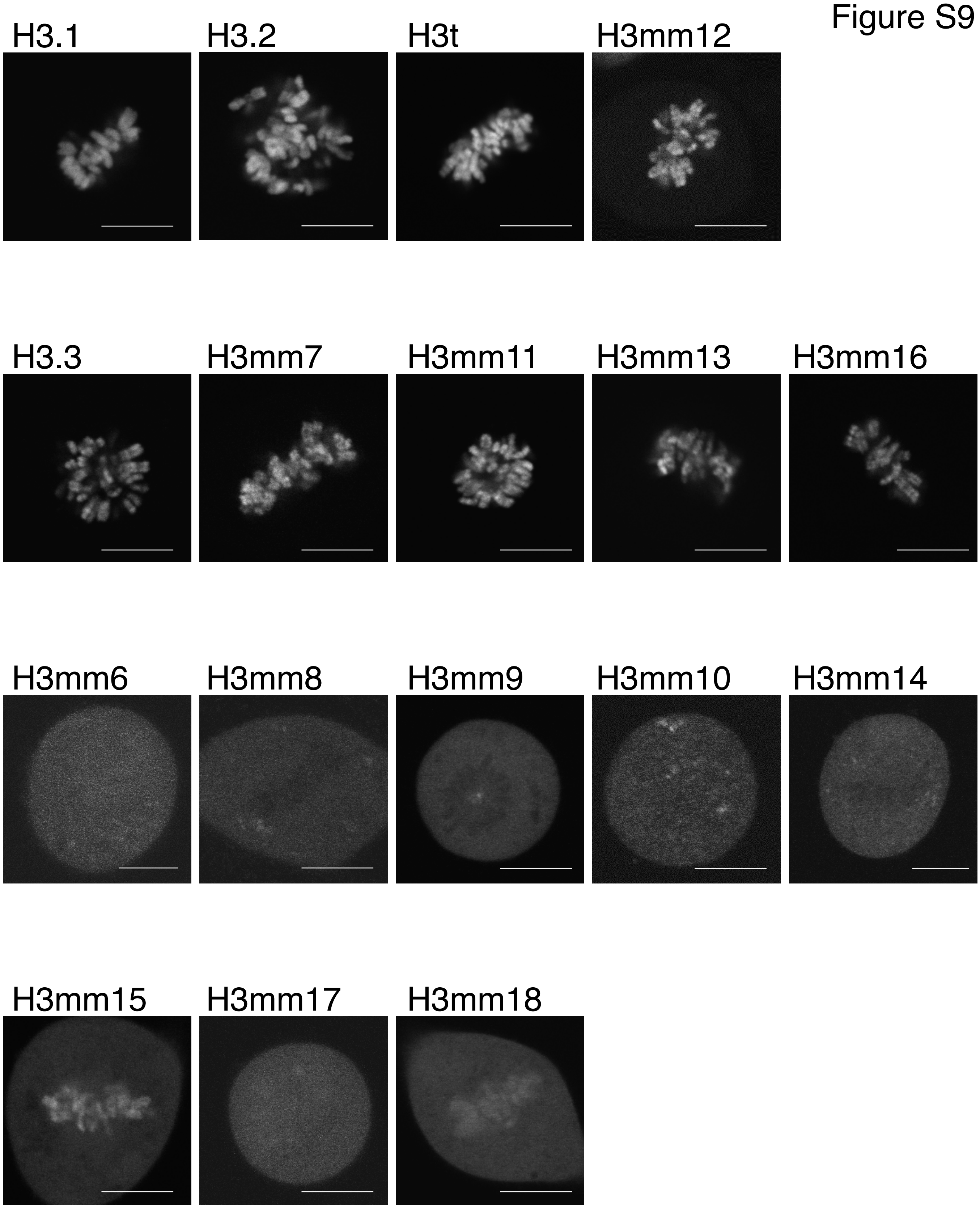

H3.1
H3.2
H3t
H3mm12
H3.3
H3mm7
H3mm11
H3mm13
H3mm16
H3mm6
H3mm8
H3mm9
H3mm10
H3mm14
H3mm15
H3mm17
H3mm18

Figure S10

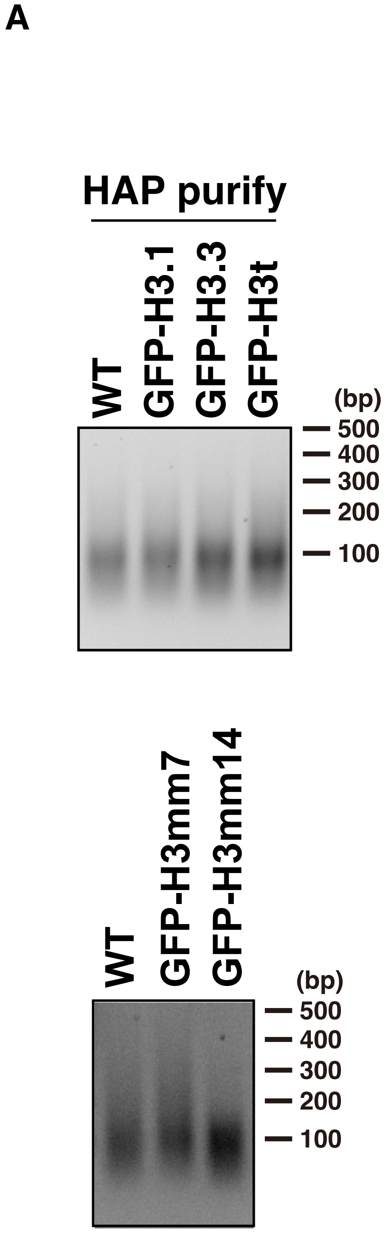
A
HAP purify
WT
GFP-H3.1
GFP-H3.3
GFP-H3t
(bp)
500
400
300
200
100
WT
GFP-H3mm7
GFP-H3mm14
(bp)
500
400
300
200
100

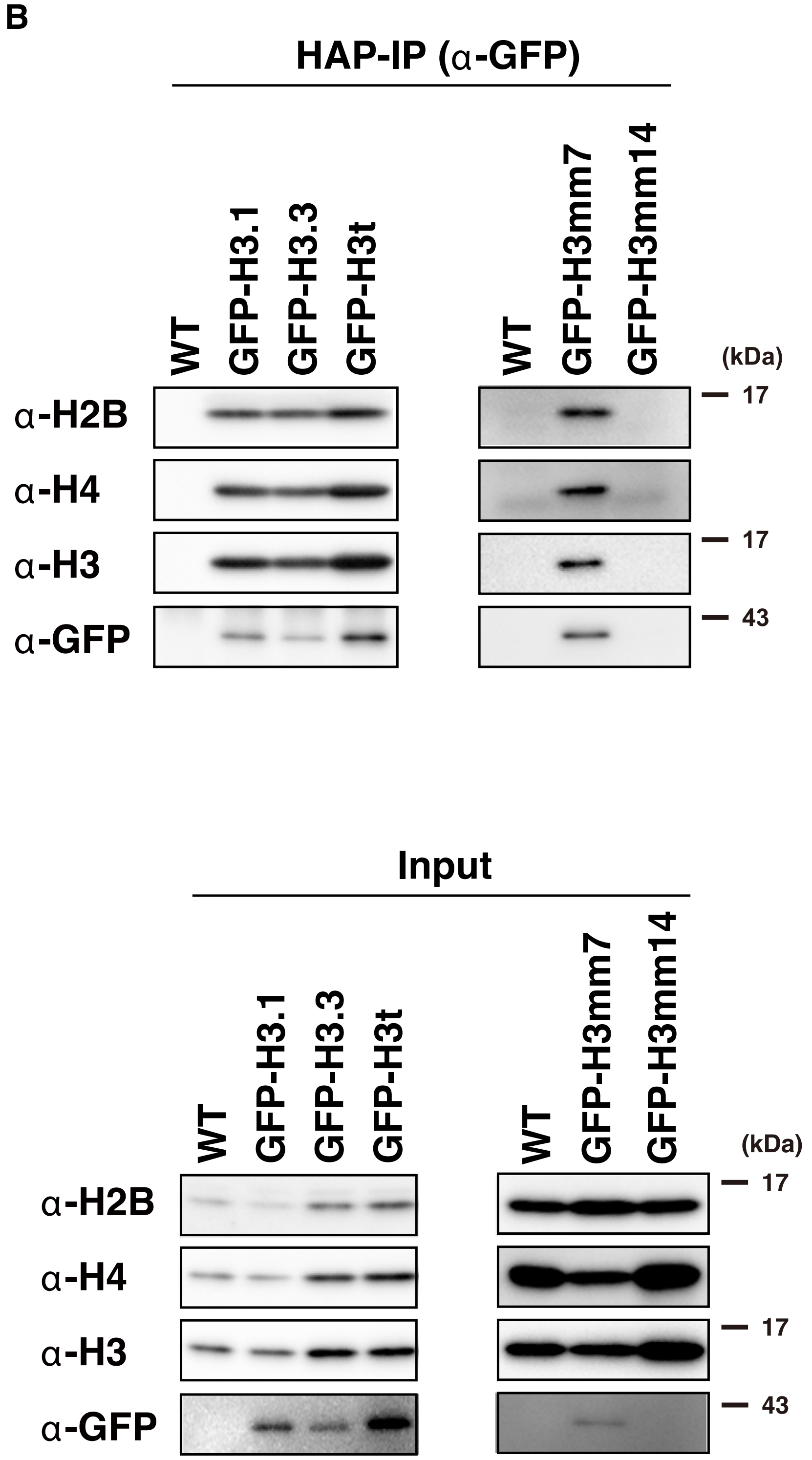
B
HAP-IP (α-GFP)
WT
GFP-H3.1
GFP-H3.3
GFP-H3t
WT
GFP-H3mm7
GFP-H3mm14
(kDa)
α-H2B
α-H4
α-H3
α-GFP
17
17
43
Input
WT
GFP-H3.1
GFP-H3.3
GFP-H3t
WT
GFP-H3mm7
GFP-H3mm14
(kDa)
α-H2B
α-H4
α-H3
α-GFP
17
17
43

Figure S11

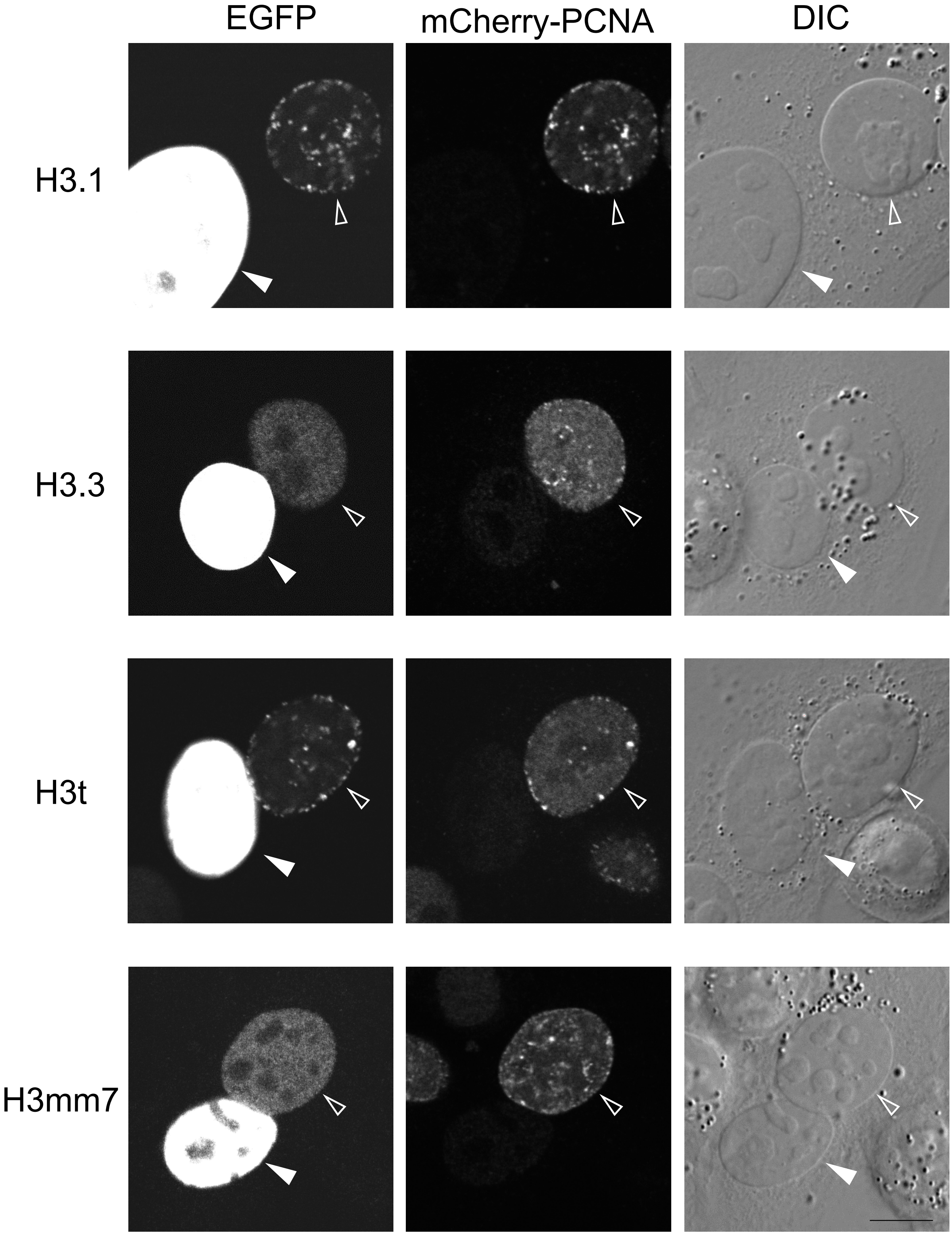

Figure S12

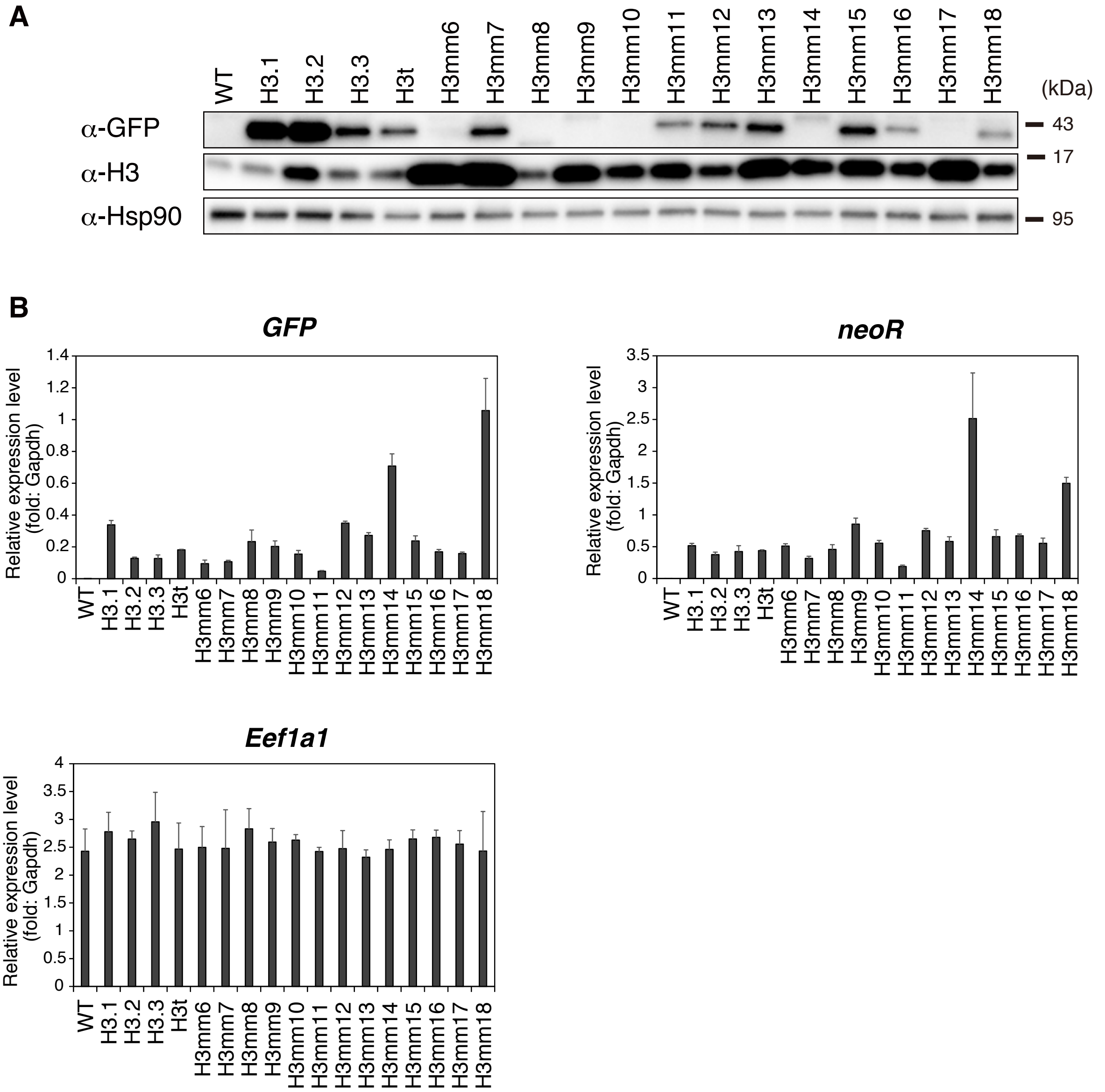

Figure S13

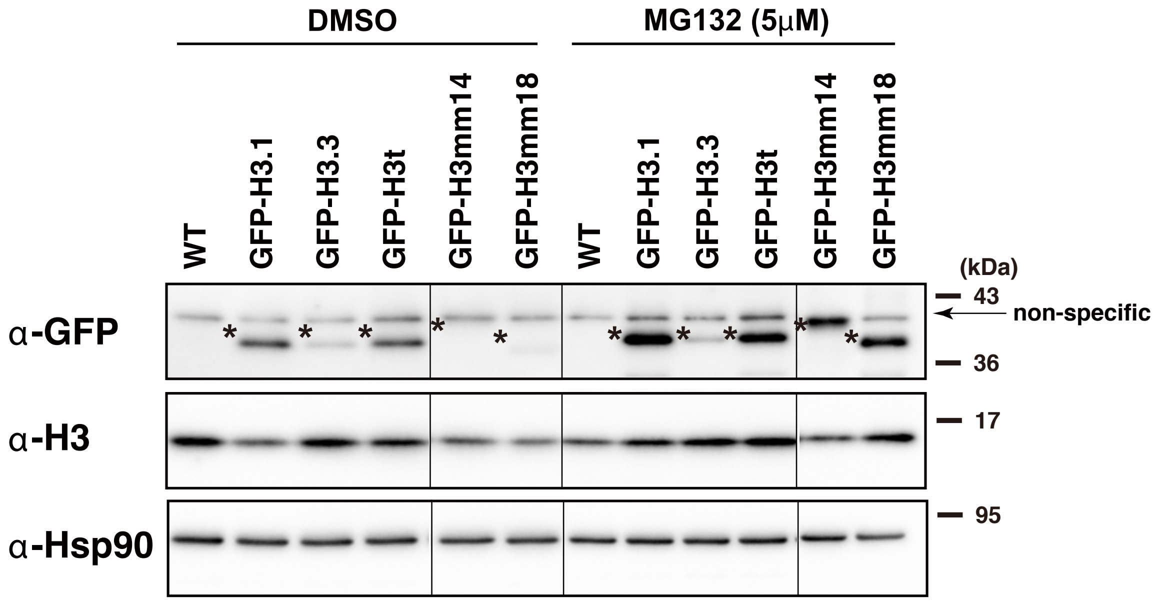

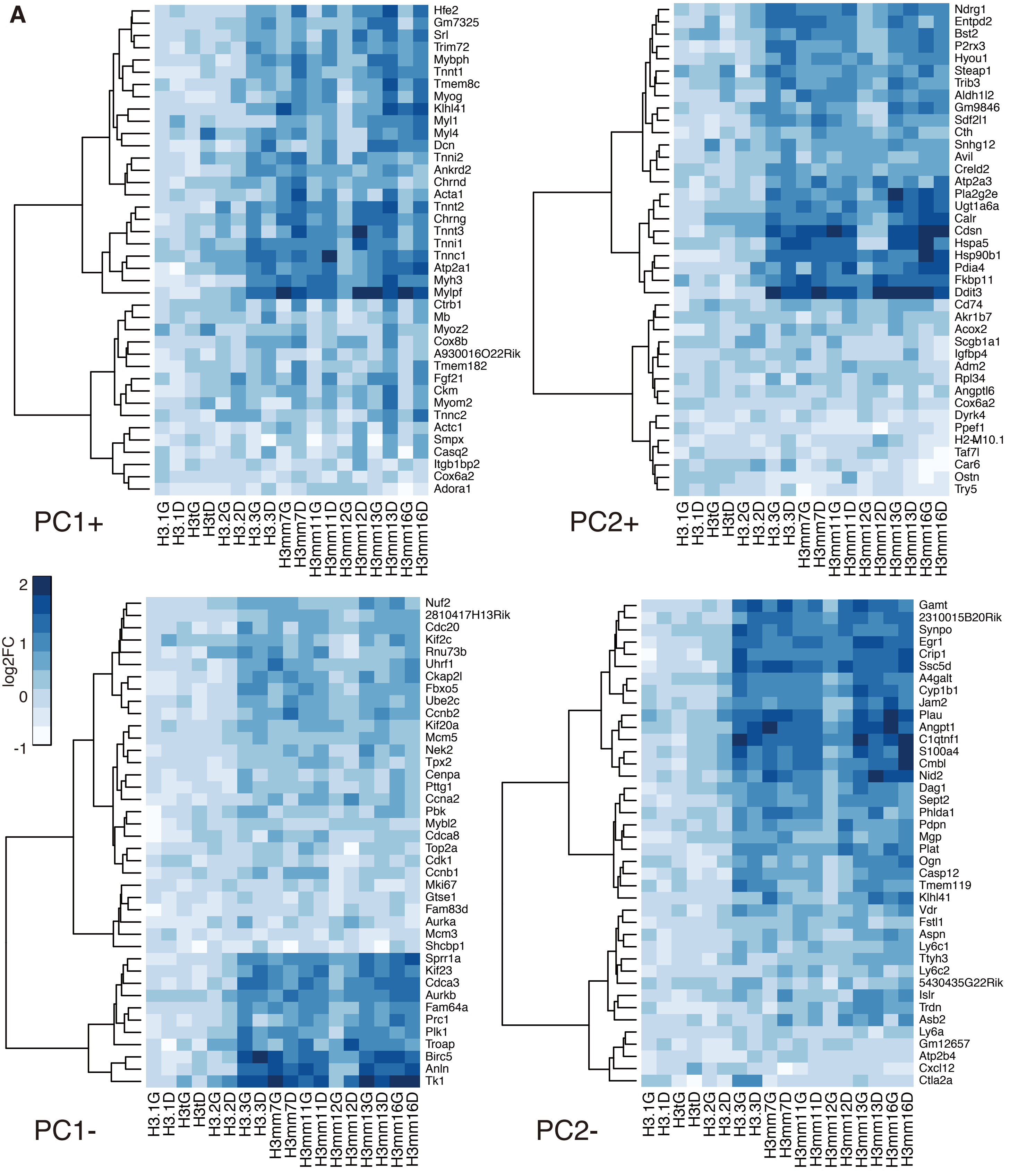
A
PC1+
Hfe2
Gm7325
Srl
Trim72
Mybph
Tnnt1
Tmem8c
Myog
Klhl41
Myl1
Myl4
Dcn
Tnni2
Ankrd2
Chrnd
Acta1
Tnnt2
Chrng
Tnnt3
Tnni1
Tnnc1
Atp2a1
Myh3
Mylpf
Ctrb1
Mb
Myoz2
Cox8b
A930016O22Rik
Tmem182
Fgf21
Ckm
Myom2
Tnnc2
Actc1
Smpx
Casq2
Itgb1bp2
Cox6a2
Adora1
PC2+
Ndrg1
Entpd2
Bst2
P2rx3
Hyou1
Steap1
Trib3
Aldh1l2
Gm9846
Sdf2l1
Cth
Snhg12
Avil
Creld2
Atp2a3
Pla2g2e
Ugt1a6a
Calr
Cdsn
Hspa5
Hsp90b1
Pdia4
Fkbp11
Ddit3
Cd74
Akr1b7
Acox2
Scgb1a1
Igfbp4
Adm2
Rpl34
Angptl6
Cox6a2
Dyrk4
Ppef1
H2-M10.1
Taf7l
Car6
Ostn
Try5
log2FC
2
1
0
-1
PC1-
Nuf2
2810417H13Rik
Cdc20
Kif2c
Rnu73b
Uhrf1
Ckap2l
Fbxo5
Ube2c
Ccnb2
Kif20a
Mcm5
Nek2
Tpx2
Cenpa
Pttg1
Ccna2
Pbk
Mybl2
Cdca8
Top2a
Cdk1
Ccnb1
Mki67
Gtse1
Fam83d
Aurka
Mcm3
Shcbp1
Sprr1a
Kif23
Cdca3
Aurkb
Fam64a
Prc1
Plk1
Troap
Birc5
Anln
Tk1
PC2-
Gamt
2310015B20Rik
Synpo
Egr1
Crip1
Ssc5d
A4galt
Cyp1b1
Jam2
Plau
Angpt1
C1qtnf1
S100a4
Cmbl
Nid2
Dag1
Sept2
Phlda1
Pdpn
Mgp
Plat
Ogn
Casp12
Tmem119
Klhl41
Vdr
Fstl1
Aspn
Ly6c1
Ttyh3
Ly6c2
5430435G22Rik
Islr
Trdn
Asb2
Ly6a
Gm12657
Atp2b4
Cxcl12
Ctla2a
H3.1G H3.1D H3tG H3tD H3.2G H3.2D H3.3G H3.3D H3mm7G H3mm7D H3mm11G H3mm11D H3mm12G H3mm12D H3mm13G H3mm13D H3mm16G H3mm16D

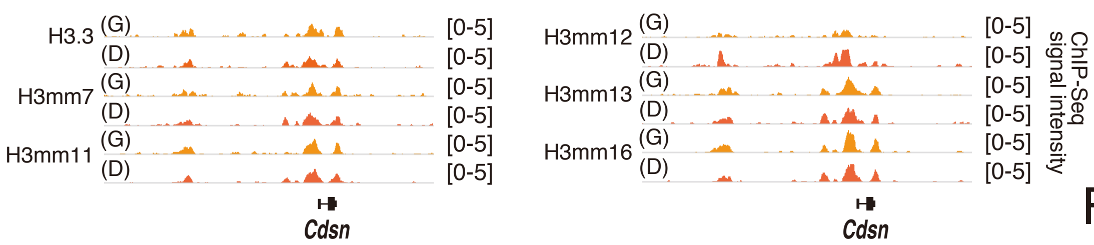
B
H3.3 (G) (D)
H3mm7 (G) (D)
H3mm11 (G) (D)
H3mm12 (G) (D)
H3mm13 (G) (D)
H3mm16 (G) (D)
[0-5]
Cdsn
ChIP-Seq signal intensity

Figure S14
